# Successively Regioselective Electrosynthesis and Electron Transport Property of Stable Multiply Functionalized [60]Fullerene Derivatives

**DOI:** 10.34133/2020/2059190

**Published:** 2020-02-15

**Authors:** Xing-Xing Yan, Bairu Li, Hao-Sheng Lin, Fei Jin, Chuang Niu, Kai-Qing Liu, Guan-Wu Wang, Shangfeng Yang

**Affiliations:** ^1^Hefei National Laboratory for Physical Sciences at Microscale, CAS Key Laboratory of Soft Matter Chemistry, Center for Excellence in Molecular Synthesis of CAS, and Department of Chemistry, University of Science and Technology of China, Hefei, Anhui 230026, China; ^2^Hefei National Laboratory for Physical Sciences at Microscale, CAS Key Laboratory of Materials for Energy Conversion, and Department of Materials Science and Engineering, University of Science and Technology of China, Hefei, Anhui 230026, China; ^3^State Key Laboratory of Applied Organic Chemistry, Lanzhou University, Lanzhou, Gansu 730000, China

## Abstract

With the recent advance in chemical modification of fullerenes, electrosynthesis has demonstrated increasing importance in regioselective synthesis of novel fullerene derivatives. Herein, we report successively regioselective synthesis of stable tetra- and hexafunctionalized [60]fullerene derivatives. The cycloaddition reaction of the electrochemically generated dianions from [60]fulleroindolines with phthaloyl chloride regioselectively affords 1,2,4,17-functionalized [60]fullerene derivatives with two attached ketone groups and a unique addition pattern, where the heterocycle is rearranged to a [5,6]-junction and the carbocycle is fused to an adjacent [6,6]-junction. This addition pattern is in sharp contrast with that of the previously reported biscycloadducts, where both cycles are appended to [6,6]-junctions. The obtained tetrafunctionalized compounds can be successively manipulated to 1,2,3,4,9,10-functionalized [60]fullerene derivatives with an intriguing “*S*”-shaped configuration *via* a novel electrochemical protonation. Importantly, the stability of tetrafunctionalized [60]fullerene products allows them to be applied in planar perovskite solar cells as efficient electron transport layers.

## 1. Introduction

**Figure 1:**
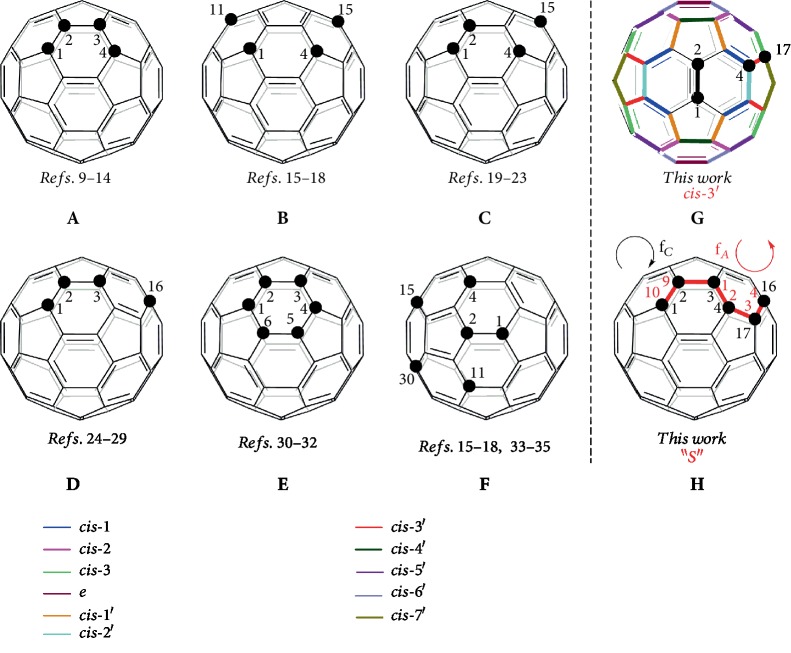
Addition patterns of tetra- and hexafunctionalized [60]fullerene derivatives.

Over the past two decades, functionalized [60]fullerene (C_60_) derivatives have attracted wide attention because of their promising applications in materials, nanotechnology, and biological sciences [1–4]. Particularly, some C_60_ derivatives, represented by [6,6]-phenyl-C_61_-butyric acid methyl ester (PCBM), have exhibited excellent superiors in perovskite solar cells (PSCs) as electron transport layers (ETLs) [5–7]. However, the number of regioisomers increases dramatically with the number of addends and thus causes the problem of tedious chromatographic separation for individual regioisomer. Only one isomeric monocycloadduct is usually formed, up to 8 regioisomeric biscycloadducts have been isolated from cycloaddition reactions of C_60_ [8]. On the other hand, the regioisomers of tetrafunctionalized C_60_ derivatives reported most commonly are 1,2,3,4-isomers (**A**) [9–14], 1,4,11,15-isomers (**B**) [15–18], 1,2,4,15-isomers (**C**) [19–23], and 1,2,3,16-isomers (**D**) [24–29], while for the hexafunctionalized C_60_ derivatives, the most frequently encountered regioisomers are 1,2,3,4,5,6-isomers (**E**) [30–32] and 1,2,4,11,15,30-isomers (**F**) [15–18, 33–35] (Figure 1). Although the elegant templated multifunctionalizations of fullerenes have been devised to realize high regioselectivity [8, 36], the regiocontrol on the formation of a specific isomer of biscycloadducts and multicycloadducts is still a daunting task.

The electrophilic-to-nucleophilic reactivity reversal of fullerenes and their derivatives caused by electrochemical reduction opens a new territory in fullerene chemistry and has demonstrated increasing importance in the efficient synthesis of novel fullerene derivatives [37], including the abovementioned types **A**, **C**, and **D** [11, 19, 25–29]. It was interestingly found that the same dianionic [60]fulleroindoline (**1a**^2–^, *vide infra*) behaved differently toward alkylating and acylating reagents. For example, the reaction of **1a**^2–^ with benzyl bromide gave both mono- and dibenzylated 1,2,3,16-adducts [25], while its reaction with benzoyl chloride could afford only monoacylated 1,2,3,16-adducts [28]. The attempted synthesis of diacylated 1,2,3,16-adducts failed and remains challenging. In our recent work [38], we disclosed the synthesis of tetra- and hexafunctionalized [60]fullerene derivatives with unprecedented addition patterns from the reaction of **1a**^2–^ with 1,2-bis(bromomethyl)benzene. Even though we could capture the intermediate leading to the cyclized 1,2,4,17-adduct, the instability of this unique cyclized product precluded its characterization by ^13^C NMR and single-crystal X-ray analysis. Bearing the aforementioned different reactivity of the same dianionic species toward alkylating and acylating reagents and instability of the previously obtained tetrafunctionalized product in mind, our continuous efforts in electrochemical functionalization of fullerene derivatives [25, 28, 29, 38–41] stimulated us to investigate the reaction of the dianionic [60]fulleroindolines **1a**–**c**^2–^ with phthaloyl chloride in order to contrast their reactivity behavior and to see if the unprecedented tetrafunctionalized *cis*-3′ isomers (type **G**) and “*S*”-shaped hexafunctionalized products (type **H**) bearing two acyl groups can be generated in the present case [42]. It turns out that these two unique types of tetra- and hexafunctionalized products can be successfully synthesized and are stable up to 107−275°C. Intriguingly, the reactivity behaviors of **1a**^2–^ toward 1,2-bis(bromomethyl)benzene and phthaloyl chloride are quite different, and chemical properties of their anionic tetrafunctionalized products also behave divergently. Importantly, the stability of the current tetrafunctionalized products allows them to be utilized in planar perovskite solar cells so as to investigate their electron transport properties.

## 2. Results and Discussion

### 2.1. Electrosynthesis of Tetra- and Hexafunctionalzied Fullerene Derivatives

**Figure 2:**
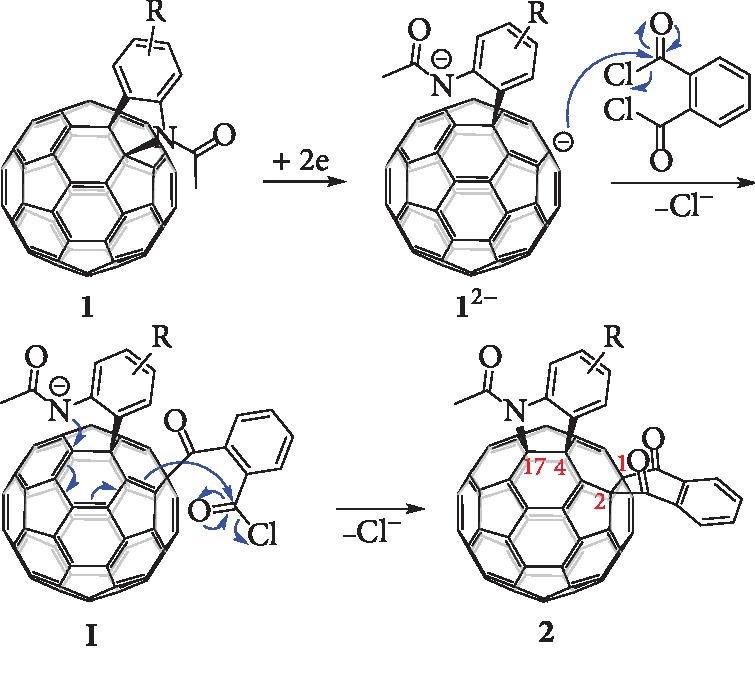
Synthetic design for the unprecedented *cis*-3′ regioisomer.

The dianionic species of [60]fulleroindolines **1** can be obtained by controlled potential electrolysis (CPE) and have ring-opened structures after acceptance of two electrons [25, 28]. For these ring-opened structures, the most negatively charged carbon atom among the fullerene skeleton is located at the *para* position of the aryl substituents. The reaction of **1**^2–^ with acyl chlorides proceeded *via* a S_N_2 rather than a SET process [28]. Therefore, we surmise that if phthaloyl chloride is chosen to react with **1**^2–^, a similar S_N_2 pathway would afford the anionic intermediate **I**, followed by the intramolecular S_N_2 ring-closure process *via* C–N bond formation [25, 28] to generate **2** with the heterocycle rearranged to a [5,6]-junction and the carbocycle anchored to a [6,6]-junction (Figure 2). It is noteworthy that our thus designed 1,2,4,17-functionalized C_60_ derivatives **2** have a unique *cis*-3′ addition pattern (Figure 1), which is in sharp contrast with that for the reported typical *cis*-3 adducts where both cycloadditions occur at [6,6]-junctions [8].

**Figure 3:**
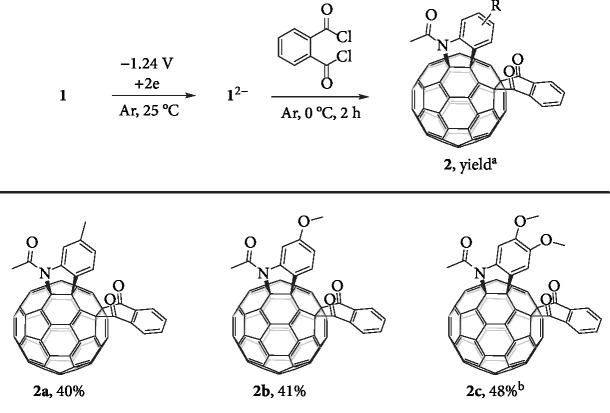
Reaction of the electrochemically generated **1**^2–^ with phthaloyl chloride. Unless otherwise specified, all the reactions were performed with 0.02 mmol of **1**^2–^ and 0.4 mmol of phthaloyl chloride in 25 mL of ODCB at 0°C for 2 h under an argon atmosphere. ^a^Isolated yield. ^b^10 equiv. of phthaloyl chloride.

[60]Fulleroindolines **1a**–**c** were synthesized according to our reported procedure [43]. The cyclic voltammograms (CVs) of **1a**–**c** were very similar and showed an irreversible second redox process (Figures [Supplementary-material supplementary-material-1]), hinting that the C–N bond cleavage occurred after receiving two electrons [25–29, 38]. It turned out that the reaction of the dianionic species of **1a**–**c** with phthaloyl chloride indeed afforded the desired *cis*-3′ regioisomers **2a**–**c**. The cyclization of **1a**^2–^, which was obtained by CPE at –1.24 V *vs.* SCE, with phthaloyl chloride was chosen to screen the optimal reaction conditions (for details, see the text and [Supplementary-material supplementary-material-1] in the Supplementary Materials). It was found that the reaction of **1a**^2–^ with 20 equiv. of phthaloyl chloride in *ortho*-dichlorobenzene (ODCB) at 0°C for 2 h generated **2a** in 40% yield. Similarly, the employment of substrate **1b** bearing one methoxy group on the phenyl ring and substrate **1c** containing two methoxy groups on the phenyl ring afforded **2b** and **2c** in 41% and 48% yields, respectively (Figure 3).

**Figure 4:**
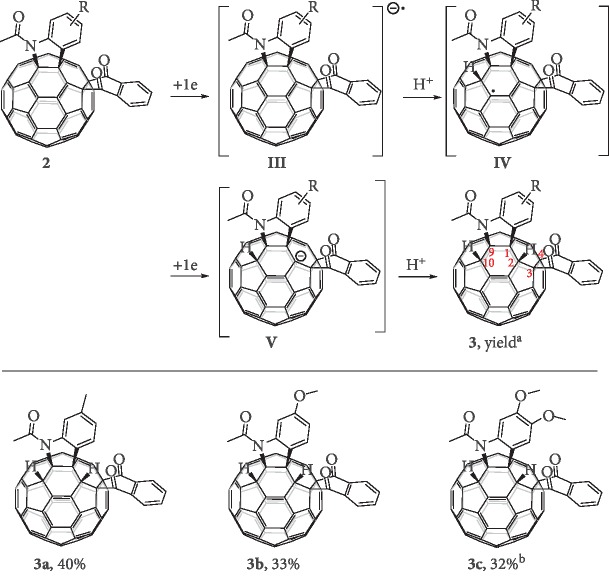
Syntheses of **3a**, **3b**, and **3c**. Unless otherwise specified, all the reactions were performed with 0.01 mmol of **2**, 0.01 mmol of TFA, and CPE at –1.20 V, at 25°C under an argon atmosphere. ^a^Isolated yield. ^b^CPE at –1.13 V.

Both the first and second redox processes in the CVs of **2a**–**c** were irreversible (Figures [Supplementary-material supplementary-material-1]), suggesting that they could be further electrochemically derivatized. In an attempt to obtain the hexafunctionalized fullerene derivative by protonation of **2a**^2–^ generated from **2a** by CPE at –1.20 V with trifluoroacetic acid (TFA), only the protonated 1,2,3,4-adduct **IIa** [44] of **1a**^2–^ was isolated due to the facile deacylation of the dianionic species and fast concomitant protonation under our conditions. Fortunately, we discovered that with the addition of 1 equiv. of TFA before electroreduction of **2a**, the hexafunctionalized fullerene derivative **3a** was isolated in 40% yield along with a trace amount of **IIa**. Further increasing the amount of TFA was detrimental to the product yield. Similarly, products **3b** and **3c** could be obtained by the electrochemical protonation of **2b** and **2c** in 33% and 32% yields, respectively (Figure 4). It is believed that product **3** is generated by a highly efficient process of stepwise one-electron reduction and protonation of **2** to give intermediates **III** and **IV**, followed by another one-electron reduction to afford **V** and final protonation (Figure 4). Alternatively, **3** might be generated by a reversal of the sequence as shown in Figure 4 with the first protonation at the carbon atom next to the ketone group. The success for the formation of **3** is probably ascribed to that the presence of TFA facilitates the sequential one-electron reduction and concomitant protonation and thus prohibits the deacylation.

It is worthwhile and illustrative to compare the different reactivity behaviors of the dianionic [60]fulleroindoline **1a**^2–^ toward the present phthaloyl chloride and the previously investigated 1,2-bis(bromomethyl)benzene and to contrast the physical and chemical properties of the formed multiply functionalized fullerene derivatives [38]. A monoalkylated 1,2,3,16-adduct, which verified the assumed addition preference at the *para* position of the aryl substituent, could be isolated if the reaction of **1a**^2–^ with 1,2-bis(bromomethyl)benzene proceeded for a short time (10 min, 0°C) and then quenched with TFA. In contrast, the attempts to intercept the anionic intermediate **Ia** with TFA failed, reflecting that the ring-closure process of **Ia** was highly rapid to generate 1,2,4,17-adduct **2a**. Unlike 1,2-bis(bromomethyl)benzene, phthaloyl chloride reacted with **1a**^2–^ at higher temperature afforded only stable **2a**, while the isomeric 1,2,3,4-adduct could not be identified. Product **2a** was thermally stable up to 168°C ([Supplementary-material supplementary-material-1]), yet the tetrafunctionalized product from 1,2-bis(bromomethyl)benzene was unstable, tended to decompose, and was partially rearranged to the more stable isomeric 1,2,3,4-adduct. Another difference between these two counterparts was that the acid TFA must be added before the electroreduction of the tetrafunctionalized **2a** to successfully form the hexafunctionalized **3a** due to the fast deacylation of **2a**^2–^, while TFA could be added as the proton source after the generation of the dianonic tetrafunctionalized product from 1,2-bis(bromomethyl)benzene.

### 2.2. Characterizations

**Figure 5:**
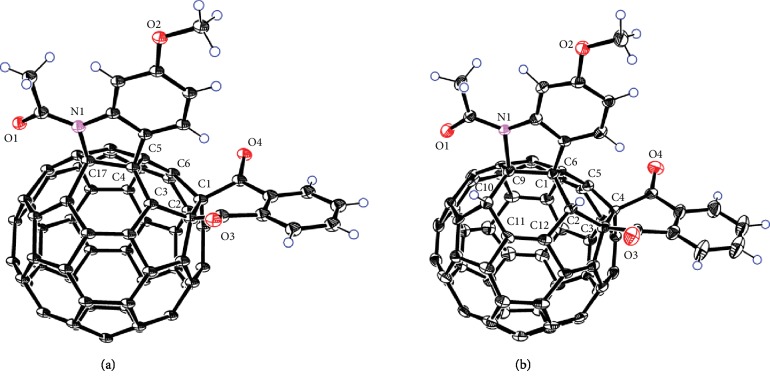
(a) ORTEP diagram of one enantiomer of **2b** with thermal ellipsoids shown at 50% probability. (b) ORTEP diagram of one enantiomer of **3b** with thermal ellipsoids shown at 50% probability. The chloroform molecules are omitted for clarity.

All new products **1b**, **1c**, **2a**–**c**, and **3a**–**c** were fully characterized by MALDI-TOF HRMS, ^1^H NMR, ^13^C NMR, FT-IR, and UV-Vis spectroscopies. Particularly, the two doublets around 6 ppm with a coupling constant of 2.3 Hz in the ^1^H NMR spectra of products **3a**–**c** indicated that they contained two fullerenyl protons in 1,4-arrangement [44]. The HMBC spectrum of **3a** clearly showed that the proton (6.09 ppm) at C2 (57.04 ppm) correlated with C1 (61.02 ppm) and C3 (68.32 ppm) and that the proton (6.02 ppm) at C10 (56.03 ppm) correlated with C9 (80.56 ppm) (Figures [Supplementary-material supplementary-material-1]), indicating that these protons and carbons were adjacent. Furthermore, the assigned structures of **2b** and **3b** were established by the single-crystal X-ray diffraction analyses (Figure 5). This is the first time that the assignment of 1,2,4,17-adducts was confirmed by single-crystal structure.

The single crystal of **2b** was obtained through slow diffusion of methanol into a chloroform solution of **2b** at 4°C. Figure 5(a) displays the X-ray single-crystal diagram for one of the two enantiomers (0.5 : 0.5) of **2b**, where a heterocycle is bonded to C_60_ through a C_aryl_ atom and a N atom at C4 and C17 sites, respectively, and two ketone groups are attached to C1 and C2 sites, respectively. The four functionalized fullerene carbon atoms are uplifted from the spherical surface notably because of their sp^3^ characters with the bond lengths of 1.560(16) Å and 1.594(10) Å for the C1–C2 and C4–C17 bonds, respectively. The bond lengths for C2–C3, C3–C4, and C1–C6 are 1.559(14) Å, 1.444(12) Å, and 1.509(15) Å, respectively, which are within the range of typical C–C single bond lengths, whereas the C5–C6 bond has a bond length of 1.380(15) Å, thus possessing double bond character. The resolved single-crystal structure unambiguously demonstrates that the molecular structure of the obtained 1,2,4,17-adduct has the *cis*-3′ addition pattern. The single crystal of **3b** was obtained through slow evaporation of a chloroform solution of **3b** at 4°C. The X-ray single-crystal diagram for one of the two enantiomers (0.5 : 0.5) of **3b** are illustrated in Figure 5(b) and resembles that of **2b** except that two additional hydrogen atoms are attached to C2 and C10 atoms. These two carbon atoms bearing hydrogen atoms are also uplifted from the spherical surface notably because of their sp^3^ characters with the bond lengths of 1.613(7) Å and 1.556(10) Å for the C1–C2 and C9–C10 bonds, respectively. The bond lengths of 1.646(7) Å, 1.601(13) Å, and 1.579(10) Å for C1–C9, C2–C3, and C3–C4, respectively, indicate that they are C–C single bonds; meanwhile, bond lengths for C5–C6 and C11–C12 are 1.358(10) Å and 1.363(11) Å, thus showing double bond character. Intriguingly, the resolved single-crystal structure unequivocally reveals that the molecular structure of the obtained 1,2,3,4,9,10-adduct [45] has a unique “*S*”-shaped configuration.

### 2.3. Applications in Perovskite Solar Cells

**Figure 6:**
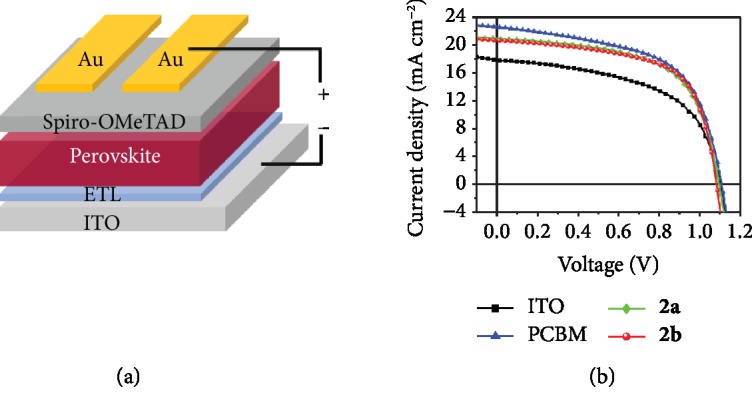
(a) Schematic structure of the n-i-p PSC device with or without (w/o) fullerene derivative as the ETL. (b) *J*−*V* curves of the n-i-p PSC devices without ETL and with PCBM, **2a**, and **2b** as the ETL measured under illumination of an AM 1.5 solar simulator (100 mW cm^−2^) in air. The scanning direction was from open-circuit voltage to short-circuit (reverse), and the scan speed was 100 mV s^−1^.

Although products **2a**–**c** and **3a**–**c** bear a heterocycle fused to a [5,6]-junction of C_60_, they are thermally stable up to 107−275°C, as determined by thermogravimetric analyses (TGA) (Figures [Supplementary-material supplementary-material-1]). Given that fullerene derivatives such as PCBM have strong electron-accepting ability and thus have been popularly applied as ETLs of planar PSCs [46, 47], we next applied two representative highly soluble fullerene products **2a** and **2b** as novel ETLs of regular-structure (n-i-p) PSC devices with configurations of ITO/ETL/Cs_0.05_FA_0.83_MA_0.12_PbI_2.55_Br_0.45_ perovskite/Spiro-OMeTAD/Au, in which 2,2′,7,7′-tetrakis(*N,N*-di-*p*-methoxyphenylamine)-9,9′-spirobifluorene (Spiro-OMeTAD) was used as the hole transport material (Figure 6(a)). For comparison, devices without ETL and with commonly used PCBM ETL were also fabricated [46, 47]. The current density-voltage (*J−V*) curves of the PSC devices based on different ETLs with optimized thicknesses measured under one sun illumination are shown in Figure 6(b), and the measured photovoltaic parameters, including open-circuit voltage (*V*_oc_), short-circuit current (*J*_sc_), fill factor (FF), power conversion efficiency (PCE), series resistance (*R*_s_), and shunt resistance (*R*_sh_) of the best performance devices, are summarized in Table 1. The control device without ETL showed a *V*_oc_ of 1.10 V, a *J*_sc_ of 17.92 mA cm^−2^, an FF of 54.71%, and a PCE of 10.77%. When **2a** and **2b** were incorporated as ETLs, the device performance enhanced obviously. The **2a**-based device exhibited an increased PCE of 13.81%, calculated from a *V*_oc_ of 1.09 V, a *J*_sc_ of 20.82 mA cm^−2^, and an FF of 60.65%. Upon using **2b** as ETL, PCE of the device increased further to 14.04%, which approached that of the PCBM-based device (14.49%). These results showed the considerably good electron transport properties of **2a** and **2b**.

**Figure 7:**
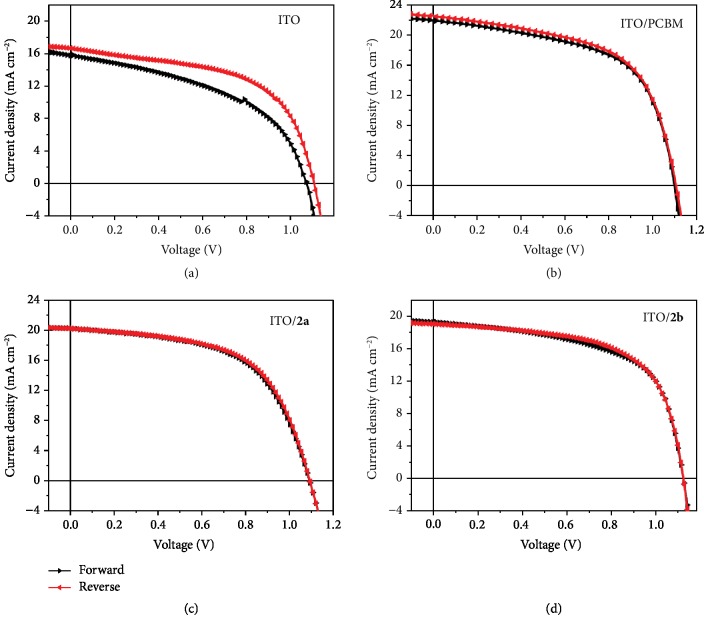
The *J*-*V* curves of the PSCs using different fullerene derivatives, (a) without ETL, (b) PCBM, (c) **2a**, and (d) **2b** as the ETL in different scan directions.

It is known that n-i-p PSC devices based on the conventional TiO_2_ ETL usually suffer from severe current-voltage hysteresis [7]. To examine the hysteresis of *J*−*V* curves of our devices based on **2a** or **2b** ETL, we measured the *J*−*V* curves in different scan directions (Figure 7), and the corresponding device parameters are given in Table 2. The control device without ETL showed severe hysteresis with a hysteresis index, defined as [PCE(reverse) − PCE(forward)]/PCE(reverse) [48], of 20.6%. Upon incorporating **2a** or **2b** ETL, the device exhibited negligible hysteresis with a small hysteresis index of 1.80% or 1.51%, respectively. This is similar to the case of PCBM ETL. Such a dramatic suppression of the hysteresis may come from the improved electron transport due to the strong electron-accepting ability of **2a** and **2b**, resulting in suppressed charge accumulation at the perovskite/ITO interface [49, 50]. Therefore, these results along with the comparable PCE to PCBM reveal the promising applications of **2a** and **2b** in PSCs.

## 3. Conclusion

In summary, we have achieved an efficient and regioselective synthesis of the diacylated products of fulleroindolines **1a–c** by the reaction of the electrochemically generated dianionic **1a–c**^2–^ with phthaloyl chloride. The obtained tetrafunctionalized fullerene products are 1,2,4,17-adducts **2a–c** and have a unique *cis*-3′ addition pattern where the heterocycle is rearranged to a [5,6]-junction and the carbocycle is appended to a [6,6]-junction. Intriguingly, 1,2,4,17-adducts can be successively protonated to provide hexafunctionalized fullerene products **3a–c** by a stepwise one-electron reduction and protonation of **2a–c**, which are 1,2,3,4,9,10-adducts and possess an intriguing “*S*”-shaped addition pattern. The tetra- and hexafunctionalized fullerene derivatives have been fully characterized by spectroscopic data and single-crystal X-ray diffraction analyses. Both **2a–c** and **3a–c** are stable up to 107−275°C, and representative fullerene products show considerably good electron transport performance in planar perovskite solar cells. This study paves the way to regiocontrolled synthesis of novel multifunctionalized fullerene derivatives toward applications in energy conversion.

## 4. Materials and Methods

### 4.1. General Procedure for Synthesis of ***2a***–***c***

The dianionic **1**^2–^ was obtained by electroreduction from [60]fulleroindoline **1** (0.02 mmol) at –1.24 V by CPE and then reacted with phthaloyl chloride (0.40 mmol or 0.20 mmol). After being stirred at 0°C for 2 h, the resulting mixture was directly filtered through a silica gel (200–300 mesh) plug with CS_2_/CH_2_Cl_2_ (1 : 1 *v*/*v*) to remove the supporting electrolyte and insoluble materials and then evaporated in vacuo to remove the solvent. Next, the residue was further separated on a silica gel column (300–400 mesh) with CS_2_/CH_2_Cl_2_ as the eluent to afford **2** as an amorphous brown solid along with unreacted **1**.

### 4.2. General Procedure for Synthesis of ***3a***–***c***

The mixture of **2** (0.01 mmol) and TFA (0.74 *μ*L, 0.01 mmol) was dissolved in ODCB containing 0.1 M TBAP and then electroreduced by CPE at –1.20 V or –1.13 V. The potentiostat was turned off after the theoretical coulomb was reached. The resulting mixture was directly filtered through a silica gel (200–300 mesh) plug with CS_2_/CH_2_Cl_2_ (1 : 1 *v*/*v*) to remove the supporting electrolyte and insoluble materials and then evaporated in vacuo to remove the solvent. Next, the residue was further separated on a silica gel column (300–400 mesh) with CS_2_/CH_2_Cl_2_ as the eluent to afford **3** as an amorphous red-brown solid along with a minor byproduct **II**.

### 4.3. Device Fabrication of Perovskite Solar Cells

The patterned ITO-coated glass was cleaned by sequential ultrasonic treatment in detergent, deionized water, acetone, and isopropanol for 15 min and then treated with ultraviolet-ozone for 20 min. The PCBM (20 mg mL^−1^ in ODCB) or representative fullerene derivative (**2a** and **2b**, saturated solution in ODCB) was deposited on the ITO substrates by spin coating at 2000 rpm for 60 s. The as-spun films were annealed at 100°C for 10 min. Next, Cs_0.05_FA_0.83_MA_0.12_PbI_2.55_Br_0.45_ perovskite precursor solution (1.3 M dissolved in dimethyl sulfoxide and *N,N*-dimethylformamide with a volume ratio of 2 : 8, with molar ratios of PbI_2_/PbBr_2_, 1.1 : 0.2; FAI : MABr, 1 : 0.2; CsI/(FAI+MABr), 0.05 : 0.95; PbI_2_/FAI, 1.1 : 1; and PbBr_2_ : MABr, 1 : 0.2) was spin-coated onto the substrates with a two-step procedure. The first step was 2000 rpm for 10 s with an acceleration of 200 rpm. The second step was 6000 rpm for 30 s with an acceleration of 2000 rpm. At 15 s before the end of the second procedure, 100 *μ*L chlorobenzene (CB) was dropped on the spinning substrate. The substrate was then immediately transferred on a hotplate and heated at 100°C for 60 min. After the perovskite films were cooled down to room temperature, the hole transport layer was deposited on top of the perovskite film by spin coating at 3000 rpm for 30 s using a CB solution which contained 73.2 mg mL^−1^ of Spiro-OMeTAD and 28.8 *μ*L mL^−1^ of tert-butylpyridine, as well as 18.8 *μ*L mL^−1^ of bis(trifluoromethane)sulfonimide lithium salt (Li-TFSI, 520 mg mL^−1^ in acetonitrile). Finally, the device was transferred into a vacuum chamber (10^−6^ torr), and an Au electrode (ca. 55 nm thick) was thermally deposited through a shadow mask to define the effective active area of the device (0.10 cm^2^).

## Figures and Tables

**Table 1:** Device parameters of PSCs without ETL and with **2a**, **2b**, and PCBM as ETLs.

ETL	*V*_oc_ (V)	*J*_sc_ (mA cm^−2^)	FF	PCE (%)	*R*_s_ (Ω cm^2^)	*R*_sh_ (Ω cm^2^)
—	1.10	17.92	54.71	10.77	7.7	297.6
**2a**	1.09	20.82	60.65	13.81	6.4	515.4
**2b**	1.08	20.56	63.12	14.04	5.0	487.1
PCBM	1.11	22.45	58.37	14.49	6.0	304.2

**Table 2:** Photovoltaic parameters of the best performance devices without ETL and with **2a**, **2b**, and PCBM as ETL in different scan directions.

ETL	Direction	*V*_*oc*_ (V)	*J*_*sc*_ (mA cm^−2^)	FF (%)	PCE (%)	Hysteresis index (%)
—	Reverse	1.11	16.58	56.49	10.36	20.60%
	Forward	1.07	15.81	48.53	8.23	

**2a**	Reverse	1.09	20.20	57.90	12.78	1.80%
	Forward	1.09	20.22	56.89	12.55	

**2b**	Reverse	1.12	19.05	61.94	13.26	1.51%
	Forward	1.12	19.30	60.17	13.06	

PCBM	Reverse	1.11	22.45	58.37	14.49	2.40%
	Forward	1.10	21.86	58.90	14.14	

## Supplementary Materials

Supplementary Materials Supplementary materials and methods. Figures S1–S6: cyclic voltammograms of compounds **1a–c** and **2a–c** (scan rate of 20 mV s^−1^). Figures S7–S8: HMBC and expanded HMBC (400/100 MHz, TCE-*d*_2_) of compound **3a**. Figures S9–S10: ORTEP diagrams of **2b** and **3b** with 50% thermal ellipsoids. The chloroform molecules are omitted for clarity. Figures S11–S16: TGA data for **2a–c** and **3a–c** under a N_2_ gas flow with temperature ramp rate of 10°C/min until 600°C. Figures S17–S55: NMR spectra of compounds **1b**, **1c**, **2a–c**, **IIa**, and **3a–c**. Figures S56-S63: UV–Vis spectra of compounds **1b**, **1c**, **2a–c**, and **3a–c**. Figure S64–S71: MALDI-TOF HRMS spectra of compounds **1b**, **1c**, **2a–c**, and **3a–**c. Table S1: optimization of the reaction conditions. Table S2: crystal data and structure refinement for compound **2b**. Table S3: crystal data and structure refinement for compound **3b**.
