# Supplementary material for: Successively Regioselective Electrosynthesis and Electron Transport Property of Stable Multiply Functionalized [60]Fullerene Derivatives
Source: Research (Wash D C). 2020 Feb 15;2020:2059190. doi: 10.34133/2020/2059190 (PMC7044465; doi:10.34133/2020/2059190)
Supplement: Supplementary Materials — and methods. Figures S1–S6: cyclic voltammograms of compounds 1a–c and 2a–c (scan rate of 20 mV s−1). Figures S7–S8: HMBC and expanded HMBC (400/100 MHz, TCE-d2) of compound 3a. Figures S9–S10: ORTEP diagrams of 2b and 3b with 50% thermal ellipsoids. The chloroform molecules are omitted for clarity. Figures S11–S16: TGA data for 2a–c and 3a–c under a N2 gas flow with temperature ramp rate of 10°C/min until 600°C. Figures S17–S55: NMR spectra of compounds 1b, 1c, 2a–c, IIa, and 3a–c. Figures S56-S63: UV–Vis spectra of compounds 1b, 1c, 2a–c, and 3a–c. Figure S64–S71: MALDI-TOF HRMS spectra of compounds 1b, 1c, 2a–c, and 3a–c. Table S1: optimization of the reaction conditions. Table S2: crystal data and structure refinement for compound 2b. Table S3: crystal data and structure refinement for compound 3b. [file 2059190.f1.pdf]

Supplementary Materials

Successively Regioselective Electrosynthesis and Electron Transport Property of Stable Multiply Functionalized [60]Fullerene Derivatives

Xing-Xing Yan,[1] Bairu Li,[2] Hao-Sheng Lin,[1] Fei Jin,[2] Chuang Niu,[1] Kai-Qing Liu,[1] Guan-Wu Wang,*[,1,3] and Shangfeng Yang*[,2]

[1]Hefei National Laboratory for Physical Sciences at Microscale, CAS Key Laboratory of Soft Matter Chemistry, Center for Excellence in Molecular Synthesis of CAS, and Department of Chemistry, University of Science and Technology of China, Hefei, Anhui 230026, P. R. China

E-mail: gwang@ustc.edu.cn

[2]Hefei National Laboratory for Physical Sciences at Microscale, CAS Key Laboratory of Materials for Energy Conversion, and Department of Materials Science and Engineering, University of Science and Technology of China, Hefei 230026, China

E-mail: sfyang@ustc.edu.cn

[3]State Key Laboratory of Applied Organic Chemistry, Lanzhou University, Lanzhou, Gansu 730000, China

Table of Contents

1. General Methods S2
2. Optimization of the Reaction Conditions S2
3. Experimental Procedures and Spectral Data for Products **1b**, **1c**, **2a**–**c**, **IIa**, and **3a**–**c** S3
4. Cyclic Voltammograms of Compounds **1a**–**c** and **2a**–**c** S9
5. HMBC Spectrum of Compound **3a** S12
6. Single-Crystal X-Ray Crystallography of Compounds **2b** and **3b** S13
7. TGA Analyses of Products **2a**–**c** and **3a**–**c** S17
8. NMR Spectra of Compounds **1b**, **1c**, **2a-c**, **IIa**, and **3a**–**c** S20
9. UV-vis Spectra of Compounds **1b**, **1c**, **2a**–**c**, and **3a**–**c** S40
10. MALDI-TOF HRMS Spectra of Compounds **1b**, **1c**, **2a-c**, and **3a-c** S44
11. References S52

1. General Methods

All electrochemical measurements and reactions were performed under an argon atmosphere at room temperature (~25 ^oC) using a SHANGHAI CHENHUA CHI600E workstation. Tetra-*n*-butylammonium perchlorate (TBAP) was recrystallized from absolute ethanol and dried in a vacuum at 313 K prior to use. Other chemicals were obtained commercially and used without further purification. Controlled potential electrolysis (CPE) was carried out on a potentiostat/galvanostat using an "H" type cell which consisting of two platinum gauze electrodes (serving as working and counter electrodes, respectively) separated by a sintered glass frit. A conventional three-electrode cell was used for CV measurements and consisted of a 2-mm diameter platinum disc working electrode, a platinum wire counter electrode, and a saturated calomel reference electrode (SCE). The SCE was separated from the bulk of the solution by a fritted-glass bridge of low porosity which contained the solvent/supporting electrolyte mixture.

2. Optimization of the Reaction Conditions.

Firstly, CPE of **1a** was carried out at –1.24 V under an argon atmosphere at room temperature in *ortho*-dichlorobenzene (ODCB) solution containing 0.1 M TBAP as the supporting electrolyte. The potentiostat was turned off after the theoretical coulombs was reached, indicating that the transformation process of **1a** to **1a**$^{2-}$ was fulfilled. Satisfactorily, the desired product **2a** was obtained in 30% yield when the reaction was performed by using 0.02 mmol of **1a**, 0.1 mmol of phthaloyl chloride, and 0.2 mmol of NaH in 25 mL of anhydrous ODCB containing 0.1 M TBAP at 0 ^oC for 2 h under an argon atmosphere (Table S1, entry 1). The addition of NaH was intended to remove the possibly contaminated HCl, phthalic acid and trace amount of water present in this reaction system.[1] The reaction time was further investigated, and the results indicated that shortening or prolonging the reaction time did not enhance the yield of product (Table S1, entries 2 and 3). It should be noted that an obviously increased yield (36%) was obtained when the amount of phthaloyl chloride was increased from 5 equiv to 10 equiv (Table S1, entry 1 *vs.* entry 4). When 20 equiv of phthaloyl chloride was introduced into this reaction, a higher yield of 38% was obtained (Table S1, entry 5). However, further increasing the dosage of phthaloyl chloride could not achieve a better yield (Table S1, entry 6). Next, the amount of NaH was also investigated, and the results indicated NaH was actually not necessary for this reaction, since a slightly higher yield was obtained in the absence of NaH (Table S1, entry 8 *vs.* entries 5 and 7). In addition, the reaction temperature was also varied in a bid to improve the yield, but it was found that 0 ^oC was still the best choice (Table S1, entry 8 *vs.* entries 9 and 10). Thus, the optimal reaction conditions were confirmed as follows: the molar ratio of **1a** and phthaloyl chloride was 1:20, and the reaction was carried out at 0 ^oC for 2 h in 25 mL anhydrous ODCB containing 0.1 M TBAP under an argon atmosphere (Table S1, entry 8).

Table S1: Optimization of the Reaction Conditions[a]

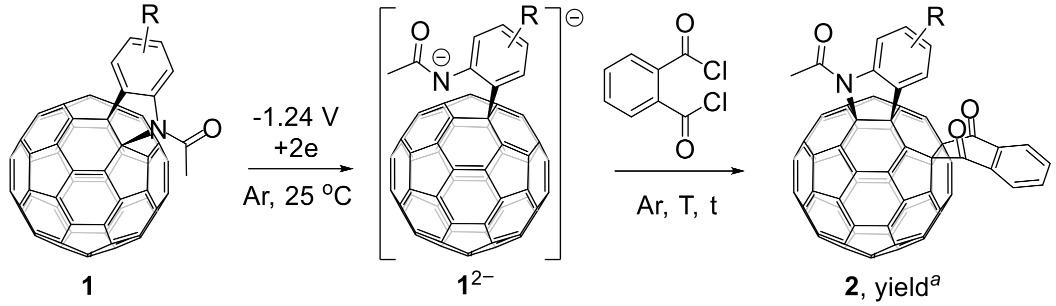

Entry	Phthaloyl chloride (equiv)	NaH (equiv)	Temp.(°C)	Time (h)	Yield (%)[b]
1	5	10	0	2	30
2	5	10	0	1	17
3	5	10	0	5	30
4	10	10	0	2	36
5	20	10	0	2	38
6	50	10	0	2	23
7	20	20	0	2	35
8	20	-	0	2	40
9	20	-	-10	2	37
10	20	-	25	2	27

[a]The reactions were carried out under an argon atmosphere using a SHANGHAI CHENHUA CHI600E workstation with 0.02 mmol of **1a** in anhydrous ODCB (25 mL). [b]Isolated yield based on **1a**.

3. Experimental Procedures and Spectral Data for Products 1b, 1c, 2a–c, IIa, and 3a–c

Synthesis of Compound 1b. By following our previous procedure,[2] to a solution of C_{60} (36.0 mg, 0.050 mmol) in a mixture of ODCB (4 mL) and CH_3CN (0.5 mL) was sequentially added *N*-(3-methoxyphenyl)acetamide (41.2 mg, 0.250 mmol), $Pd(OAc)_2$ (1.1 mg, 0.0050 mmol), *p*-toluenesulfonic acid monohydrate (*p*-TSA) (9.8 mg, 0.050 mmol), and $K_2S_2O_8$ (65.0 mg, 0.25 mmol). After being stirred at 130 °C for 24 h. The reaction was filtered through a silica gel (200-300 mesh) plug in order to remove any insoluble materials. After evaporation in vacuo, the residue was separated on a silica gel column (300-400 mesh) with CS_2/CH_2Cl_2 (4:1 v/v) as the eluent to give recovered C_{60} (9.4 mg, 26%) and then the desired product **1b** (15.3 mg, 35%) as an amorphous brown solid. ^1H NMR (400 MHz, $CS_2/CDCl_3$), δ 8.00 (d, J = 8.4 Hz, 1H), 7.29 (d, J = 2.2 Hz, 1H), 6.90 (dd, J = 8.4 Hz, 2.2 Hz, 1H), 3.99 (s, 3H), 2.89 (s, 3H); ^{13}C NMR (100 MHz, $CS_2/CDCl_3$, with $Cr(acac)_3$ as relaxation reagent, all 2C unless indicated) δ 168.70 (1C, *C*=O), 160.82 (1C, aryl *C*), 152.98, 147.90, 147.60 (1C), 147.11 (1C),

146.23 (4C), 146.04, 145.96, 145.68, 145.60, 145.08, 144.93, 144.87, 144.85, 144.64, 144.33 (4C), 142.76, 142.65, 142.59 (4C), 142.16, 141.91 (4C), 141.87, 141.58, 141.41, 140.83, 137.28, 136.53, 134.63 (1C, aryl *C*), 126.79 (1C, aryl *C*), 123.26 (1C, aryl *C*), 108.63 (1C, aryl *C*), 103.34 (1C, aryl *C*), 87.55 (1C, sp^3-*C* of C_{60}), 70.51 (1C, sp^3-*C* of C_{60}), 55.56 (1C), 27.72 (1C); UV-vis ($CHCl_3$) λ_{max} nm (log ε) 256.5 (4.91), 312.8 (4.43), 427.7 (3.28), 479.5 (3.02), 551.5 (2.78), 612.5 (2.48), 678.6 (2.18); FT-IR ν/cm^{-1} (KBr) 2921, 2851, 1672, 1593, 1498, 1429, 1363, 1337, 1219, 1034, 816, 785, 622, 595, 574, 548, 525; MALDI-TOF MS m/z calcd for $C_{69}H_9NO_2$ $[M]^-$ 883.0639, found 883.0638.

Synthesis of Compound 1c. By following our previous procedure,[2] to a solution of C_{60} (36.0 mg, 0.050 mmol) in a mixture of ODCB (4 mL) and CH_3CN (0.5 mL) was sequentially added *N*-(3,4-dimethoxyphenyl)acetamide (49.2 mg, 0.250 mmol), $Pd(OAc)_2$ (1.1 mg, 0.0050 mmol), *p*-TSA (9.5 mg, 0.050 mmol), and $K_2S_2O_8$ (68.0 mg, 0.25 mmol). After being stirred at 130 °C for 24 h. The reaction was filtered through a silica gel (200-300 mesh) plug in order to remove any insoluble materials. After evaporation in vacuo, the residue was separated on a silica gel column (300-400 mesh) with CS_2/CH_2Cl_2 (1:1 v/v) as the eluent to give recovered C_{60} (8.2 mg, 23%) and then the desired product **1c** (19.2 mg, 42%) as an amorphous brown solid. ^{1}H NMR (400 MHz, $CS_2/CDCl_3$), δ 7.54 (s, 1H), 7.40 (br, 1H), 4.09 (s, 3H), 3.96 (s, 3H), 2.91 (s, 3H); ^{13}C NMR (100 MHz, $CS_2/CDCl_3$, all 2C unless indicated) δ 168.25 (1C, *C*=O), 152.74, 150.28 (1C, aryl *C*), 147.86, 147.78 (1C), 147.29 (1C), 146.94, 146.42 (4C), 146.20, 146.12, 145.85, 145.76, 145.25, 145.12 (4C), 145.03, 144.67, 144.48 (4C), 142.95, 142.77 (6C), 142.30, 142.04 (4C), 141.75, 141.60, 141.03, 137.48, 136.69, 135.00 (1C, aryl *C*), 134.26 (1C, aryl *C*), 122.21 (1C, aryl *C*), 108.69 (1C, aryl *C*), 101.52 (1C, aryl *C*), 87.40 (1C, sp^3-*C* of C_{60}), 71.59 (1C, sp^3-*C* of C_{60}), 56.46 (1C), 56.31 (1C), 27.77 (1C); UV-vis ($CHCl_3$) λ_{max} nm (log ε) 255.9 (5.08), 314.5 (4.67), 427.9 (3.45), 471.0 (3.26), 547.5 (3.02), 608.5 (2.74), 679.7 (2.40); FT-IR ν/cm^{-1} (KBr) 2920, 2848, 1667, 1603, 1501, 1460, 1433, 1371, 1291, 1218, 1145, 1037, 816, 763, 559, 526; MALDI-TOF MS *m/z* calcd for $C_{70}H_{11}NO_3$ $[M]^-$ 913.0744, found 913.0746.

Synthesis of Compound 2a. The dianionic $\mathbf{1a}^{2-}$ was obtained by electroreduction from [60]fulleroindoline **1a** (17.3 mg, 0.020 mmol) at –1.24 V by CPE, and then reacted with phthaloyl chloride (58.0 μL, 0.400 mmol). After being stirred at 0 °C for 2 h, the resulting mixture was directly filtered through a silica gel (200-300 mesh) plug with CS_2/CH_2Cl_2 (1:1 v/v) to remove the supporting electrolyte and insoluble materials, and then evaporated in vacuo to remove the solvent. Next, the residue was further separated on a silica gel column (300–400 mesh) with CS_2/CH_2Cl_2 (10:1 v/v) as the eluent to afford **2a** (7.9 mg, 40%) as an amorphous brown solid along with unreacted **1a** (1.0 mg, 6%). ^{1}H NMR (400 MHz, TCE-d_2), δ 8.40-8.30 (m, 2H), 8.14-8.06 (m, 2H), 7.33 (s, 1H), 7.11 (d, *J* = 7.7 Hz, 1H), 6.96 (d, *J* = 7.7 Hz, 1H), 2.68 (s, 3H), 2.44 (s, 3H); ^{13}C NMR (100 MHz, TCE-d_2, all 1C unless indicated) δ 191.08 (*C*=O), 189.91 (*C*=O),

169.56 (C=O), 162.48, 156.00, 152.85, 151.45, 150.91, 150.72, 149.06, 149.00, 148.96, 148.81, 148.32, 148.13, 147.75, 147.52, 147.40 (2C), 147.21, 147.18, 147.12, 146.76, 146.12 (2C), 145.96, 145.74, 145.73, 145.66, 145.25, 145.05, 144.93 (2C), 144.87, 144.80, 144.36, 144.13, 143.90 (3C), 143.88, 143.39, 142.86, 142.82, 142.57, 141.95, 141.71, 141.55, 140.94, 140.59 (2C), 140.58, 140.33, 140.13, 140.05, 139.36 (aryl C), 138.40, 137.83, 137.62, 136.44 (aryl C), 136.41 (aryl C), 135 32, 135.08, 134.57 (aryl C), 134.38 (aryl C), 129.94 (aryl C), 128.91 (aryl C), 128.75 (aryl C), 125.60 (aryl C), 124.98 (aryl C), 116.68 (aryl C), 80.31 (sp^3-C of C_{60}), 73.21 (sp^3-C of C_{60}), 72.18 (sp^3-C of C_{60}), 64.72 (sp^3-C of C_{60}), 27.56, 22.49; UV-vis ($CHCl_3$) λ_{max} nm (log ε) 238.0 (5.04), 254.9 (5.01), 300.5 (4.66), 331.3 (4.51), 497.0 (3.50), 583.3 (3.02), 721.2 (3.04); FT-IR ν/cm^{-1} (KBr) 2915, 2845, 1689, 1591, 1498, 1428, 1367, 1340, 1250, 1044, 994, 792, 759, 745, 721, 690, 546, 526; MALDI-TOF MS *m/z* calcd for $C_{77}H_{13}NO_3$ $[M]^-$ 999.0901, found 999.0902.

Synthesis of Compound 2b. The dianionic **1b**$^{2-}$ was obtained by electroreduction from [60]fulleroindoline **1b** (17.7 mg, 0.020 mmol) at –1.24 V by CPE, and then reacted with phthaloyl chloride (58.0 μL, 0.400 mmol). After being stirred at 0 °C for 5 h, the resulting mixture was directly filtered through a silica gel (200-300 mesh) plug with CS_2/CH_2Cl_2 (1:1 v/v) to remove the supporting electrolyte and insoluble materials, and then evaporated in vacuo to remove the solvent. Next, the residue was further separated on a silica gel column (300–400 mesh) with CS_2/CH_2Cl_2 (4:1 v/v) as the eluent to afford **2b** (8.3 mg, 41%) as an amorphous brown solid along with unreacted **1b** (1.5 mg, 8%). ^{1}H NMR (400 MHz, $CS_2/CDCl_3$), δ 8.30-8.26 (m, 1H), 8.26-8.22 (m, 1H), 8.05-8.00 (m, 2H), 7.02 (d, J = 8.4 Hz, 1H), 7.06-6.95 (br, 1H), 6.52 (dd, J = 8.4 Hz, 2.2 Hz, 1H), 3.81 (s, 3H), 2.62 (s, 3H); ^{13}C NMR (100 MHz, $CS_2/CDCl_3$, all 1C unless indicated) δ 190.32 (C=O), 188.94 (C=O), 168.47 (C=O), 162.32, 160.69 (aryl C), 155.50, 152.46, 151.21, 150.75, 150.48, 148.85, 148.82, 148.70, 148.33, 148.13, 148.02, 147.58, 147.36, 147.21, 147.09, 147.05, 146.92, 146.84, 146.49, 145.880, 145.878, 145.75, 145.62, 145.50, 145.37, 144.97, 144.82, 144.73, 144.70 (2C), 144.63, 144.08, 143.88, 143.81 (2C), 143.72, 143.18, 142.72 (2C), 142.38, 141.81, 141.49, 141.34, 141.22, 140.64, 140.14, 140.07, 139.83 (2C), 139.07, 138.31, 137.72, 137.38, 135.59 (2C, aryl C), 135.48, 135.24, 134.29 (aryl C), 134.06 (aryl C), 129.01 (aryl C), 128.47 (aryl C), 128.29 (aryl C), 128.24 (aryl C), 125.67 (aryl C), 108.38 (aryl C), 103.17 (aryl C), 80.60 (sp^3-C of C_{60}), 73.06 (sp^3-C of C_{60}), 71.96 (sp^3-C of C_{60}), 60.04 (sp^3-C of C_{60}), 55.59, 26.93; UV-vis ($CHCl_3$) λ_{max} nm (log ε) 255.0 (5.09), 304.5 (4.79), 365.5 (4.43), 490.5 (3.76), 645.5 (2.95), 678.5 (2.74); FT-IR ν/cm^{-1} (KBr) 2919, 2846, 1676, 1608, 1592, 1497, 1432, 1367, 1342, 1309, 1250, 1216, 1174, 1043, 997, 817, 790, 744, 721, 689, 624, 546, 524; MALDI-TOF MS *m/z* calcd for $C_{77}H_{13}NO_4$ $[M]^+$ 1015.0839, found 1015.0826.

Synthesis of Compound 2c. The dianionic **1c**$^{2-}$ was obtained by electroreduction from [60]fulleroindoline **1c** (18.2 mg, 0.020 mmol) at –1.24 V by CPE, and then reacted with phthaloyl chloride (29.0 μL, 0.200 mmol). After being stirred at 0 °C for 2 h, the resulting mixture was directly filtered through a silica gel (200-300 mesh) plug with CS_2/CH_2Cl_2 (1:1 v/v) to remove the supporting electrolyte and insoluble materials, and then evaporated in vacuo to remove the solvent. Next, the residue was further separated on a silica gel column (300–400 mesh) with CS_2/CH_2Cl_2 (1:1 v/v) as the eluent to afford **2c** (10.0 mg, 48%) as an amorphous brown solid along with unreacted **1c** (0.5 mg, 3%). ^{1}H NMR (400 MHz, $CS_2/CDCl_3$), δ 8.43-8.37 (m, 2H), 8.12-8.04 (m, 2H), 7.12-6.93 (brs, 1H), 6.66 (s, 1H), 3.94 (s, 3H), 3.22 (s, 3H), 2.67 (s, 3H); ^{13}C NMR (100 MHz, CS_2/TCE-d_2, with $Cr(acac)_3$ as relaxation reagent, all 1C unless indicated) δ 190.30 (*C*=O), 189.09 (*C*=O), 168.04 (*C*=O), 162.56, 155.98, 152.75, 151.28, 150.98, 150.53, 149.86 (aryl *C*), 148.92, 148.82 (2C), 148.76, 148.18, 147.97, 147.64, 147.29, 147.24, 147.19, 147.08, 147.02, 146.95, 146.67, 146.33 (2C), 146.07, 145.95, 145.61, 145.43, 145.08, 144.88, 144.83 (3C), 144.68, 144.10, 143.98, 143.78 (3C), 143.22, 142.78, 142.38, 141.86, 141.50 (2C), 141.44, 140.72 (2C), 140.35, 140.18 (2C), 139.89, 139.21, 138.20, 137.94, 137.40, 136.52 (aryl *C*), 136.49 (aryl *C*), 134.72, 134.50 (aryl *C*), 134.40 (aryl *C*), 134.15, 129.24 (aryl *C*), 128.92 (aryl *C*), 128.79 (aryl *C*), 128.43 (aryl *C*), 125.53 (aryl *C*), 107.43 (aryl *C*), 100.87 (aryl *C*), 80.39 (sp^3-*C* of C_{60}), 72.58 (sp^3-*C* of C_{60}), 71.44 (sp^3-*C* of C_{60}), 64.20 (sp^3-*C* of C_{60}), 56.61, 54.87, 26.92; UV-vis ($CHCl_3$) λ_{max} nm (log ε) 255.5 (4.58), 324.0 (4.36), 364.5 (4.08), 490.9 (3.45), 640.0 (2.74), 687.0 (2.60); FT-IR ν/cm^{-1} (KBr) 2922, 2849, 1671, 1589, 1501, 1434, 1372, 1292, 1250, 1212, 1147, 1089, 1044, 993, 914, 822, 774, 744, 686, 654, 546, 522; MALDI-TOF MS *m/z* calcd for $C_{78}H_{15}NO_5$ $[M]^+$ 1045.0945, found 1045.0933.

Synthesis of Compound IIa. Compound **2a** (10.1 mg, 0.01 mmol) was dissolved in ODCB containing 0.1 M TBAP, and then by CPE at –1.20 V. The potentiostat was turned off after the theoretical coulombs was reached, and TFA (1.5 μL, 0.02 mmol) was added and stirred for 5 min. The resulting mixture was directly filtered through a silica gel (200-300 mesh) plug with CS_2/CH_2Cl_2 (1:1 v/v) to remove the supporting electrolyte and insoluble materials, and then evaporated in vacuo to remove the solvent. Next, the residue was further separated on a silica gel column (300–400 mesh) with CS_2/CH_2Cl_2 (10:1 v/v) as the eluent to afford **IIa**[3] (3.5 mg, 40%) as an amorphous brown solid. ^{1}H NMR (400 MHz, $CS_2/CDCl_3$), δ 7.99 (d, J = 7.7 Hz, 1H), 7.43 (s, 1H), 7.30 (d, J = 7.7 Hz, 1H), 6.37 (d, J = 1.7 Hz, 1H), 6.35 (d, J = 1.7 Hz, 1H), 2.90 (s, 3H), 2.62 (s, 3H).

Synthesis of Compound 3a. The mixture of **2a** (10.0 mg, 0.01 mmol) and trifluoroacetic acid (TFA) (0.74 μL, 0.01 mmol) was dissolved in ODCB containing 0.1 M TBAP, and then by CPE at –1.20 V. The potentiostat was turned off after the theoretical coulombs was reached. The resulting mixture was directly filtered through

a silica gel (200-300 mesh) plug with CS_2/CH_2Cl_2 (1:1 v/v) to remove the supporting electrolyte and insoluble materials, and then evaporated in vacuo to remove the solvent. Next, the residue was further separated on a silica gel column (300–400 mesh) with CS_2/CH_2Cl_2 (10:1 v/v) as the eluent to afford **3a** (4.0 mg, 40%) as an amorphous red-brown solid along with a minor byproduct **IIa** (0.3 mg). ^{1}H NMR (400 MHz, TCE-d_2), δ 8.32-8.27 (m, 1H), 8.27-8.21 (m, 1H), 8.06-7.99 (m, 2H), 7.35 (s, 1H), 7.13 (d, J = 7.7 Hz, 1H), 7.08 (d, J = 7.7 Hz, 1H), 6.09 (d, J = 2.3 Hz, 1H), 6.02 (d, J = 2.3 Hz, 1H), 2.82 (s, 3H), 2.49 (s, 3H); ^{13}C NMR (100 MHz, TCE-d_2, all 1C unless indicated) δ 193.80 (*C*=O), 191.99 (*C*=O), 169.32 (*C*=O), 153.71, 149.24, 148.92, 148.84 (2C), 148.59, 148.55, 148.41, 148.32 (2C), 148.15, 147.89 (2C), 147.69, 147.65, 147.56, 147.50, 147.15, 147.09, 146.94, 146.88, 146.78, 146.00, 145.64, 145.60, 145.57, 145.47, 145.17, 145.05 (2C), 144.95, 144.88, 144.73, 144.44, 143.34, 143.95, 143.50, 143.44, 142.95, 142.80, 142.71, 141.89, 141.51, 141.50, 141.33, 141.17, 140.88, 140.80, 140.42, 140.18, 140.10, 139.51 (aryl *C*), 138.77, 137.58, 136.03, 136.03 (aryl *C*), 135.64 (aryl *C*), 135.57 (aryl *C*), 135.49 (aryl *C*), 134.76 (aryl *C*), 134.18 (aryl *C*), 129.25 (aryl *C*), 127.73 (aryl *C*), 126.59 (aryl *C*), 124.33 (aryl *C*), 115.79 (aryl *C*), 80.56 (sp^3-*C* of C_{60}), 71.03 (sp^3-*C* of C_{60}), 68.32 (sp^3-*C* of C_{60}), 61.02 (sp^3-*C* of C_{60}), 57.04 (sp^3-*C* of C_{60}), 56.03 (sp^3-*C* of C_{60}), 28.15, 22.48; UV-vis ($CHCl_3$) λ_{max} nm (log ε) 236.8 (5.00), 253.8 (5.00), 311.0 (4.54), 336.8 (4.42), 387.3 (4.06), 427.4 (3.79), 522.8 (3.13), 619.2 (2.54), 675.5 (2.18); FT-IR ν/cm^{-1} (KBr) 2918, 2845, 1673, 1592, 1500, 1456, 1439, 1368, 1340, 1300, 1254, 1045, 999, 756, 745, 720, 597, 527, 519; MALDI-TOF MS *m/z* calcd for $C_{77}H_{15}NO_3$ [M]$^-$ 1001.1057, found 1001.1059.

Synthesis of Compound 3b. The mixture of **2b** (10.1 mg, 0.01 mmol) and TFA (0.74 μL, 0.01 mmol) was dissolved in ODCB containing 0.1 M TBAP, and then by CPE at –1.20 V. The potentiostat was turned off after the theoretical coulombs was reached. The resulting mixture was directly filtered through a silica gel (200-300 mesh) plug with CS_2/CH_2Cl_2 (1:1 v/v) to remove the supporting electrolyte and insoluble materials, and then evaporated in vacuo to remove the solvent. Next, the residue was further separated on a silica gel column (300–400 mesh) with CS_2/CH_2Cl_2 (4:1 v/v) as the eluent to afford **3b** (3.4 mg, 33%) as an amorphous red-brown solid along with 1,2,3,4-adduct **IIb** (0.3 mg). ^{1}H NMR (400 MHz, $CS_2/CDCl_3$), δ 8.31-8.26 (m, 1H), 8.25-8.20 (m, 1H), 8.03-7.96 (m, 2H), 7.18 (d, J = 8.4 Hz, 1H), 7.07 (brs, 1H), 6.77 (dd, J = 8.4 Hz, 2.1 Hz, 1H), 6.13 (d, J = 2.3 Hz, 1H), 6.04 (d, J = 2.3 Hz, 1H); ^{13}C NMR (100 MHz,TCE-d_2, all 1C unless indicated) δ 193.97 (*C*=O), 191.98 (*C*=O), 169.31 (*C*=O), 160.84 (aryl *C*), 153.70, 149.24, 148.92, 148.85 (2C), 148.59, 148.55, 148.41, 148.31 (2C), 148.16, 147.90 (2C), 147.69, 147.65, 147.60, 147.49, 147.13, 147.09, 146.91, 146.88, 146.78, 146.01, 145.60 (2C), 145.56, 145.45, 145.20, 145.05 (2C), 144.95, 144.84, 144.73, 144.44, 144.33, 143.85, 143.46, 143.28, 142.93, 142.90, 142.62, 141.91, 141.50 (2C), 141.34, 141.15, 140.89, 140.84, 140.45, 140.39, 140.18, 138.64, 137.54, 136.11, 136.02 (aryl *C*), 135.64 (aryl *C*), 135.61 (aryl *C*), 135.51 (aryl *C*),

134.20 (aryl *C*), 130.08 (aryl *C*), 129.24 (aryl *C*), 127.71 (aryl *C*), 125.17 (aryl *C*), 109.88 (aryl *C*), 102.88 (aryl *C*), 81.05 (sp^3-*C* of C_{60}), 71.01 (sp^3-*C* of C_{60}), 68.39 (sp^3-*C* of C_{60}), 60.70 (sp^3-*C* of C_{60}), 57.10 (sp^3-*C* of C_{60}), 56.30, 56.07 (sp^3-*C* of C_{60}), 28.02; UV-vis ($CHCl_3$) λ_{max} nm (log ε) 254.5 (4.75), 306.6 (4.32), 335.6 (4.16), 386.6 (3.83), 425.2 (3.56), 520.6 (2.90), 619.9 (2.30) ; FT-IR ν/cm^{-1} (KBr) 2921, 2851, 1682, 1609, 1594, 1498, 1433, 1370, 1343, 1311, 1256, 1220, 1173, 1094, 1045, 801, 754, 720, 528, 518; MALDI-TOF MS *m/z* calcd for $C_{77}H_{15}NO_4$ $[M]^-$ 1017.1007, found 1017.1025.

Synthesis of Compound 3c. The mixture of **2c** (10.5 mg, 0.01 mmol) and TFA (0.74 µL, 0.01 mmol) was dissolved in ODCB containing 0.1 M TBAP, and then by CPE at –1.13 V. The potentiostat was turned off after the theoretical coulombs was reached. And the resulting mixture was directly filtered through a silica gel (200-300 mesh) plug with CS_2/CH_2Cl_2 (1:1 v/v) to remove the supporting electrolyte and insoluble materials, and then evaporated in vacuo to remove the solvent. Next, the residue was further separated on a silica gel column (400-500 mesh) with CS_2/CH_2Cl_2 (1:1 v/v) as the eluent to afford **3c** (3.4 mg, 32%) as an amorphous red-brown solid along with 1,2,3,4-adduct **IIc** (0.3 mg). ^{1}H NMR (400 MHz, $CS_2/CDCl_3$), δ 8.27-8.21 (m, 1H), 8.19-8.13 (m, 1H), 8.00-7.93 (m, 2H), 7.01 (brs, 1H), 6.63 (s, 1H), 6.02 (d, *J* = 2.3 Hz, 1H), 6.00 (d, *J* = 2.3 Hz, 1H), 3.95 (s, 3H), 3.48 (s, 3H), 2.77 (s, 3H); ^{13}C NMR (100 MHz, TCE-d_2, all 1C unless indicated) δ 194.06 (*C*=O), 192.78 (*C*=O), 168.26 (*C*=O), 153.72, 149.97 (aryl *C*), 149.26, 148.96, 148.85 (2C), 148.60, 148.54, 148.43, 148.30 (2C), 148.17, 147.91 (2C), 147.64 (2C), 147.57, 147.40, 147.15, 147.11 (2C), 146.89, 146.72, 146.04, 145.64, 145.58, 145.52, 145.42, 145.20 (2C), 145.08, 145.03, 145.00, 144.81, 144.47, 144.35, 143.93, 143.37, 143.31, 143.08, 142.85, 142.53, 141.89, 141.51 (2C), 141.35, 141.25, 140.92, 140.83, 140.31 (2C), 140.18, 138.55, 137.54, 136.28, 135.88 (aryl *C*), 135.52 (aryl *C*), 135.46 (aryl *C*), 135.09 (aryl *C*), 134.40 (aryl *C*), 132.32 (aryl *C*), 129.25 (aryl *C*), 128.72 (aryl *C*), 127.78 (aryl *C*), 106.61 (aryl *C*), 100.35 (aryl *C*), 80.84 (sp^3-*C* of C_{60}), 70.69 (sp^3-*C* of C_{60}), 68.08 (sp^3-*C* of C_{60}), 61.00 (sp^3-*C* of C_{60}), 57.36 (sp^3-*C* of C_{60}), 56.94, 55.99 (sp^3-*C* of C_{60}), 55.82, 27.56; UV-vis ($CHCl_3$) λ_{max} nm (log ε) 255.5 (4.90), 311.2 (4.48), 340.3 (4.30), 388.6 (3.93), 424.8 (3.69),522.6 (3.08), 616.0 (2.48); FT-IR ν/cm^{-1} (KBr) 2924, 2853, 1687, 1665, 1593, 1512, 1461, 1434, 1376, 1291, 1255, 1218, 1093, 1045, 999, 819, 753, 720, 660, 605, 563, 527; MALDI-TOF MS *m/z* calcd for $C_{78}H_{17}NO_5$ $[M]^+$ 1047.1101, found 1047.1105.

4. Cyclic Voltammograms of Compounds 1a–c and 2a–c.

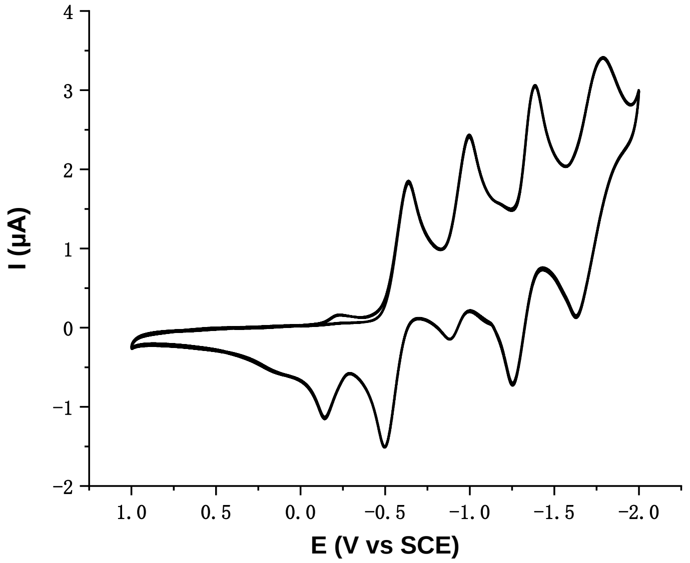

Figure S1 The cyclic voltammogram of compound 1a (scan rate of 20 mV s^{-1})

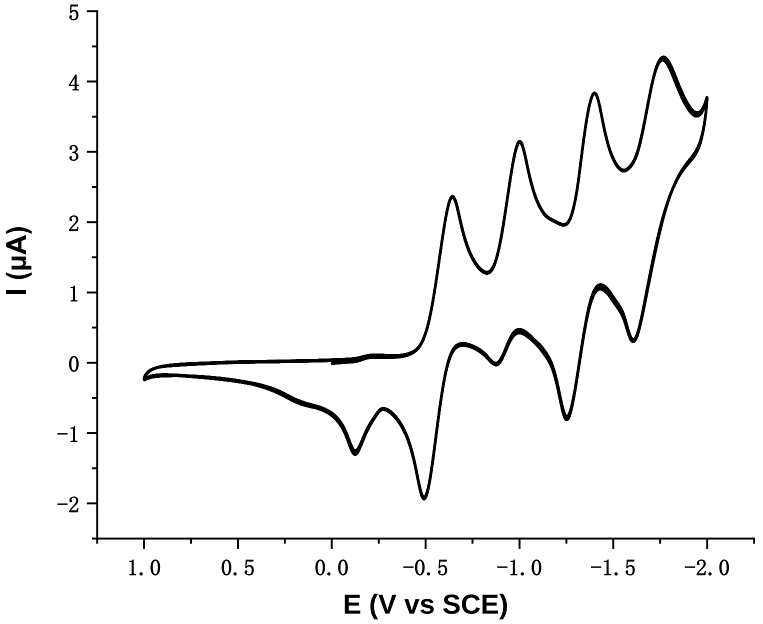

Figure S2 The cyclic voltammogram of compound 1b (scan rate of 20 mV s^{-1})

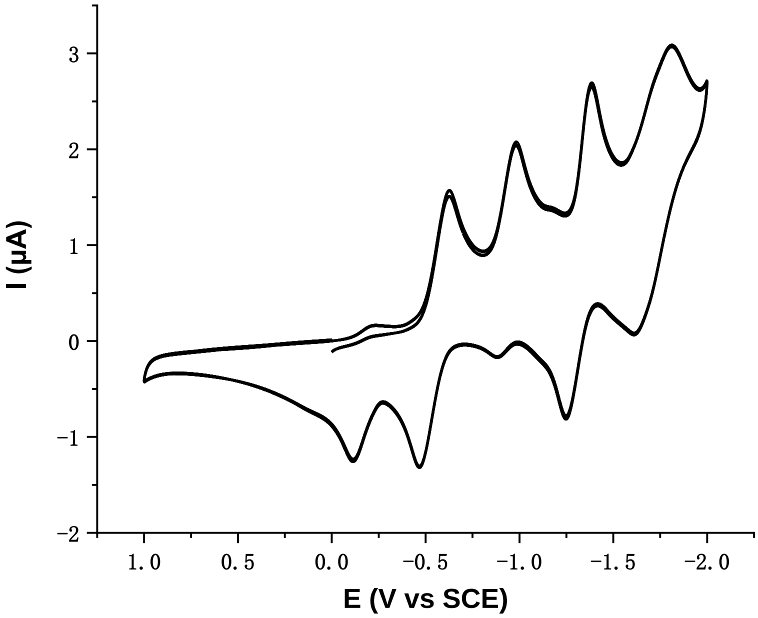

Figure S3 The cyclic voltammogram of compound 1c (scan rate of 20 mV s^{-1})

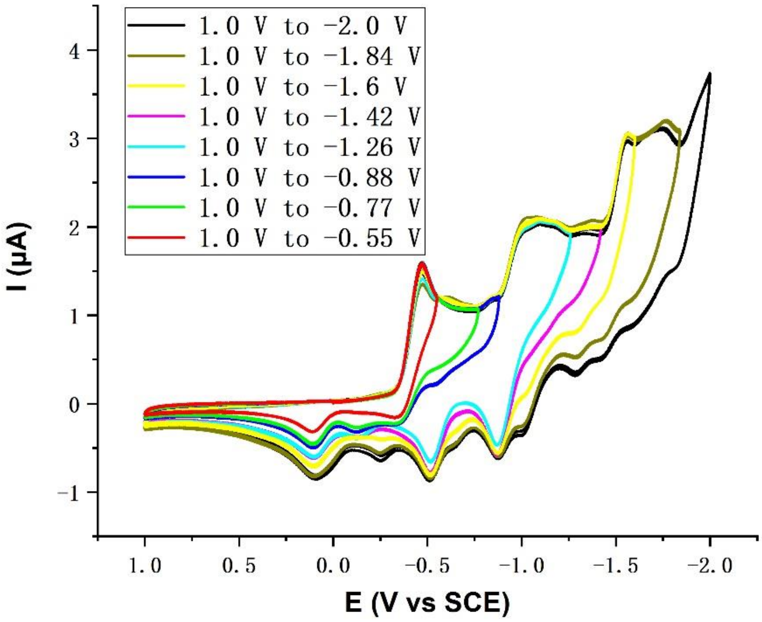

Figure S4. The cyclic voltammogram of compound 2a (scan rate of 20 mV s^{-1})

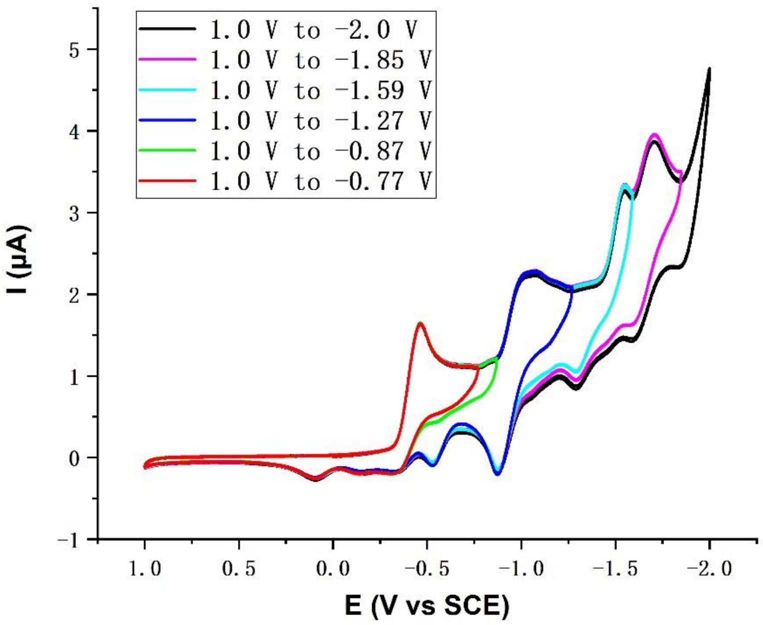

Figure S5. The cyclic voltammogram of compound 2b (scan rate of 20 mV s^{-1})

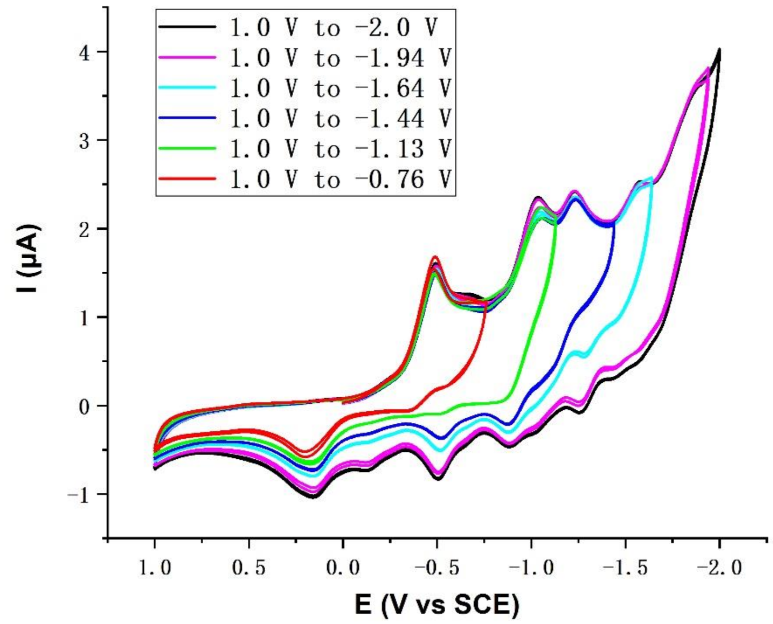

Figure S6. The cyclic voltammogram of compound 2c (scan rate of 20 mV s^{-1})

5. HMBC Spectrum of Compound 3a

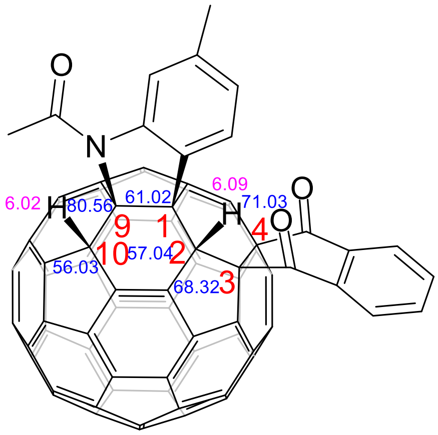

3a

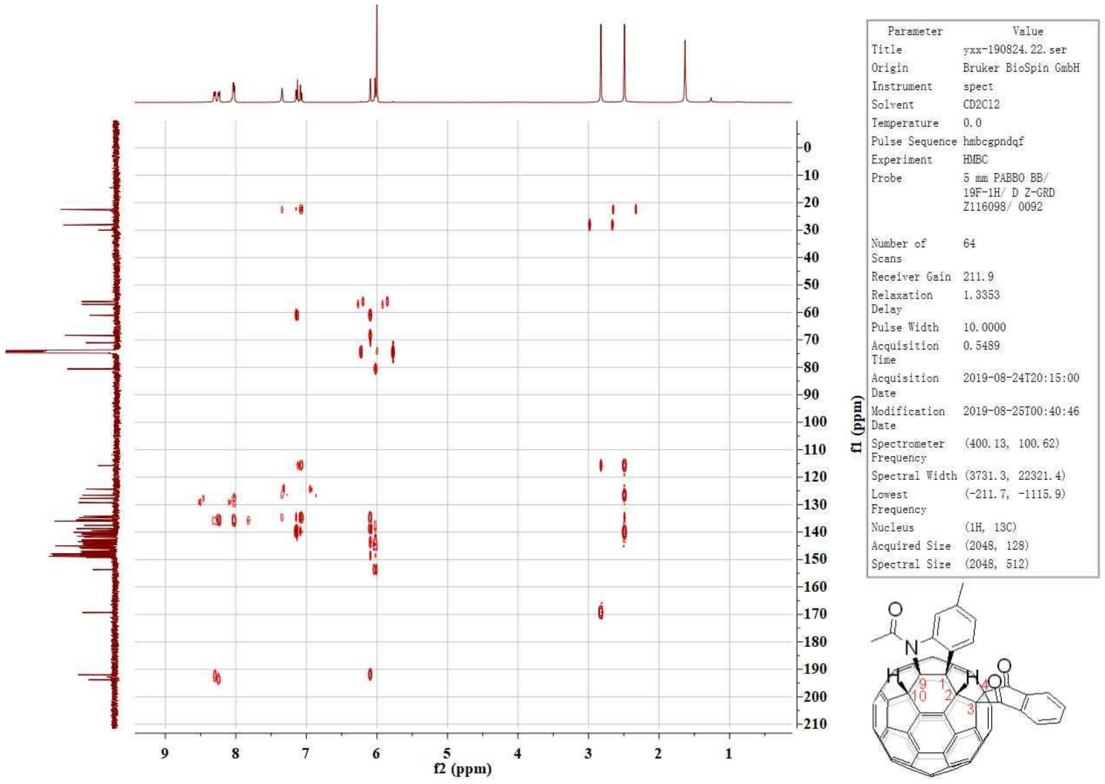

Figure S7 HMBC (400/100 MHz, TCE-d_2) of compound 3a

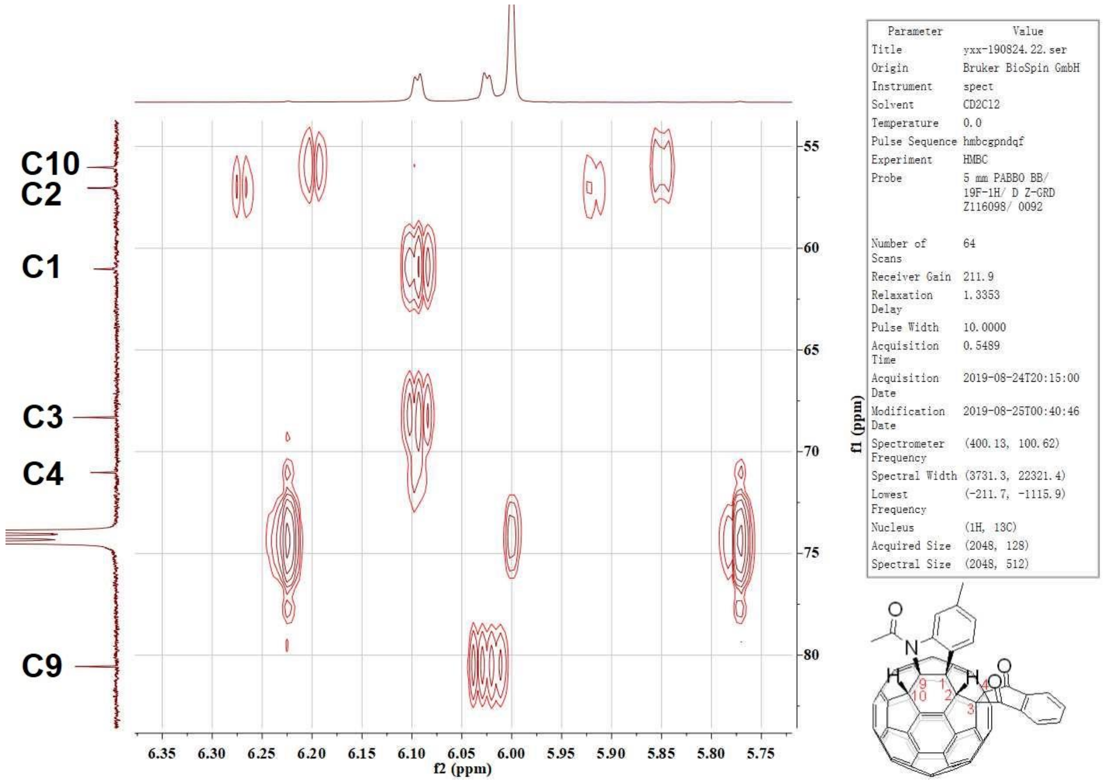

Figure S8 Expanded HMBC (400/100 MHz, TCE-d_2) of compound 3a

6. Single-Crystal X-Ray Crystallography of Compounds 2b and 3b

Single-Crystal X-Ray Crystallography of Compound 2b

Brown block crystals of **2b** were obtained by slow diffusion of methanol into a $CHCl_3$ solution of **2b** at 4 ^oC in a refrigerator. Single-crystal X-ray diffraction data were collected on a diffractometer equipped with a CCD area detector using graphite monochromated Cu Kα radiation (λ= 1.54184 Å) in the scan range 9.140°< 2θ < 142.904°. Using Olex2, the structure was solved with the ShelXS structure solution program using Direct Methods and refined with the ShelXL refinement package using Least Squares minimisation. Crystallographic data have been deposited in the Cambridge Crystallographic Data Centre as deposition number CCDC 1875965.

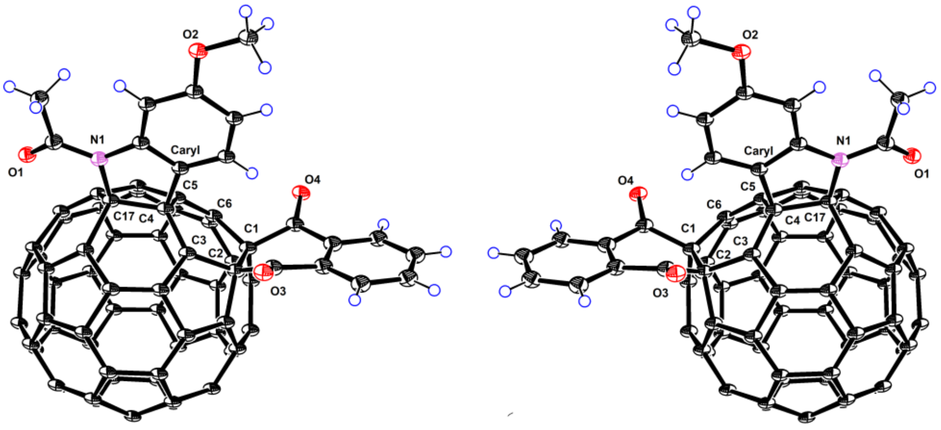

^{f}A-1,2,4,17-adduct ^{f}C-1,2,4,17-adduct

Figure S9. ORTEP diagrams of 2b with 50% thermal ellipsoids. The chloroform molecule is omitted for clarity.

Table S2. Crystal Data and Structure Refinement for Compound 2b

Identification code	1875965
Empirical formula	$C_{79}H_{15}Cl_6NO_4$
Formula weight	1254.62
Temperature/K	99.99(10)
Crystal system	orthorhombic
Space group	Pnma
a/Å	25.921(2)
b/Å	13.0956(8)
c/Å	14.3558(9)
α/°	90
β/°	90
γ/°	90
Volume/Å^3	4873.1(6)
Z	4
ρ_{calc} g/cm^3	1.710
μ/mm^{-1}	3.771
F(000)	2520.0
Crystal size/mm^3	0.280 × 0.250 × 0.120
Radiation	CuKα (λ = 1.54184)
2Θ range for data collection/°	9.140 to 142.904
Index ranges	$-19 \le h \le 31$, $-15 \le k \le 4$, $-11 \le l \le 17$
Reflections collected	9458
Independent reflections	4720 [R_{int} = 0.0975, R_{sigma} = 0.1144]
Data/restraints/parameters	4720/854/780
Goodness-of-fit on F^2	1.555
Final R indexes [I>=2σ (I)]	R_1 = 0.1467, wR_2 = 0.3751
Final R indexes [all data]	R_1 = 0.1777, wR_2 = 0.4293
Largest diff. peak/hole / e Å^{-3}	1.23/-1.03

Single-Crystal X-Ray Crystallography of Compound 3b

Brown block crystals of **3b** were obtained by slow evaporation of $CHCl_3$ solution of **3b** at 4 ^{o}C in a refrigerator. Single-crystal X-ray diffraction data were collected on a diffractometer equipped with a CCD area detector using graphite monochromated Cu Kα radiation (λ= 1.54184 Å) in the scan range 7.004°< 2θ < 142.962°. Using Olex2, the structure was solved with the ShelXS structure solution program using Direct Methods and refined with the ShelXL refinement package using Least Squares minimisation. Crystallographic data have been deposited in the Cambridge Crystallographic Data Centre as deposition number CCDC 1875973.

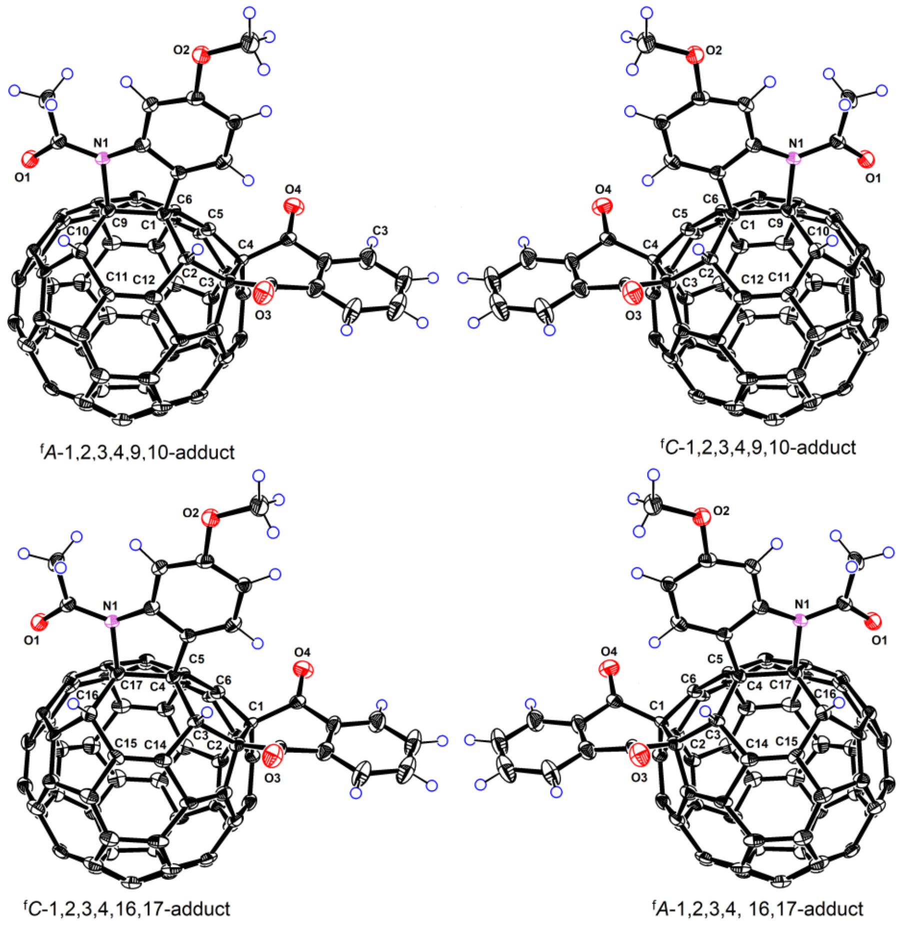

Figure S10. ORTEP diagrams of 3b with 50% thermal ellipsoids. The chloroform molecule is omitted for clarity.

Table S3. Crystal Data and Structure Refinement for Compound 3b

Identification code	1875973
Empirical formula	$C_{79}H_{17}Cl_6NO_4$
Formula weight	1256.64
Temperature/K	100.0(2)
Crystal system	orthorhombic
Space group	Pnma
a/Å	26.0038(5)
b/Å	13.1868(3)
c/Å	14.4351(3)
α/°	90
β/°	90
γ/°	90
Volume/Å^3	4949.90(18)
Z	4
ρ_{calc} g/cm^3	1.686
μ/mm^{-1}	3.713
F(000)	2528.0
Crystal size/mm^3	0.250 × 0.220 × 0.200
Radiation	CuKα (λ = 1.54184)
2Θ range for data collection/°	7.004 to 142.962
Index ranges	$-31 \le h \le 23$, $-13 \le k \le 15$, $-10 \le l \le 17$
Reflections collected	11318
Independent reflections	4825 [R_{int} = 0.0313, R_{sigma} = 0.0330]
Data/restraints/parameters	4825/372/765
Goodness-of-fit on F^2	1.092
Final R indexes [I>=2σ (I)]	R_1 = 0.0882, wR_2 = 0.2188
Final R indexes [all data]	R_1 = 0.0951, wR_2 = 0.2242
Largest diff. peak/hole / e Å^{-3}	1.08/-0.96

It should be noted that the disorder of fullerenes and its derivatives are very common. Some essential strains such as SADI, SIMU were employed to refine the above two crystals.[4,5]

7. TGA Analyses of Products 2a–c and 3a–c

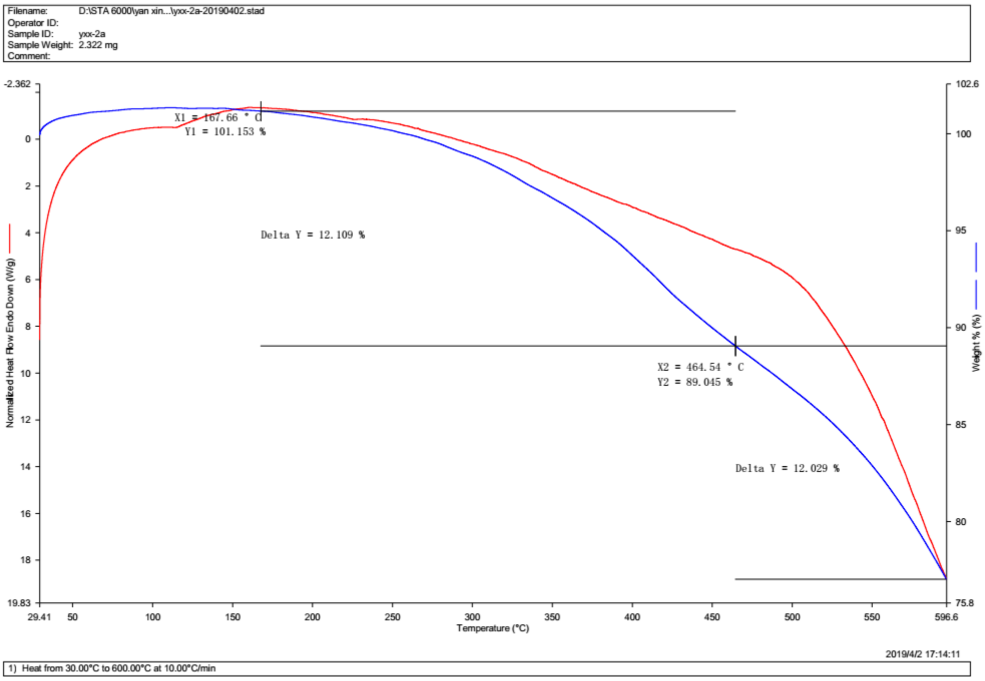

Figure S11. TGA data for 2a under a N_2 gas flow with temperature ramp rate of 10 ^oC/min until 600 ^oC.

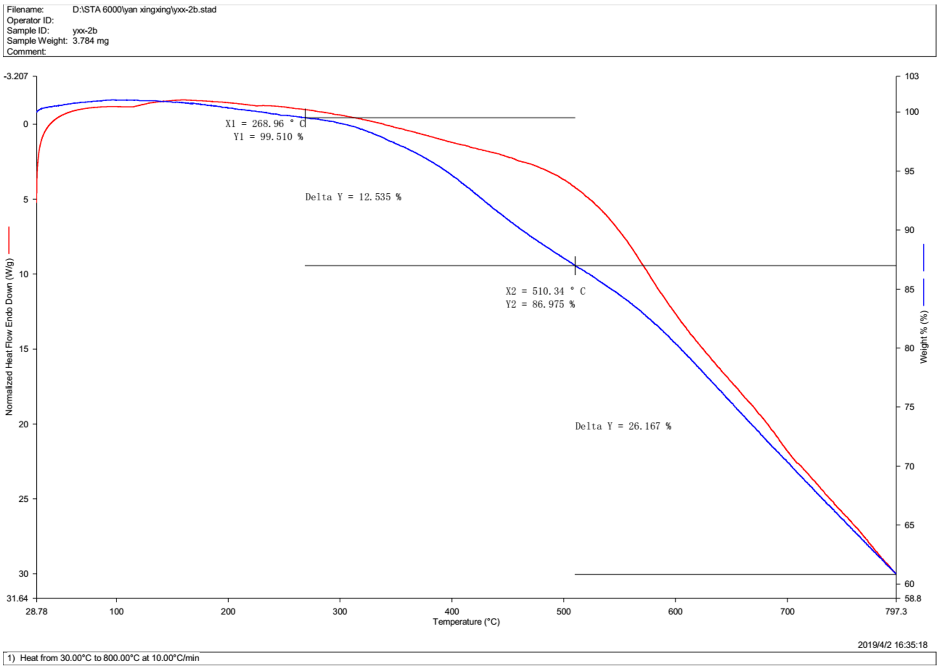

Figure S12. TGA data for 2b under a N_2 gas flow with temperature ramp rate of 10 ^oC/min until 800 ^oC.

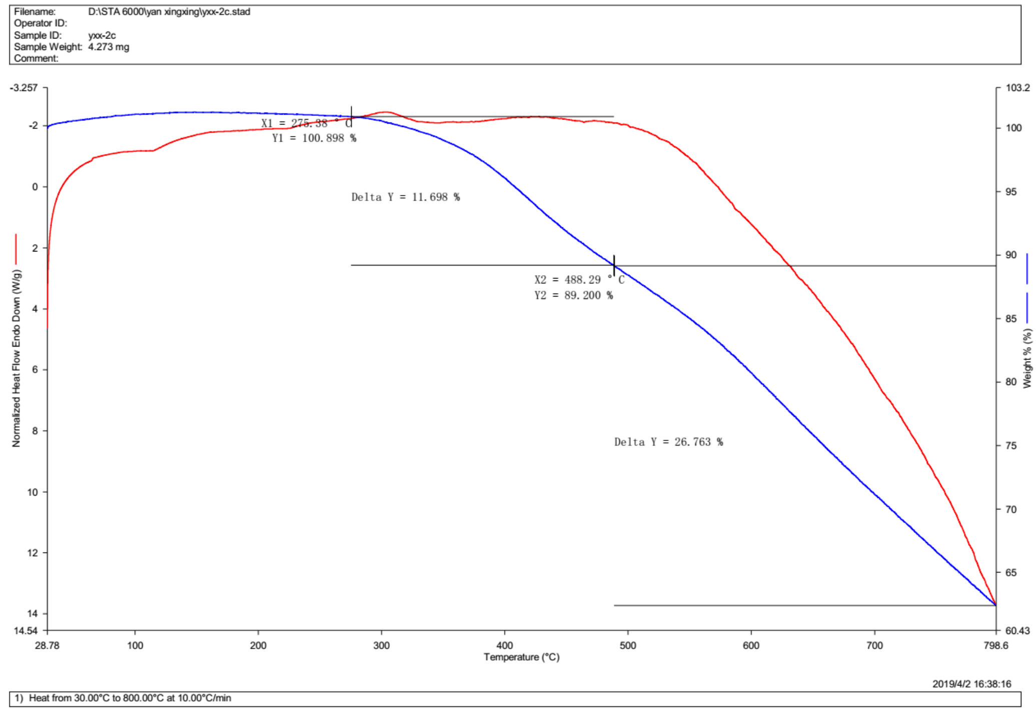

Figure S13. TGA data for 2c under a N_2 gas flow with temperature ramp rate of 10 ^oC/min until 800 ^oC.

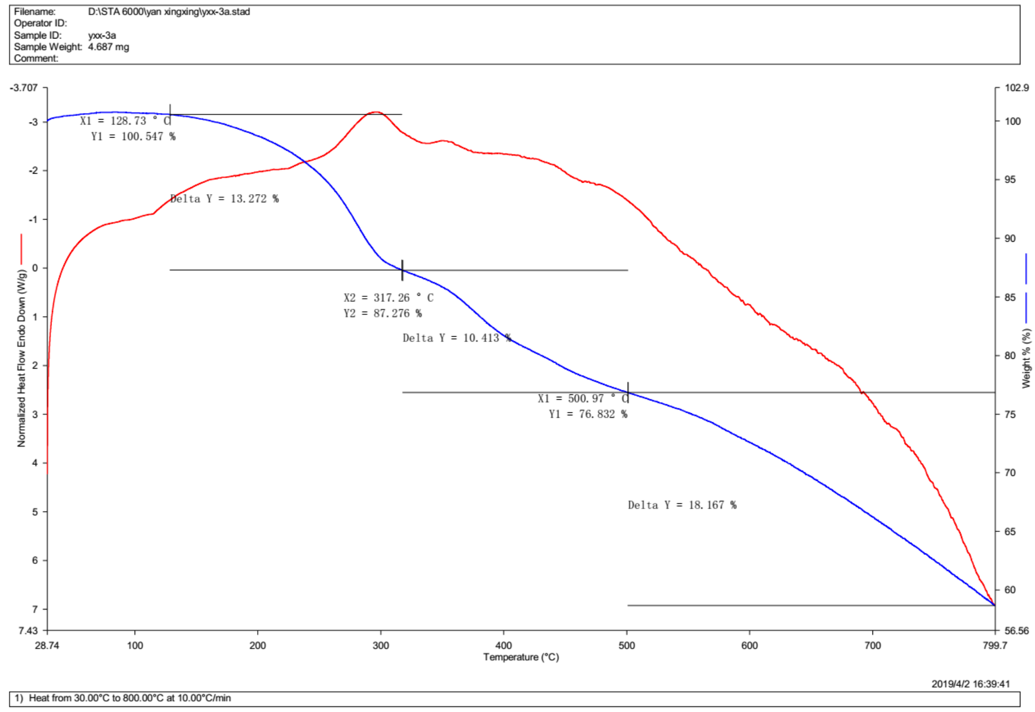

Figure S14. TGA data for 3a under a N_2 gas flow with temperature ramp rate of 10 ^oC/min until 800 ^oC.

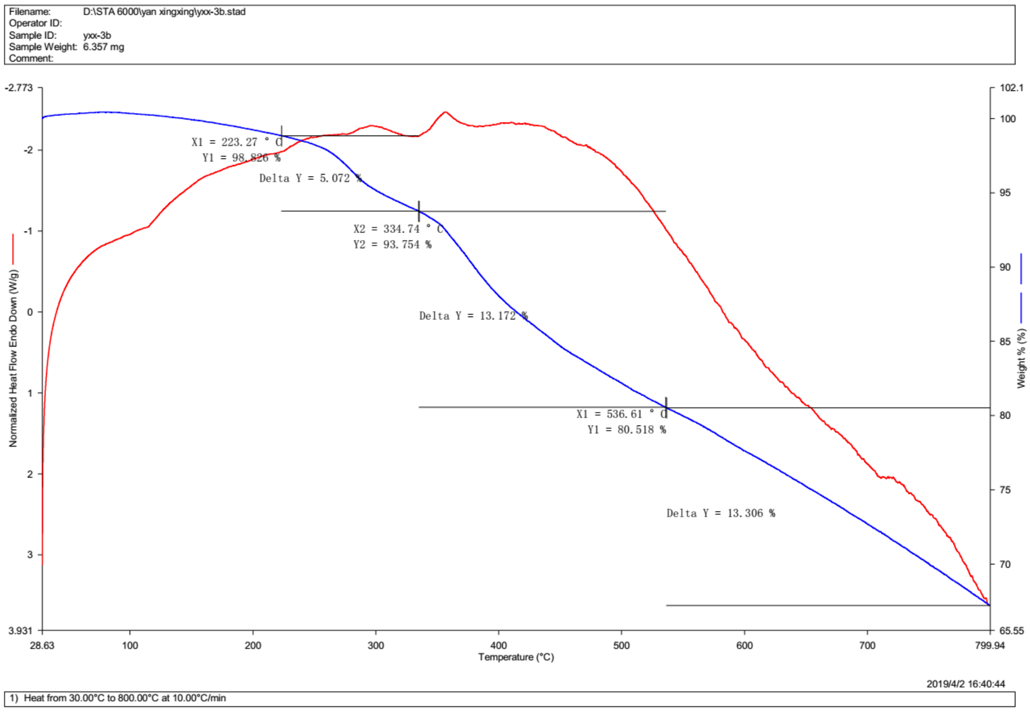

Figure S15. TGA data for 3b under a N_2 gas flow with temperature ramp rate of 10 °C/min until 800 °C.

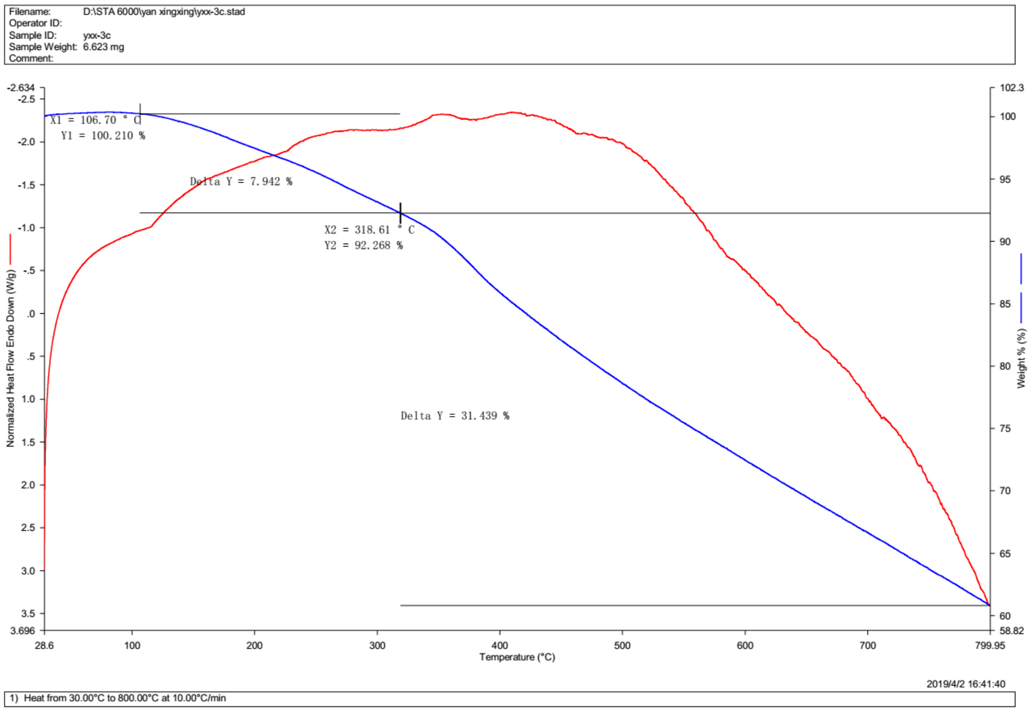

Figure S16. TGA data for 3c under a N_2 gas flow with temperature ramp rate of 10 °C/min until 800 °C.

8. NMR Spectra of Compounds 1b, 1c, 2a–c, IIa, and 3a–c

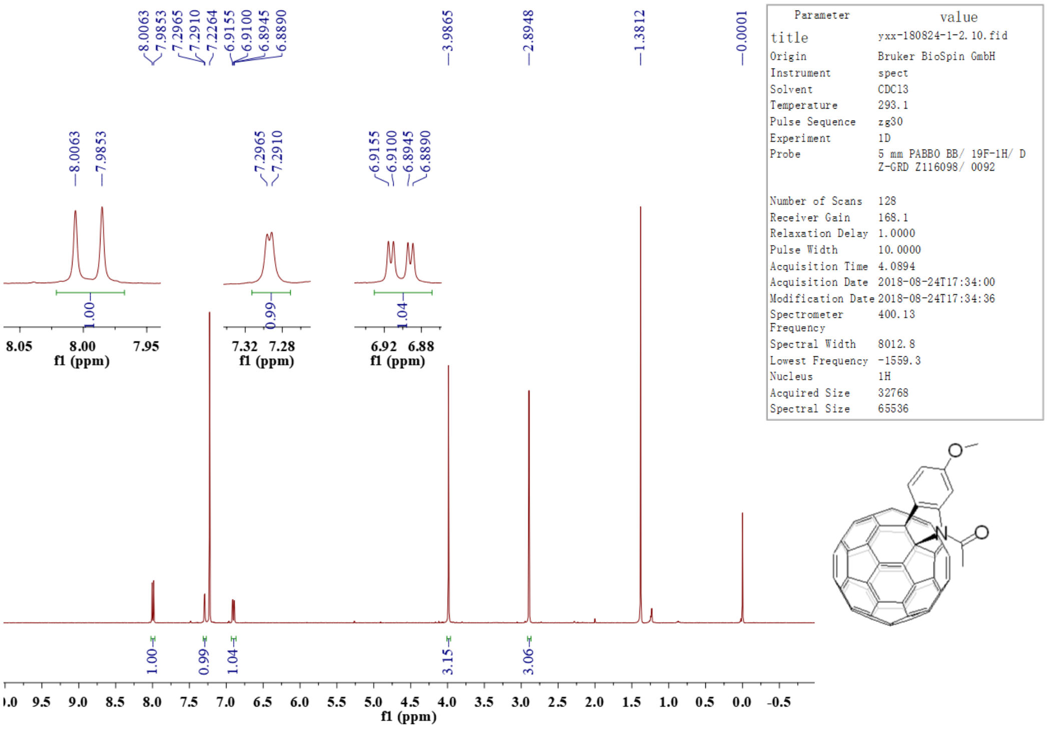

Figure S17 ^{1}H NMR (400 MHz, 1:1 $CS_2/CDCl_3$) of compound 1b

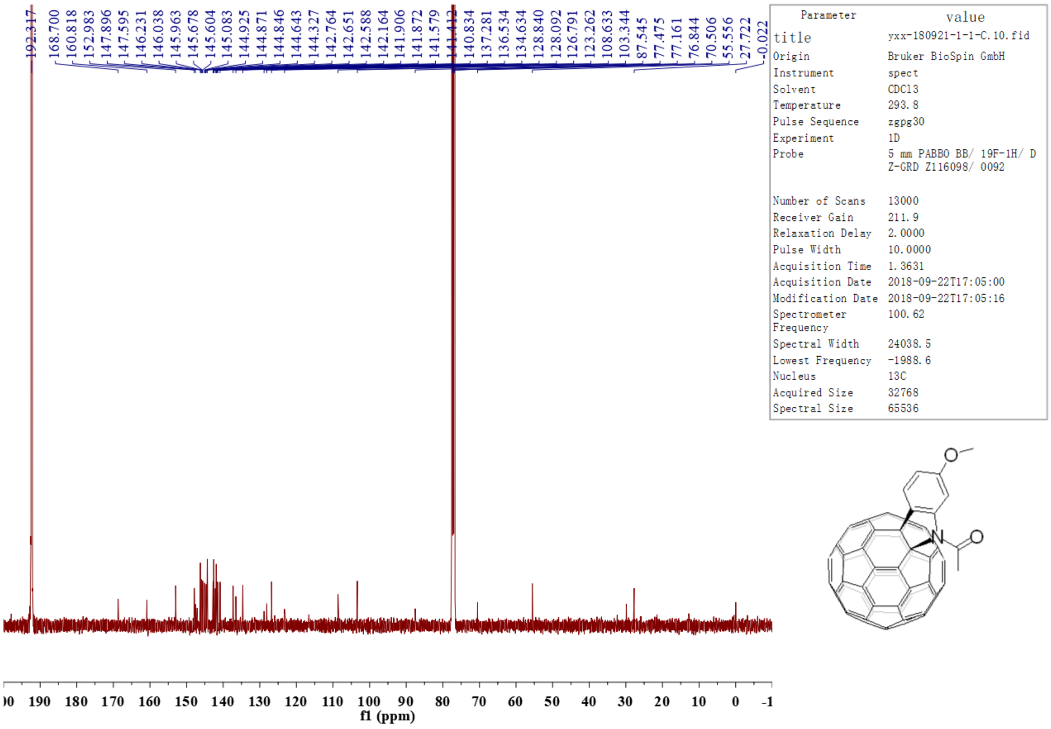

Figure S18 ^{13}C NMR (100 MHz, 1:1 $CS_2/CDCl_3$) of compound 1b

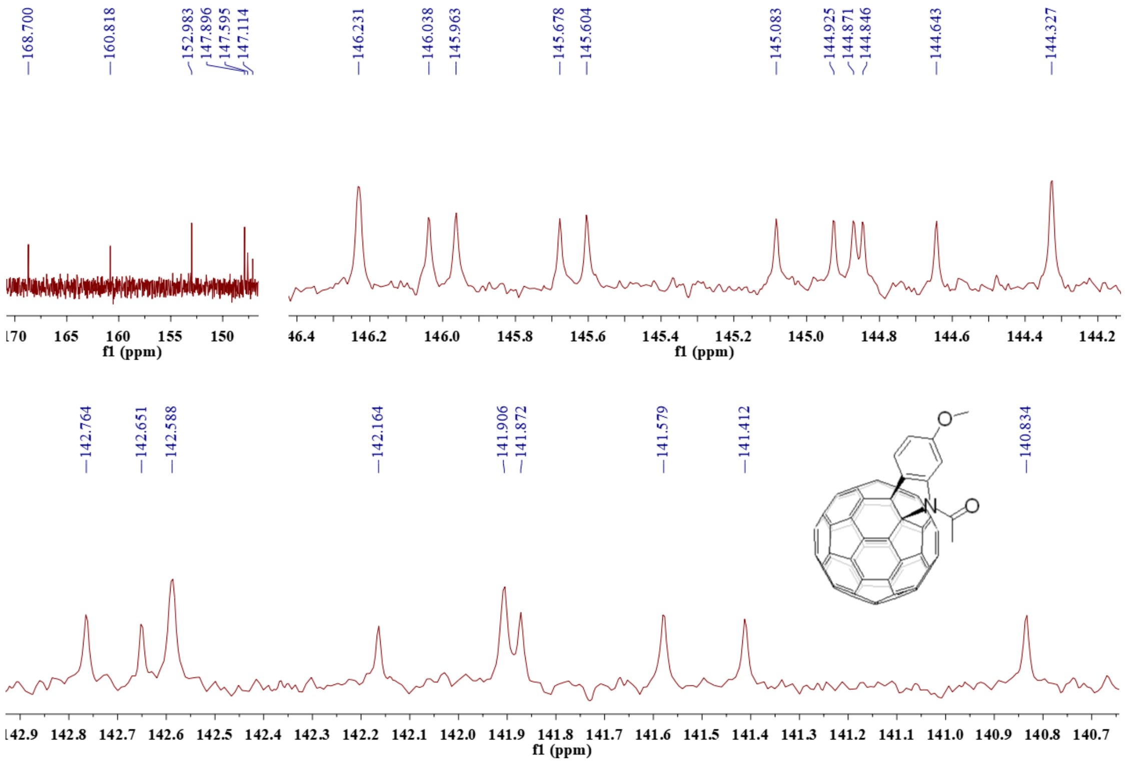

Figure S19 Expanded of ^{13}C NMR (100 MHz, 1:1 $CS_2/CDCl_3$) of compound 1b

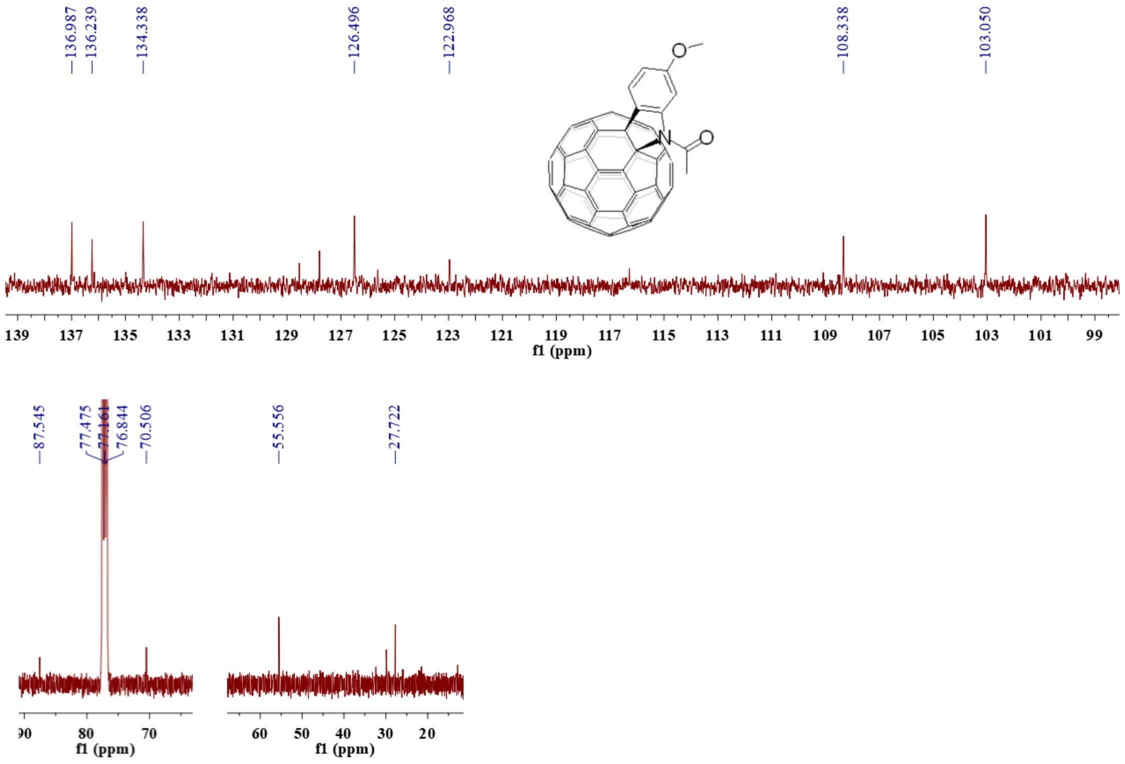

Figure S20 Expanded of ^{13}C NMR (100 MHz, 1:1 $CS_2/CDCl_3$) of compound 1b

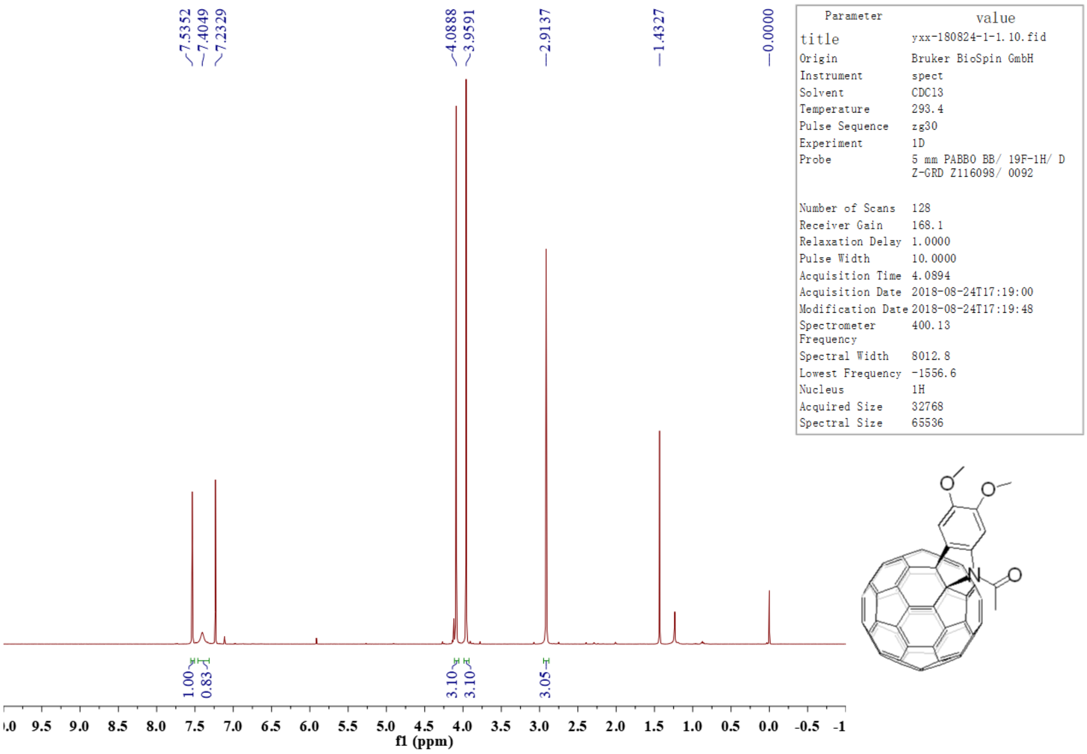

Figure S21 ^{1}H NMR (400 MHz, 1:1 CS_2/$CDCl_3$) of compound 1c

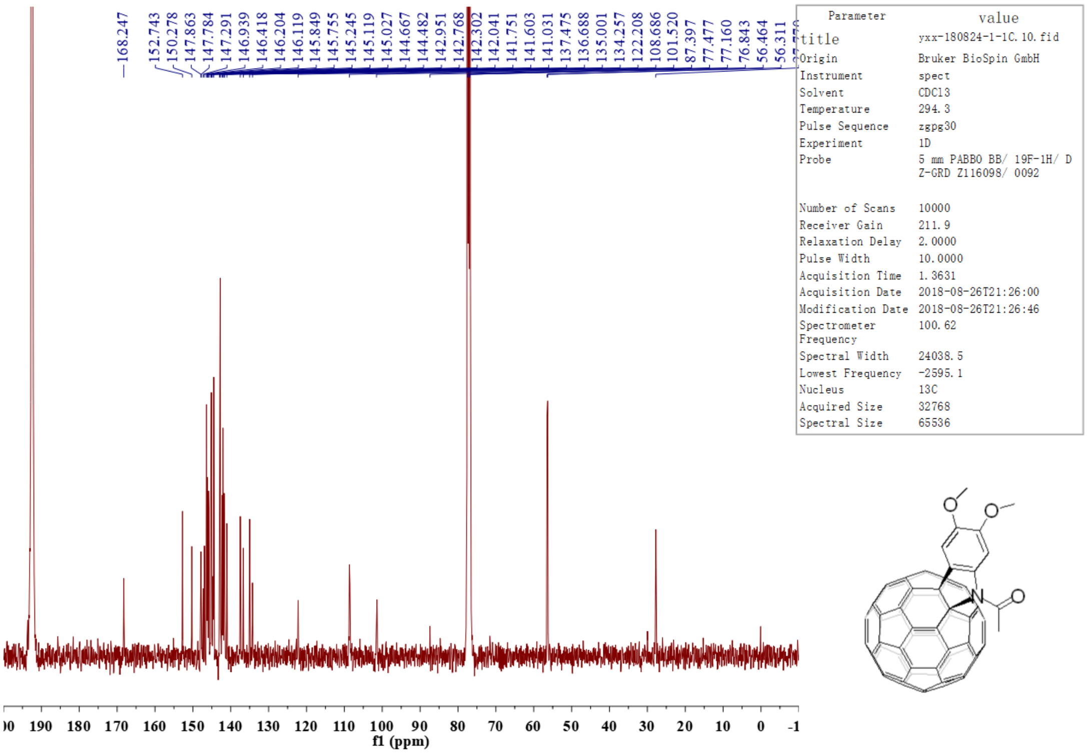

Figure S22 ^{13}C NMR (100 MHz, 1:1 CS_2/$CDCl_3$) of compound 1c

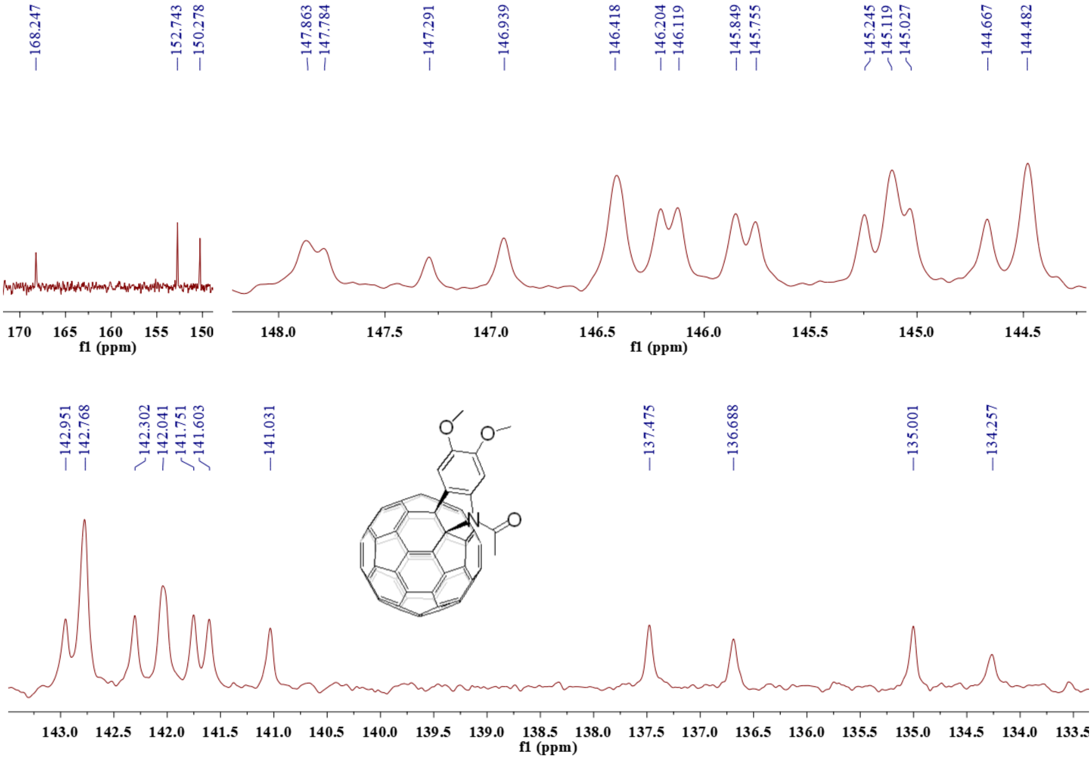

Figure S23 Expanded of ^{13}C NMR (100 MHz, 1:1 $CS_2/CDCl_3$) of compound 1c

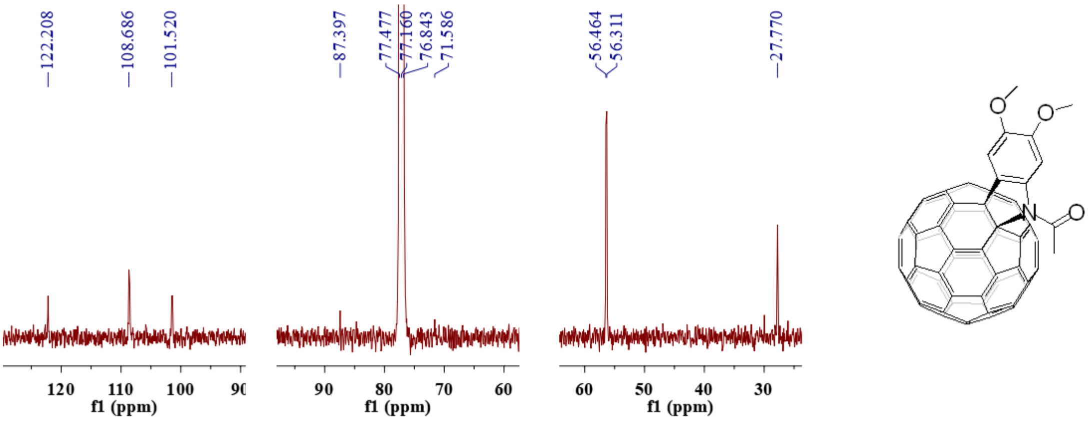

Figure S24 Expanded of ^{13}C NMR (100 MHz, 1:1 $CS_2/CDCl_3$) of compound 1c

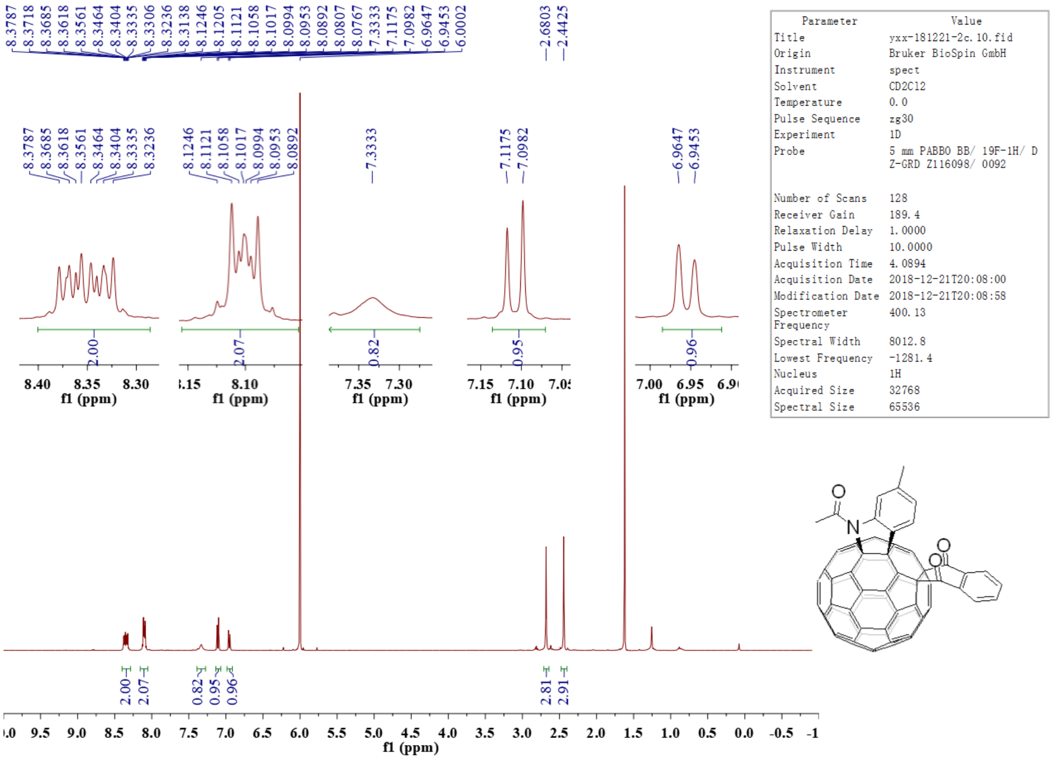

Figure S25 ^{1}H NMR (400 MHz, TCE-d_2) of compound 2a

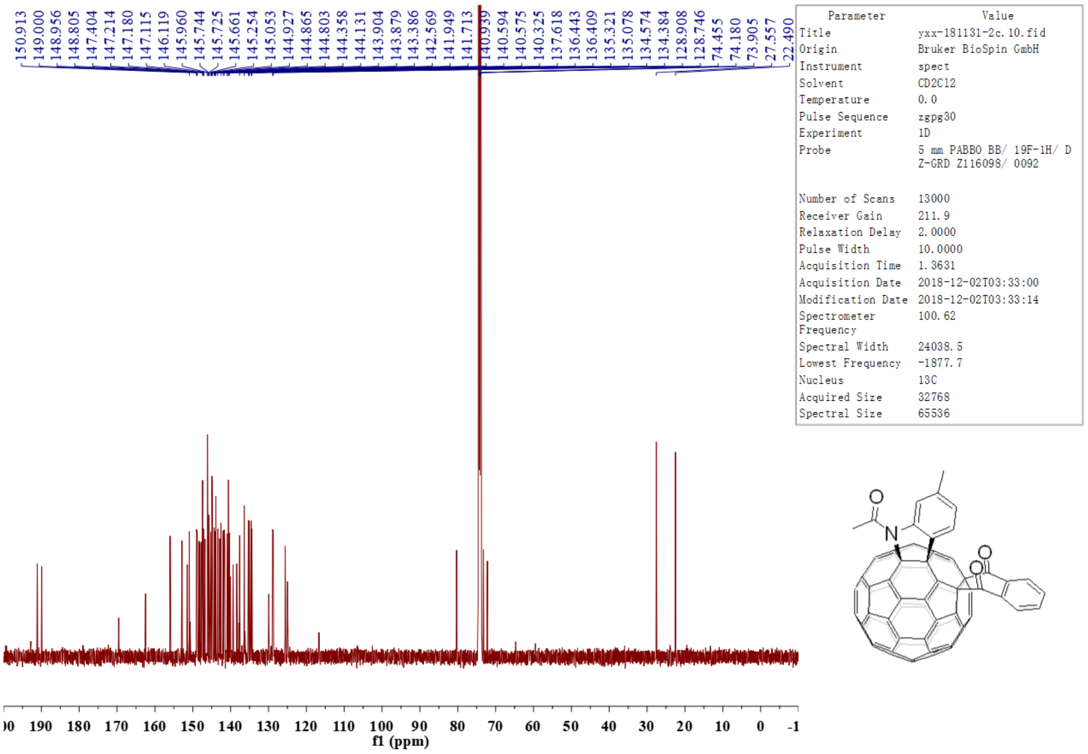

Figure S26 ^{13}C NMR (100 MHz, TCE-d_2) of compound 2a

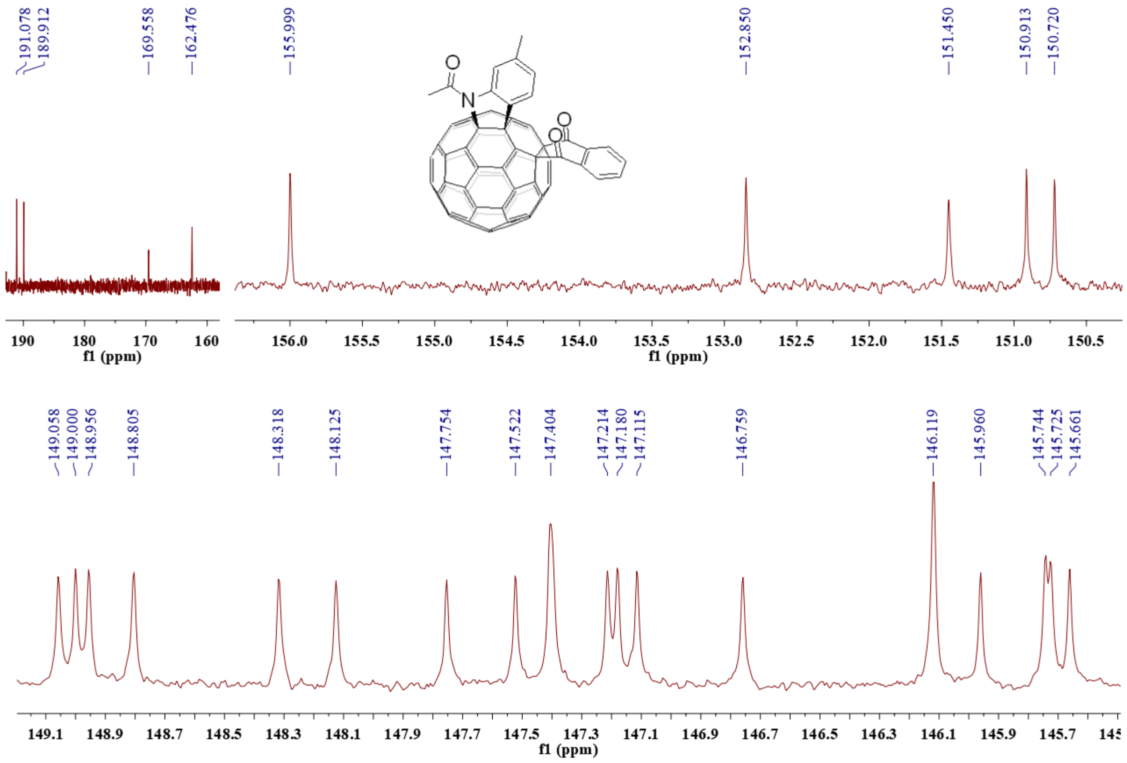

Figure S27 Expanded of ^{13}C NMR (100 MHz, TCE-*d*$_2$) of compound 2a

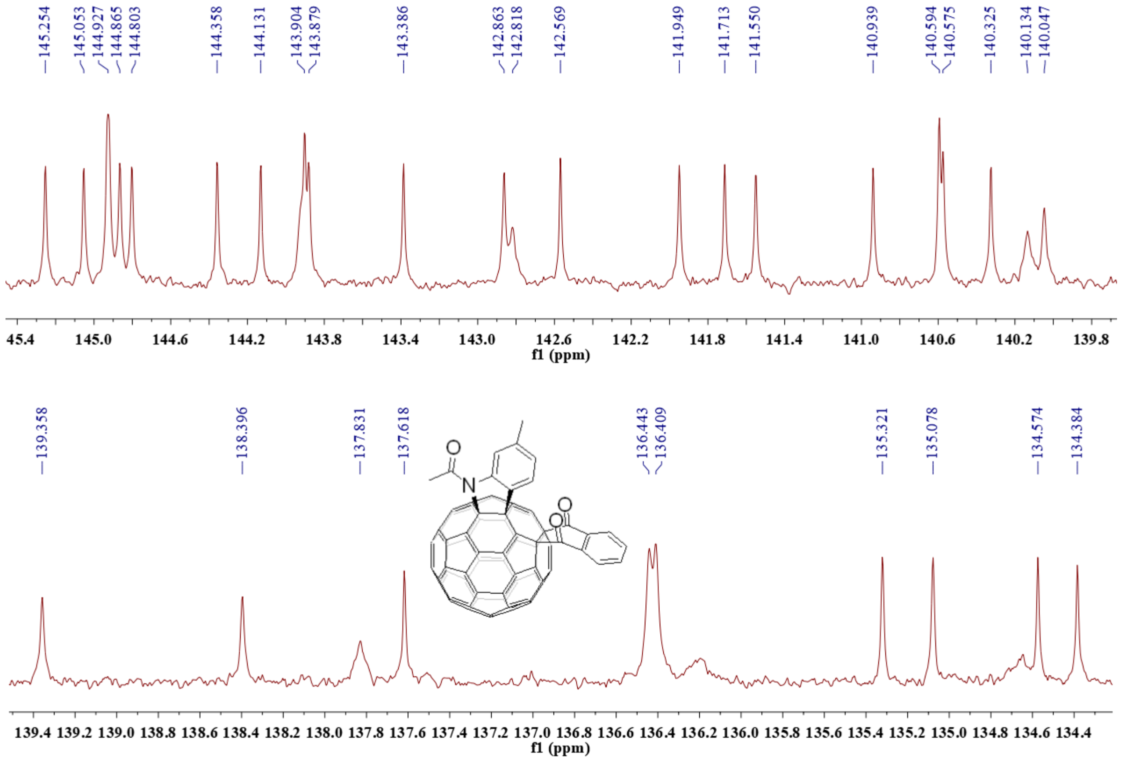

Figure S28 Expanded of ^{13}C NMR (100 MHz, TCE-*d*$_2$) of compound 2a

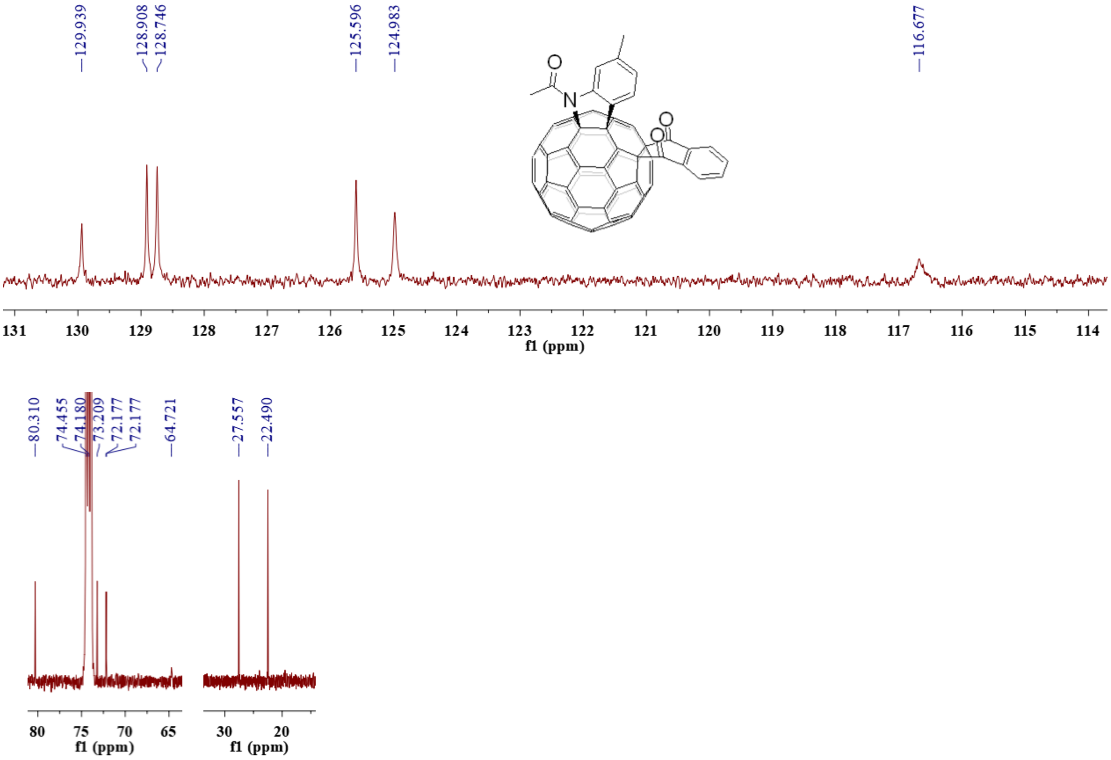

Figure S29 Expanded of ^{13}C NMR (100 MHz, TCE-d_2) of compound 2a

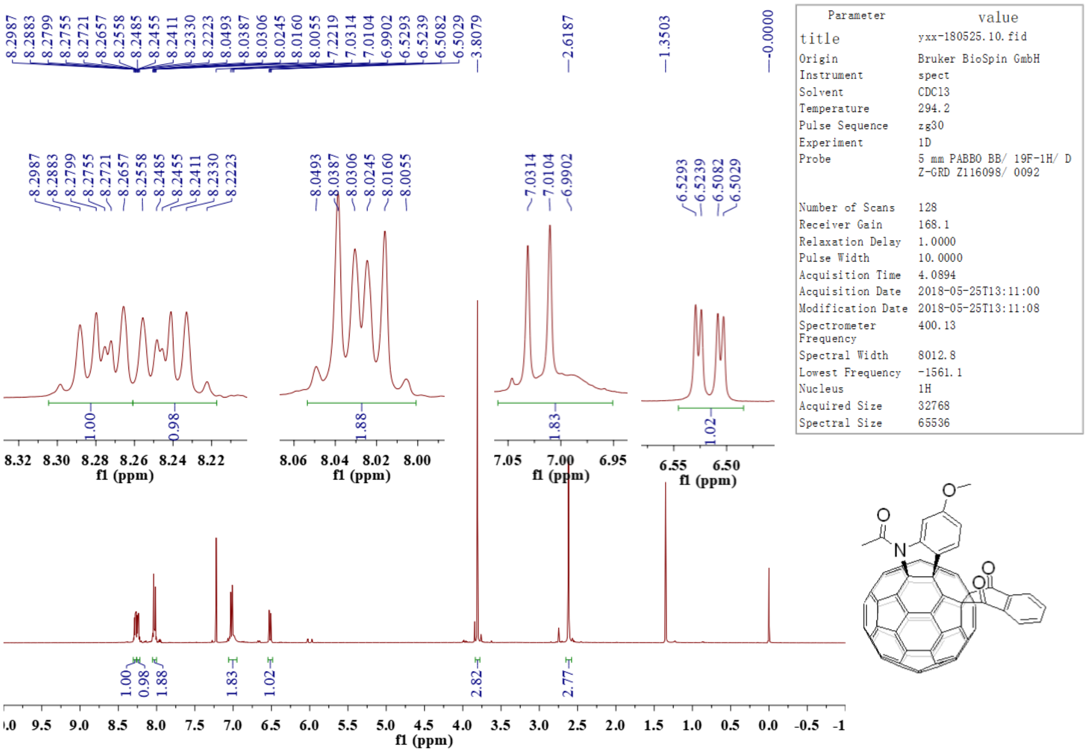

Figure S30 ^{1}H NMR (400 MHz, 1:1 CS_2/$CDCl_3$) of compound 2b

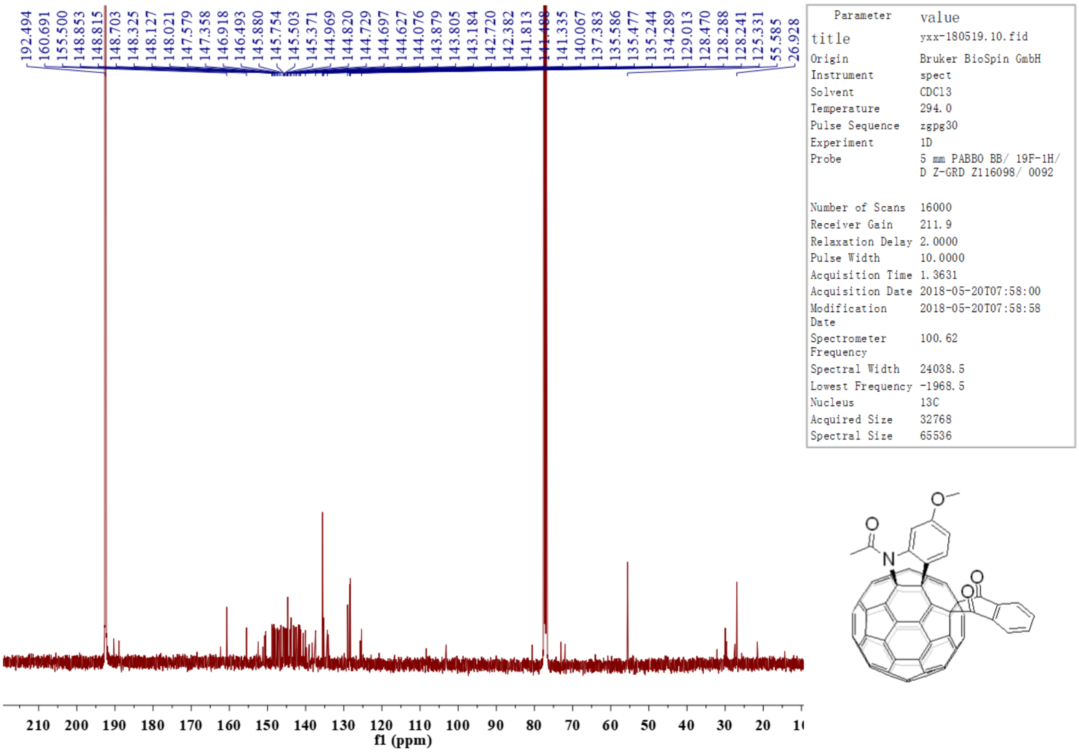

Figure S31 ^{13}C NMR (100 MHz, 1:1 CS_2/$CDCl_3$) of compound 2b

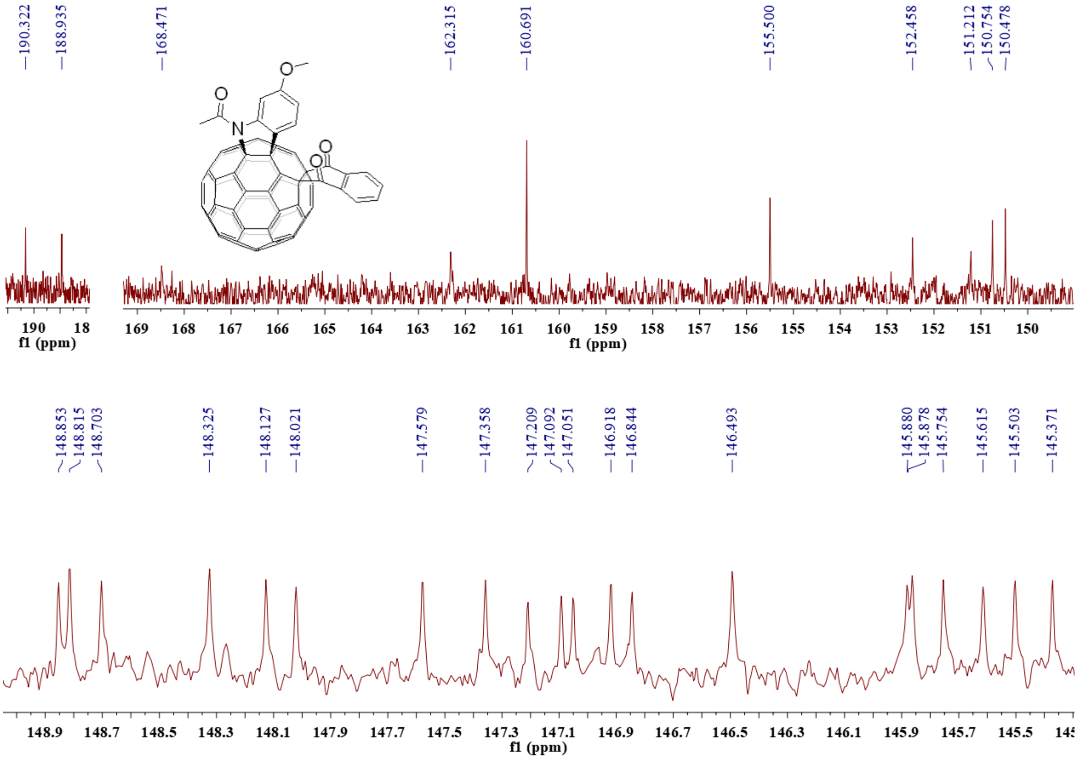

Figure S32 Expanded of ^{13}C NMR (100 MHz, 1:1 CS_2/$CDCl_3$) of compound 2b

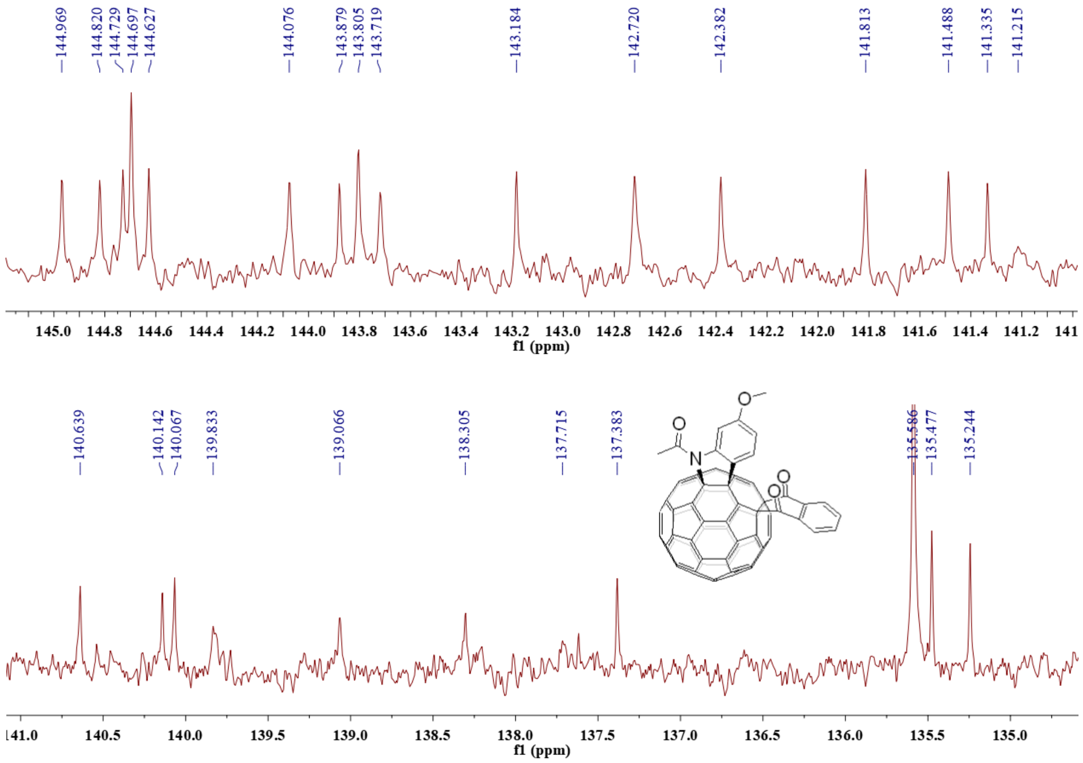

Figure S33 Expanded of ^{13}C NMR (100 MHz, 1:1 $CS_2/CDCl_3$) of compound 2b

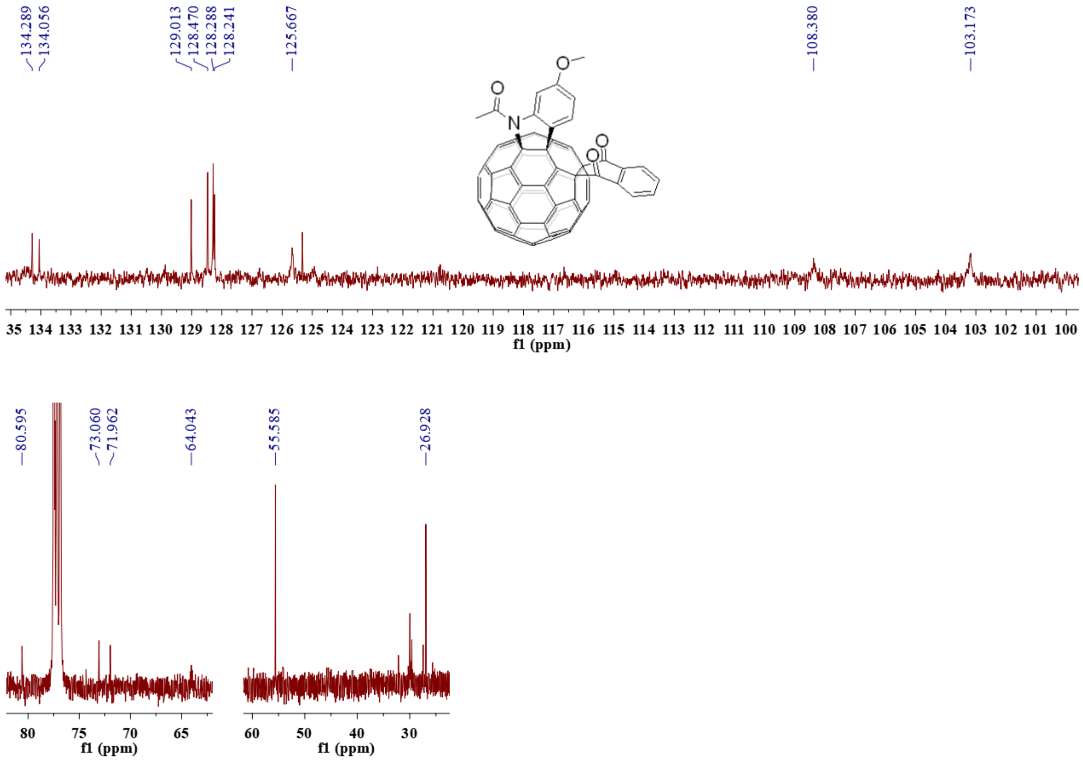

Figure S34 Expanded of ^{13}C NMR (100 MHz, 1:1 $CS_2/CDCl_3$) of compound 2b

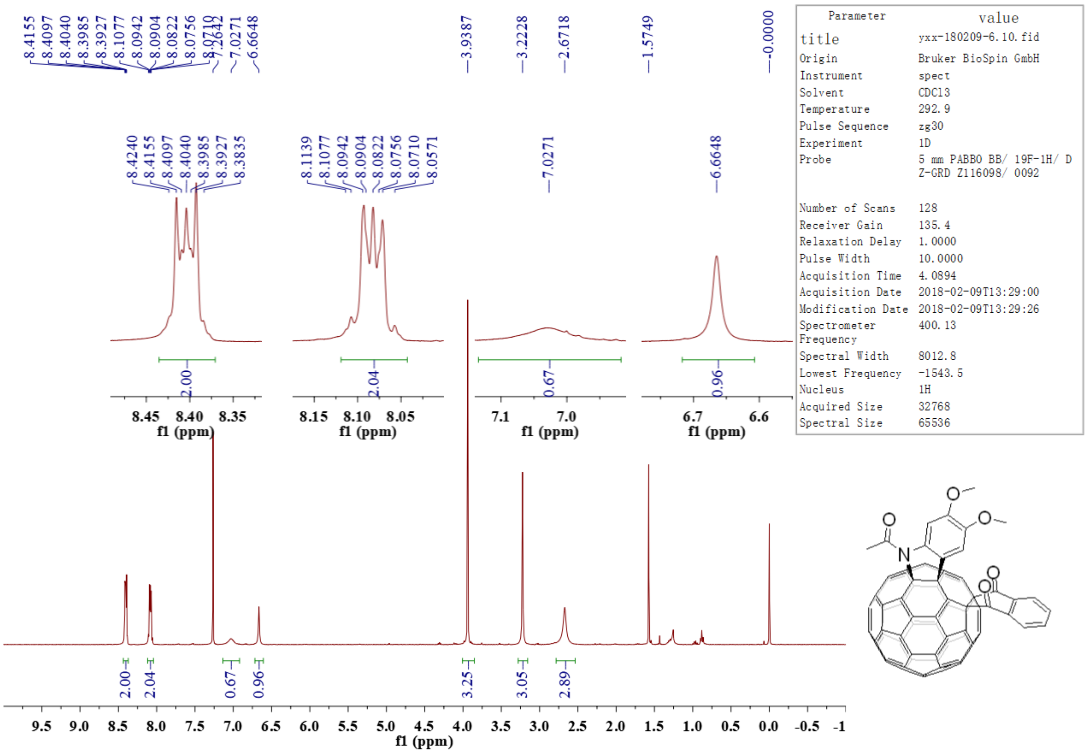

Figure S35 ^{1}H NMR (400 MHz, 1:1 CS_2/$CDCl_3$) of compound 2c

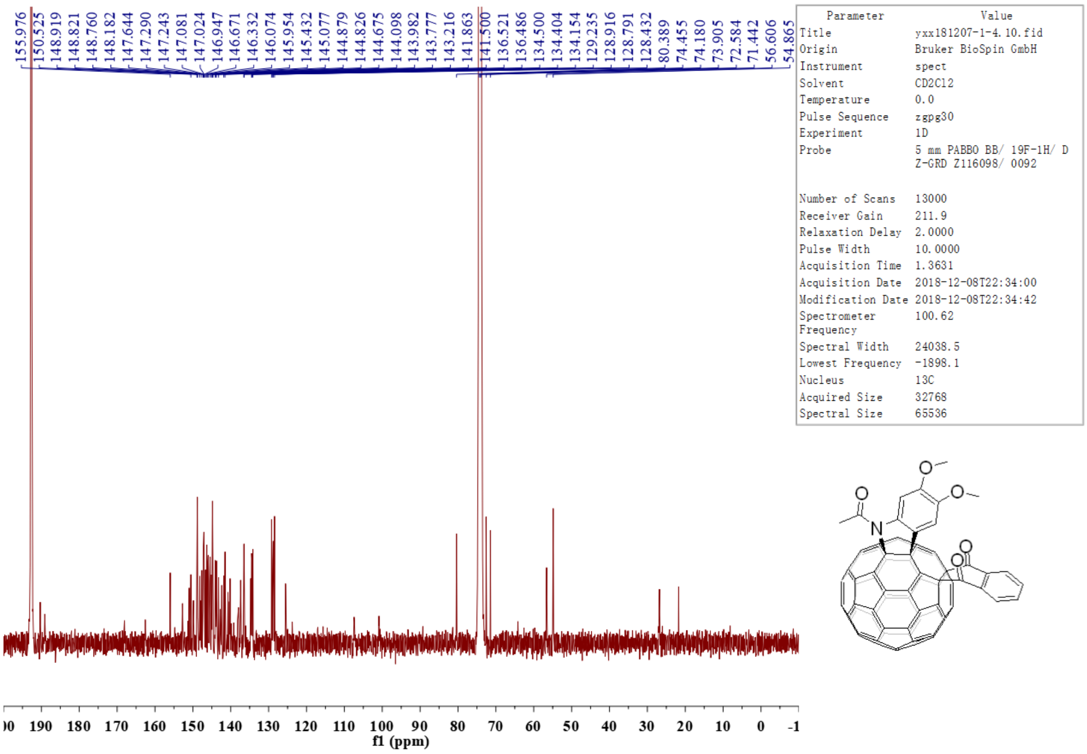

Figure S36 ^{13}C NMR (100 MHz, 1:1 CS_2/TCE-d_2) of compound 2c

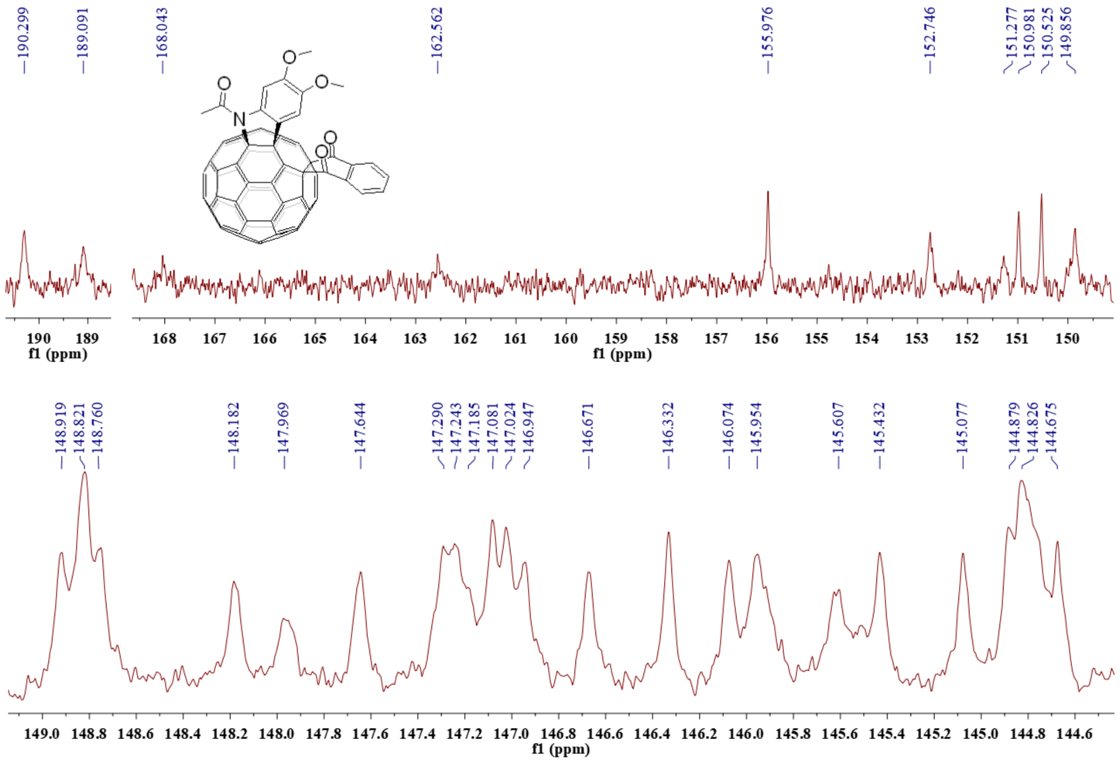

Figure S37 Expanded of ^{13}C NMR (100 MHz, 1:1 CS_2/TCE-d_2) of compound 2c

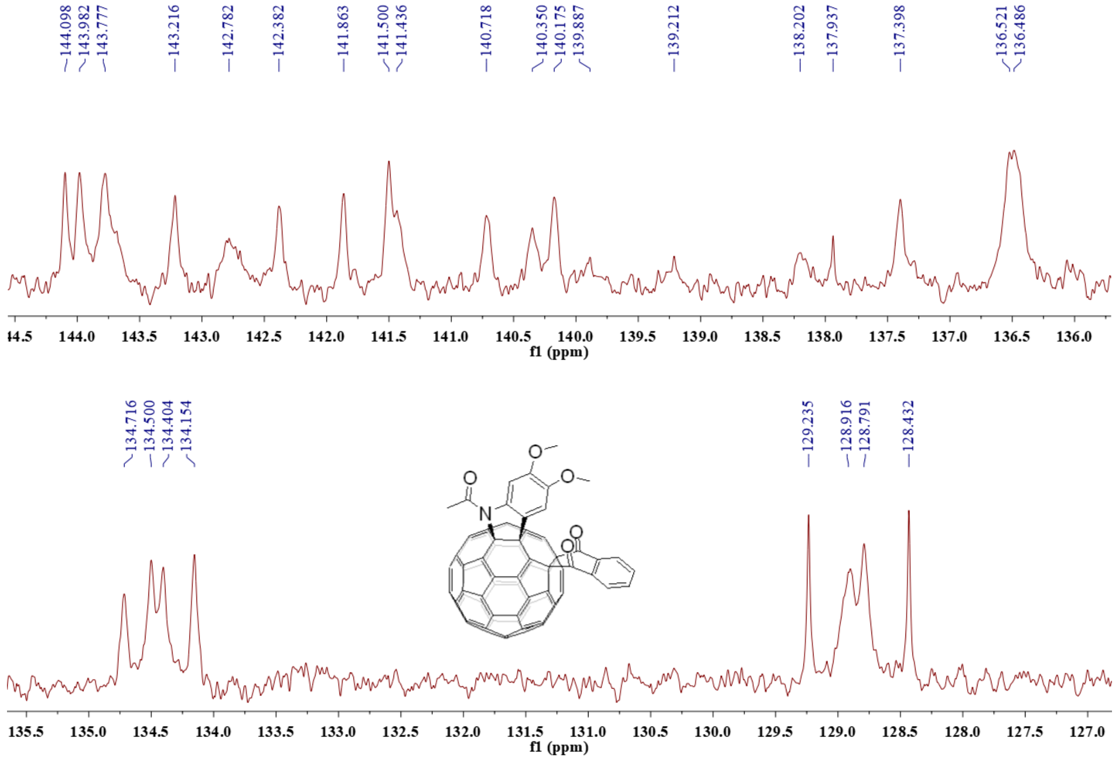

Figure S38 Expanded of ^{13}C NMR (100 MHz, 1:1 CS_2/TCE-d_2) of compound 2c

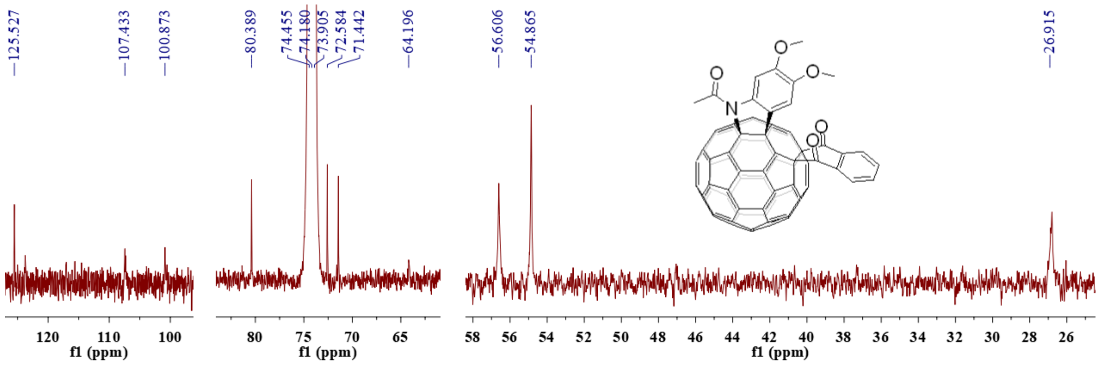

Figure S39 Expanded of ^{13}C NMR (100 MHz, 1:1 CS_2/TCE-d_2) of compound 2c

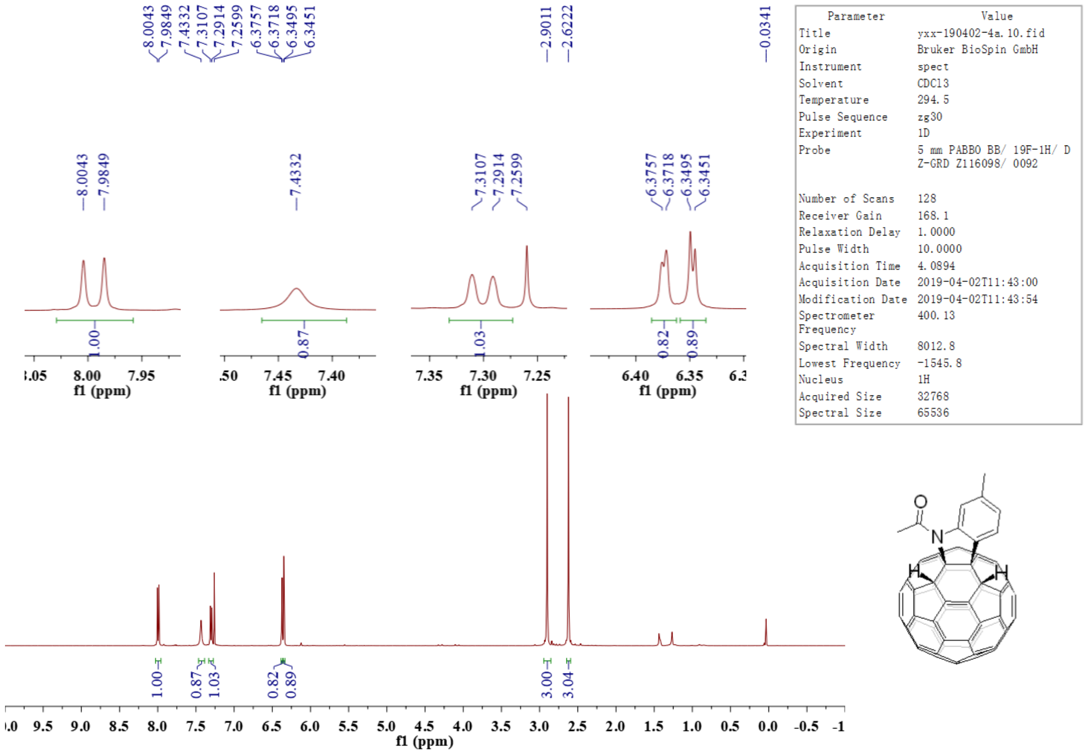

Figure S40 ^{1}H NMR (400 MHz, 1:1 CS_2/$CDCl_3$) of compound IIa

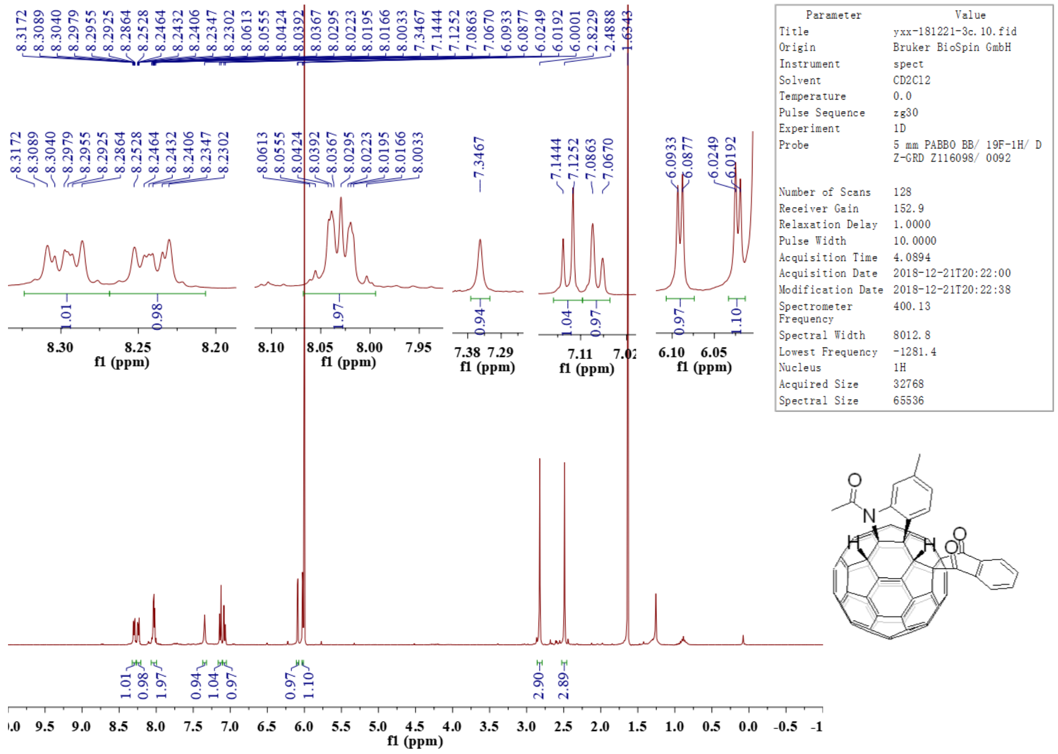

Figure S41 ^{1}H NMR (400 MHz, TCE-d_2) of compound 3a

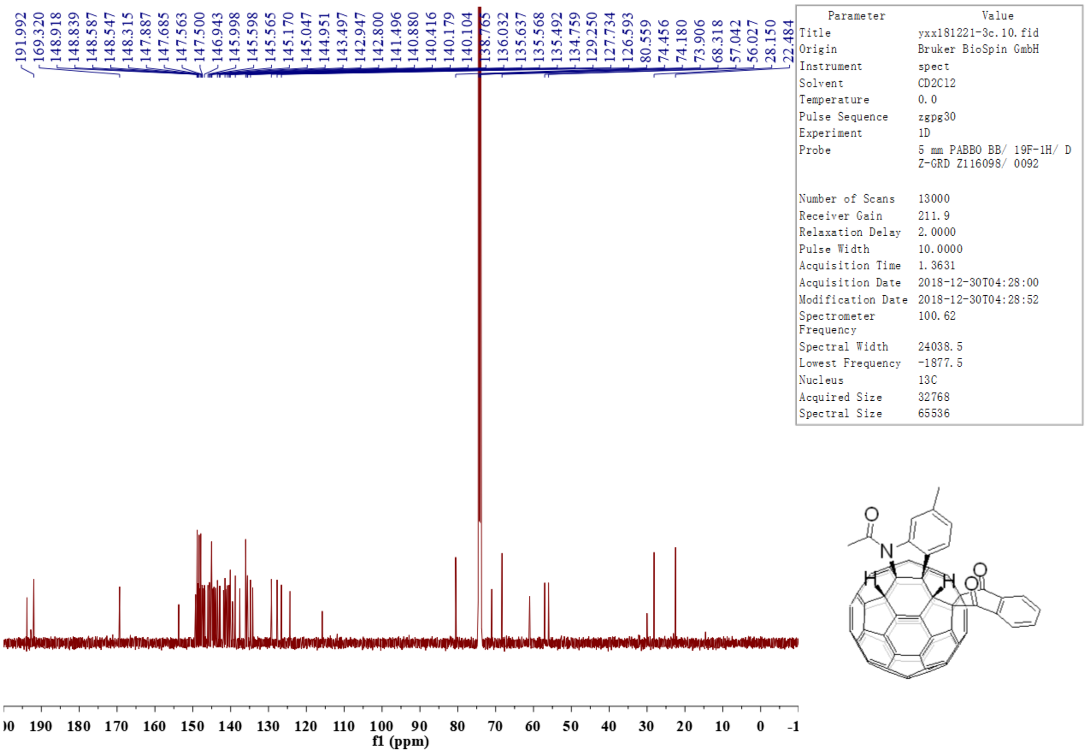

Figure S42 ^{13}C NMR (100 MHz, TCE-d_2) of compound 3a

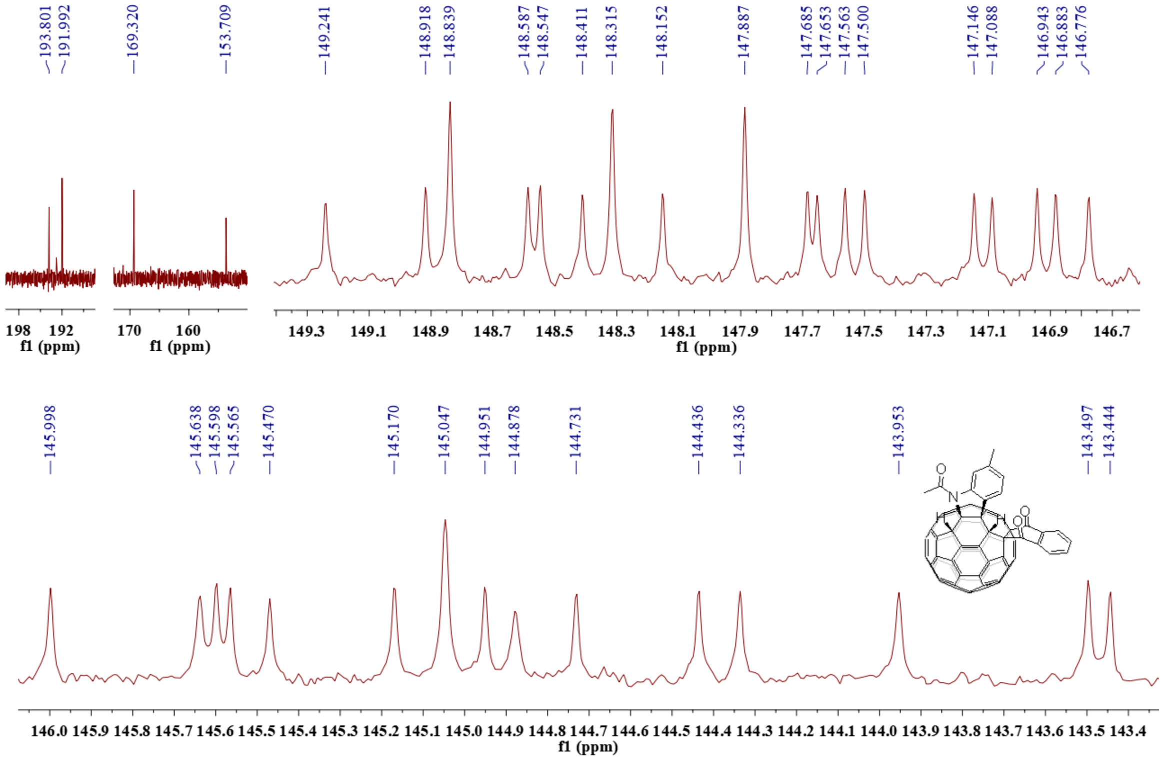

Figure S43 Expanded of ^{13}C NMR (100 MHz, TCE-d_2) of compound 3a

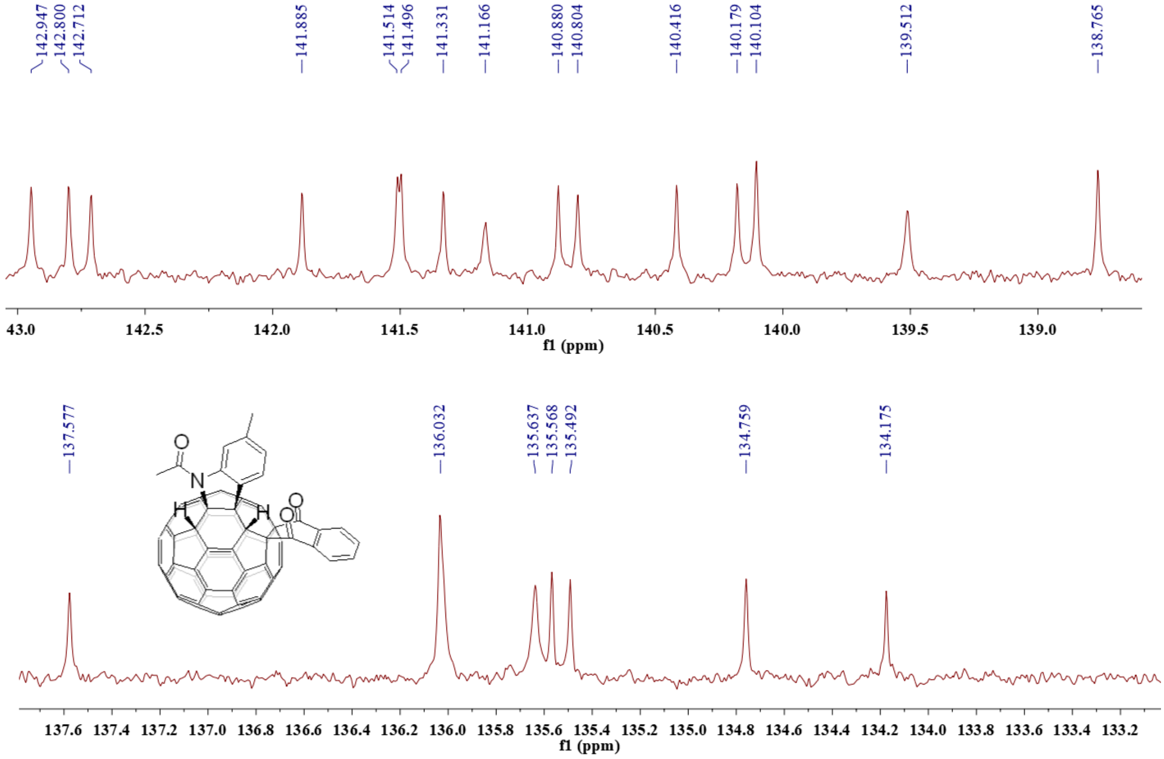

Figure S44 Expanded of ^{13}C NMR (100 MHz, TCE-d_2) of compound 3a

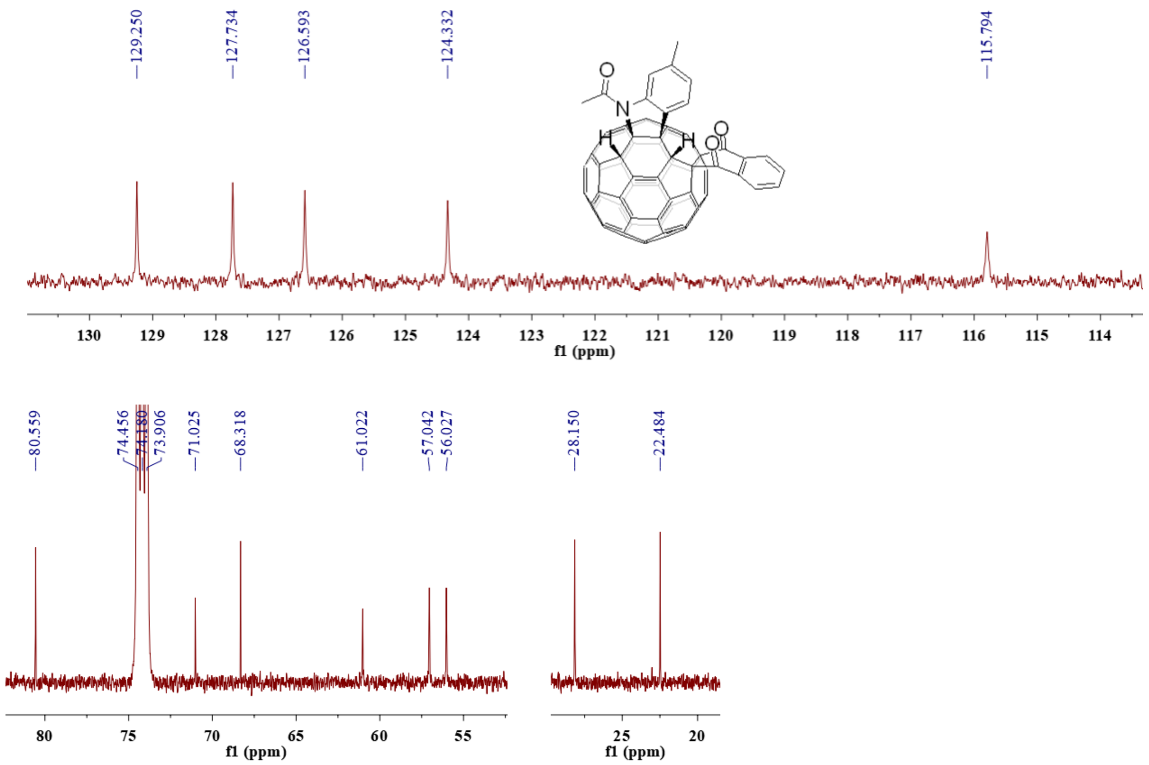

Figure S45 Expanded of ^{13}C NMR (100 MHz, TCE-d_2) of compound 3a

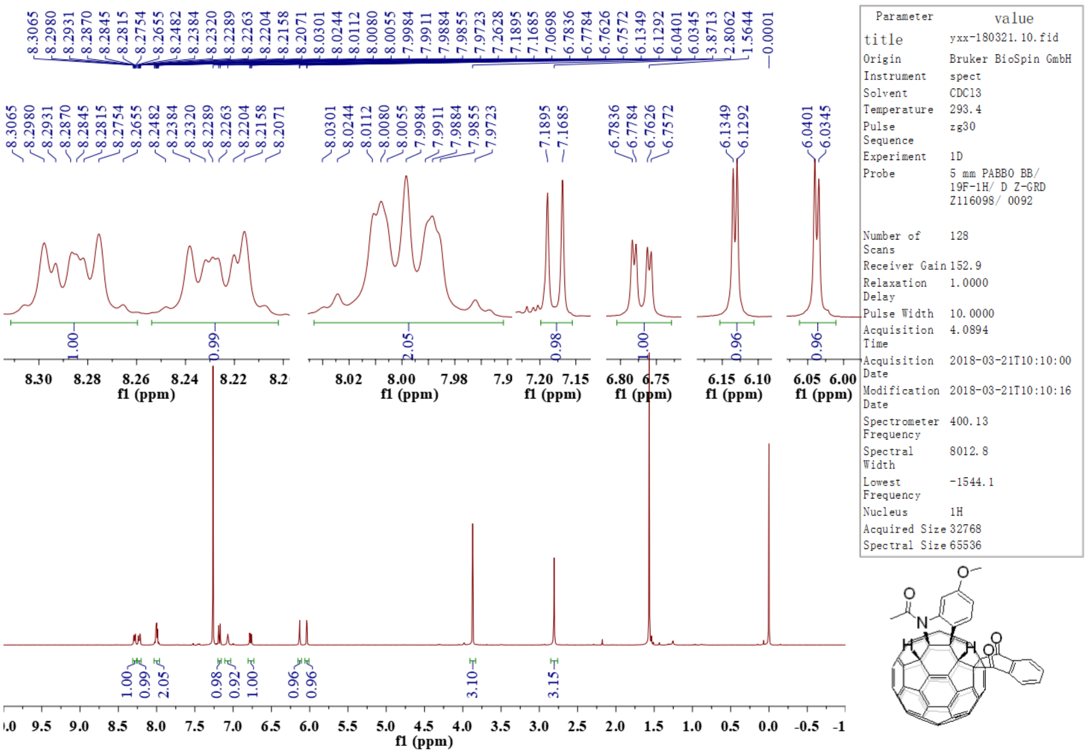

Figure S46 ^{1}H NMR (400 MHz, 1:1 CS_2/$CDCl_3$) of compound 3b

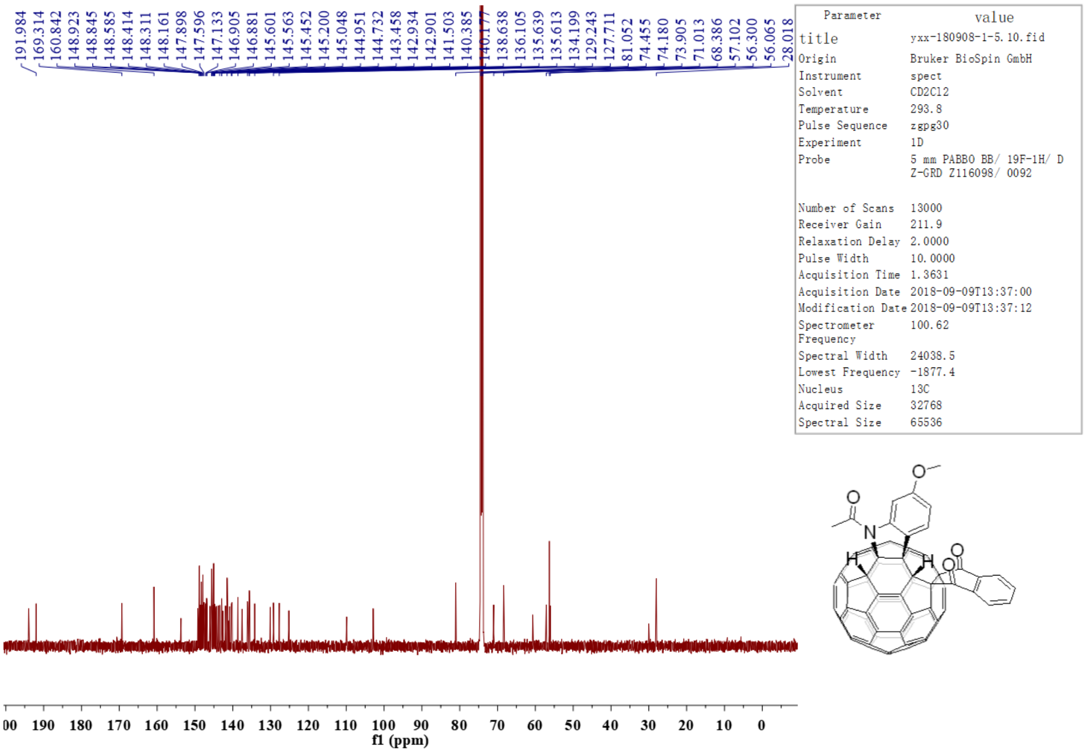

Figure S47 ^{13}C NMR (100 MHz, TCE-d_2) of compound 3b

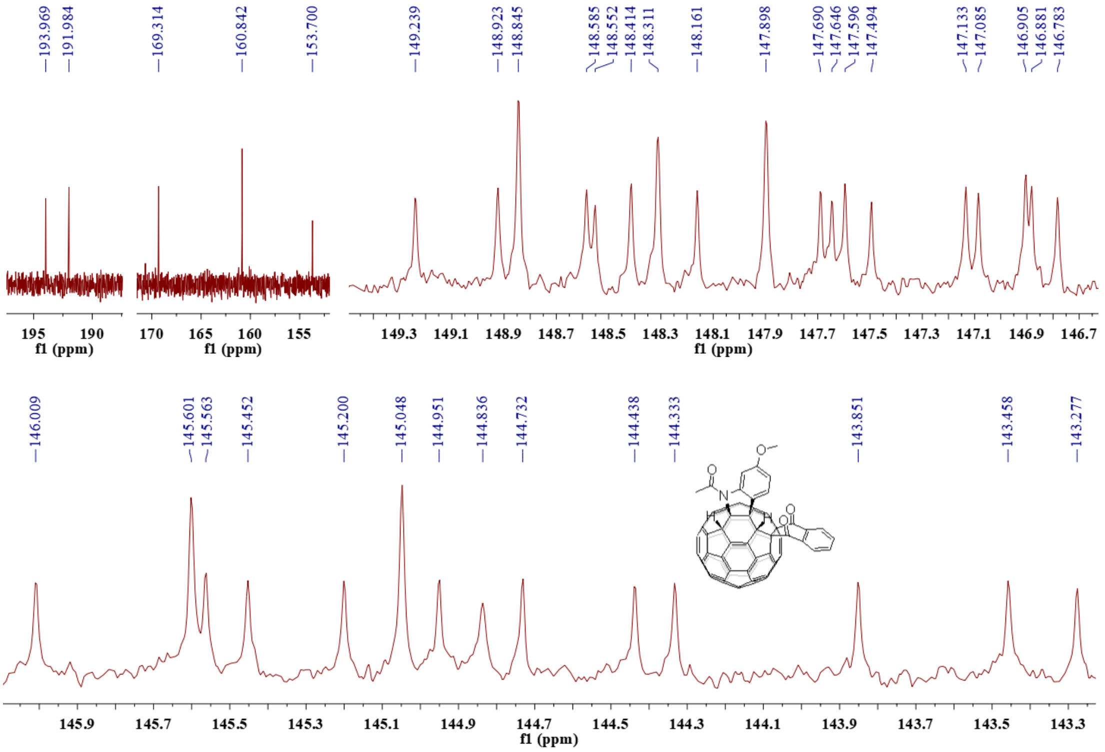

Figure S48 Expanded of ^{13}C NMR (100 MHz, TCE-d_2) of compound 3b

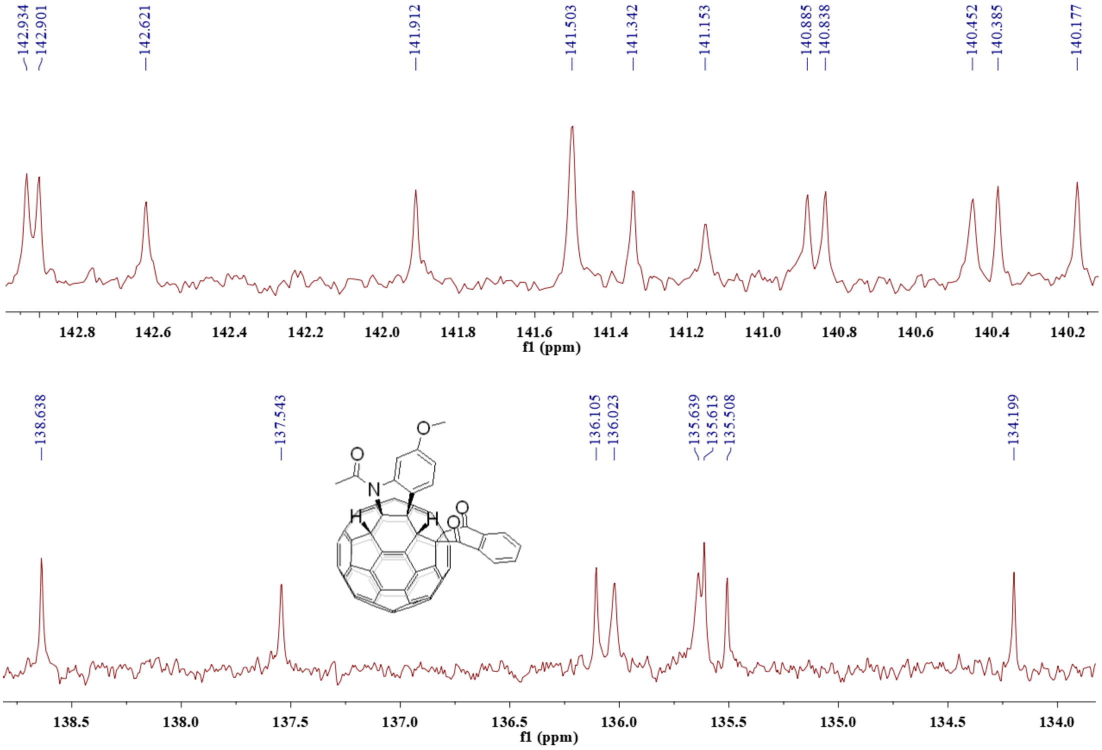

Figure S49 Expanded of ^{13}C NMR (100 MHz, TCE-*d*$_2$) of compound 3b

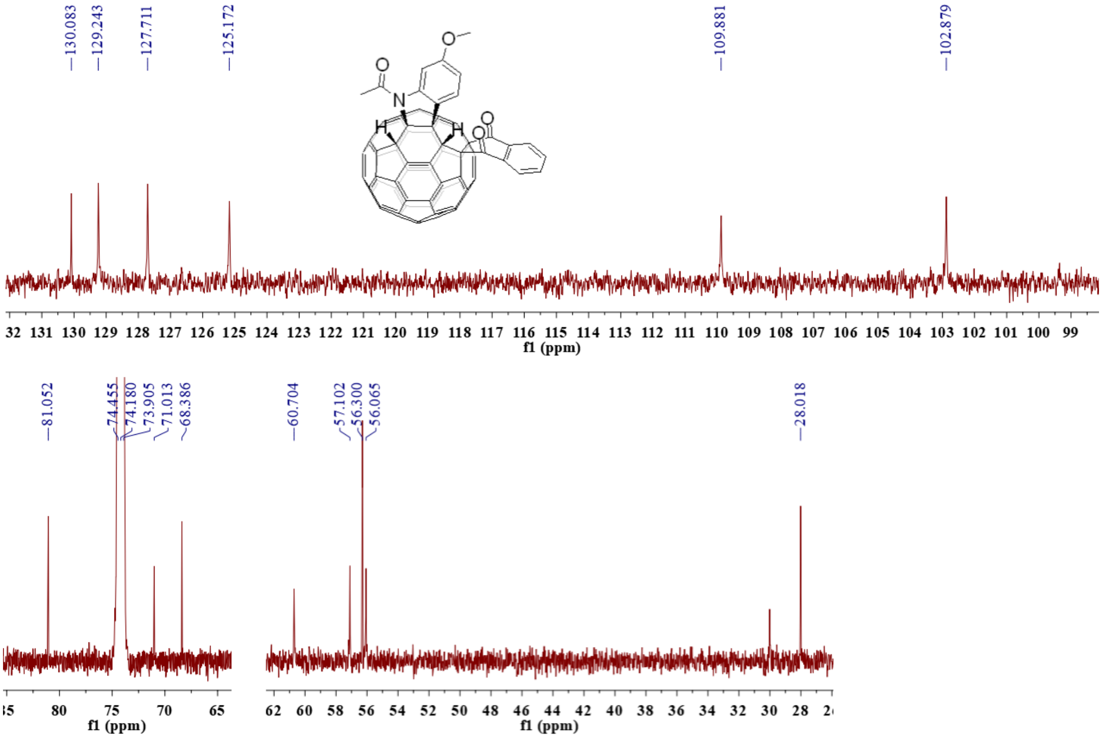

Figure S50 Expanded of ^{13}C NMR (100 MHz, TCE-*d*$_2$) of compound 3b

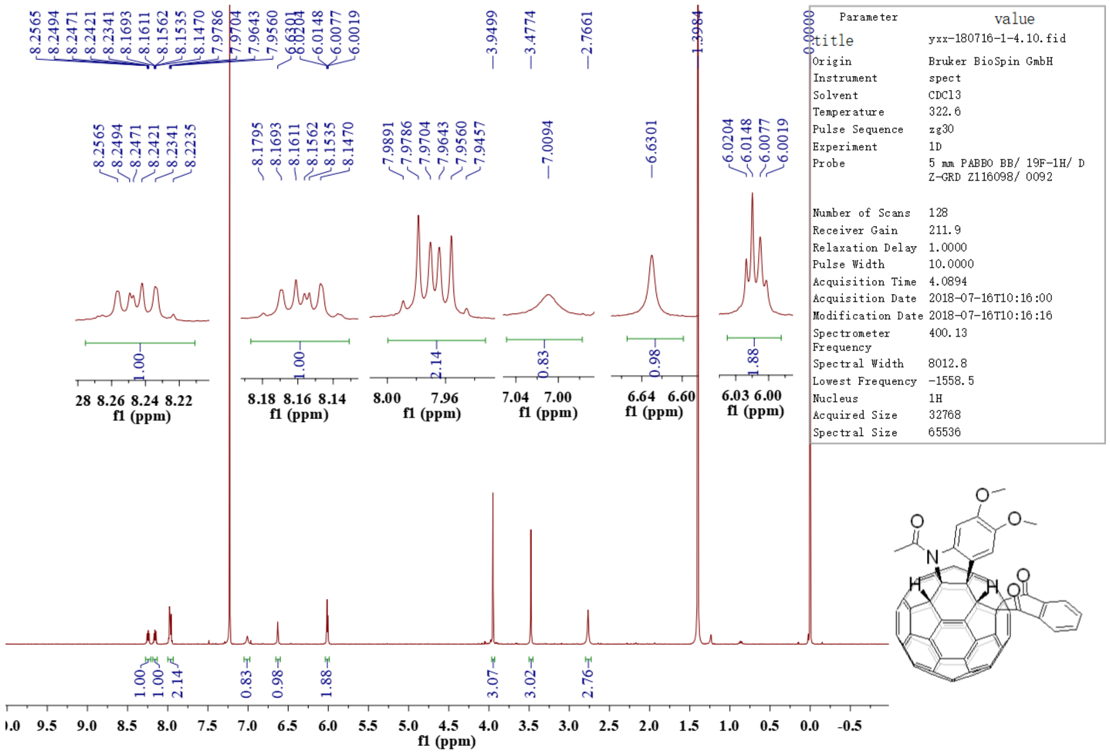

Figure S51 ^{1}H NMR (400 MHz, 1:1 $CS_2/CDCl_3$) of compound 3c

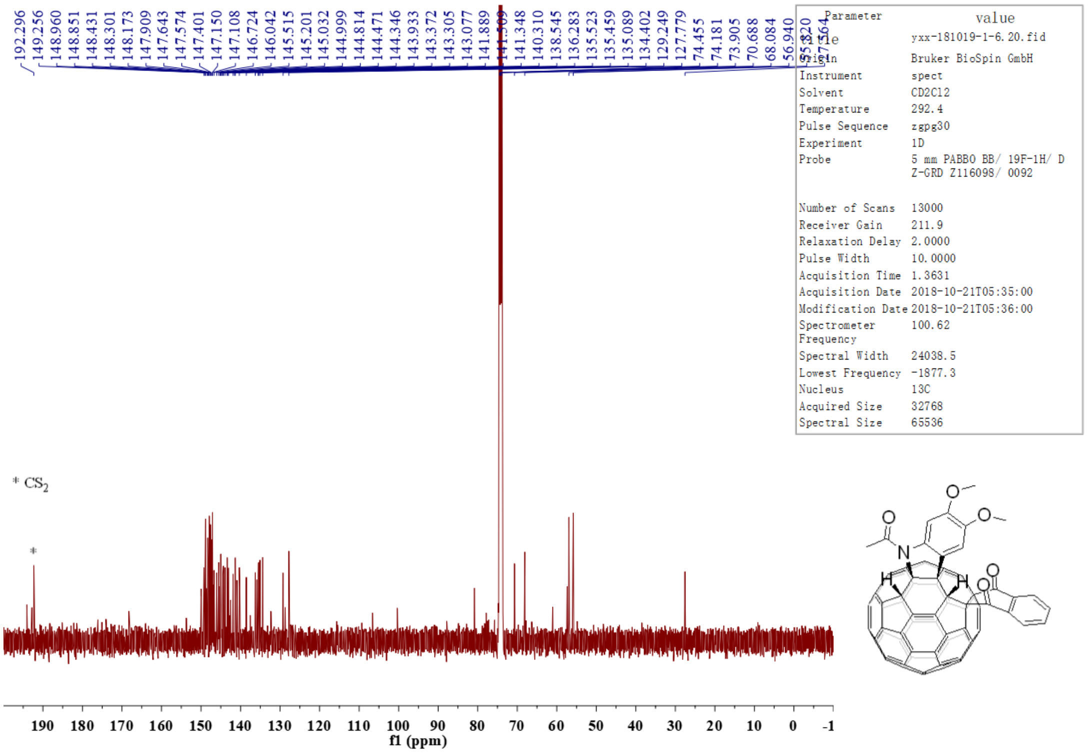

Figure S52 ^{13}C NMR (100 MHz, TCE-d_2) of compound 3c

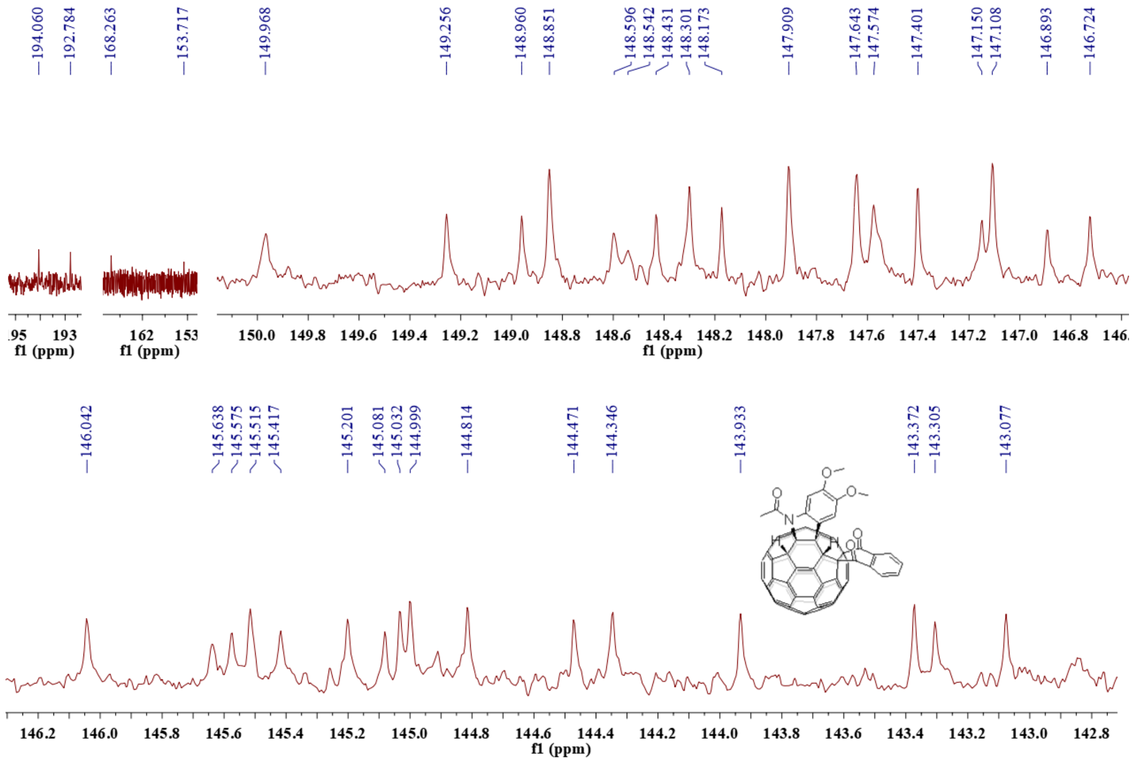

Figure S53 Expanded of ^{13}C NMR (100 MHz, TCE-d_2) of compound 3c

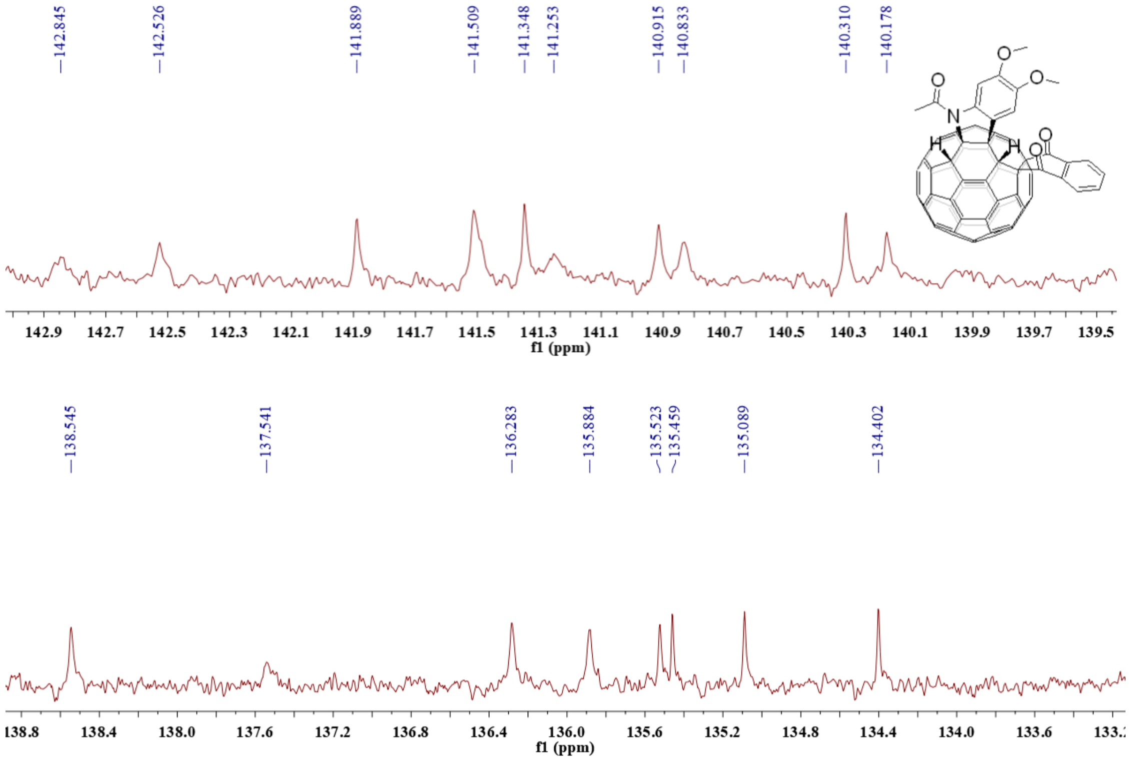

Figure S54 Expanded of ^{13}C NMR (100 MHz, TCE-d_2) of compound 3c

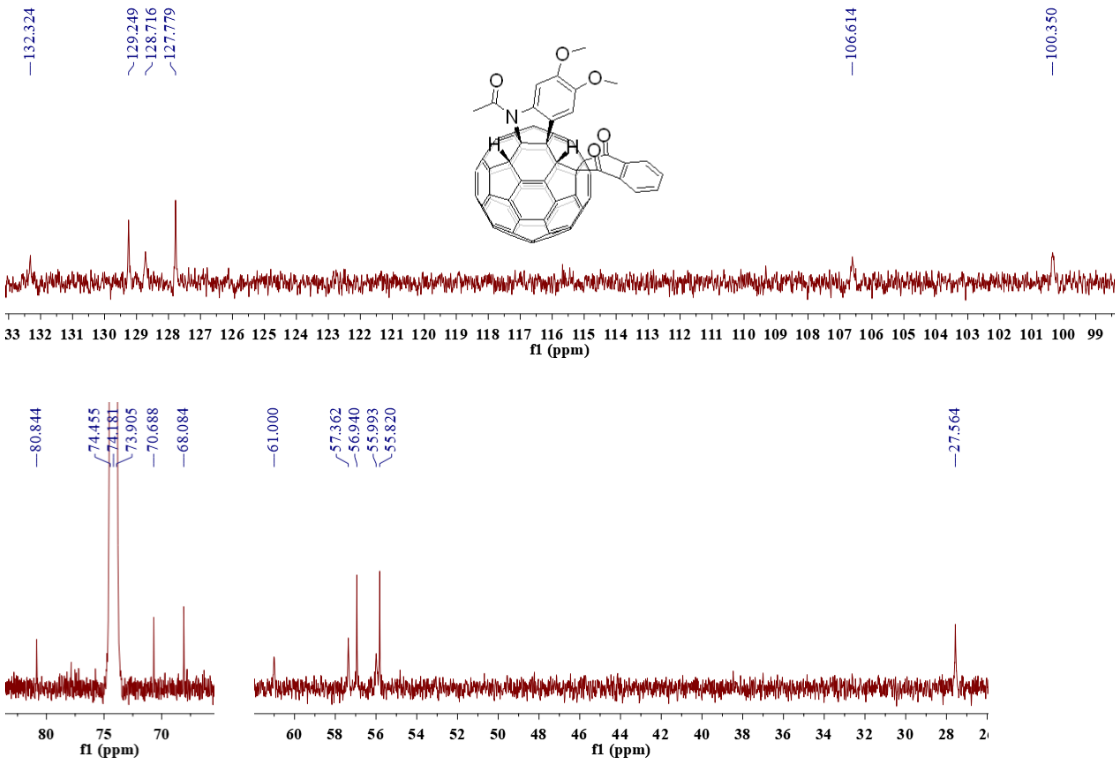

Figure S55 Expanded of ^{13}C NMR (100 MHz, TCE-d_2) of compound 3c

9. UV-vis Spectra of Compounds 1b, 1c, 2a-c, and 3a-c

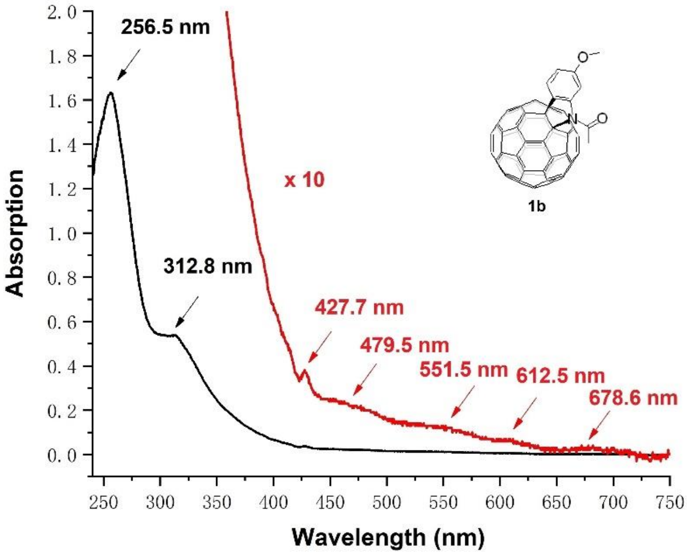

Figure S56 UV-vis absorption of compound 1b in $CHCl_3$

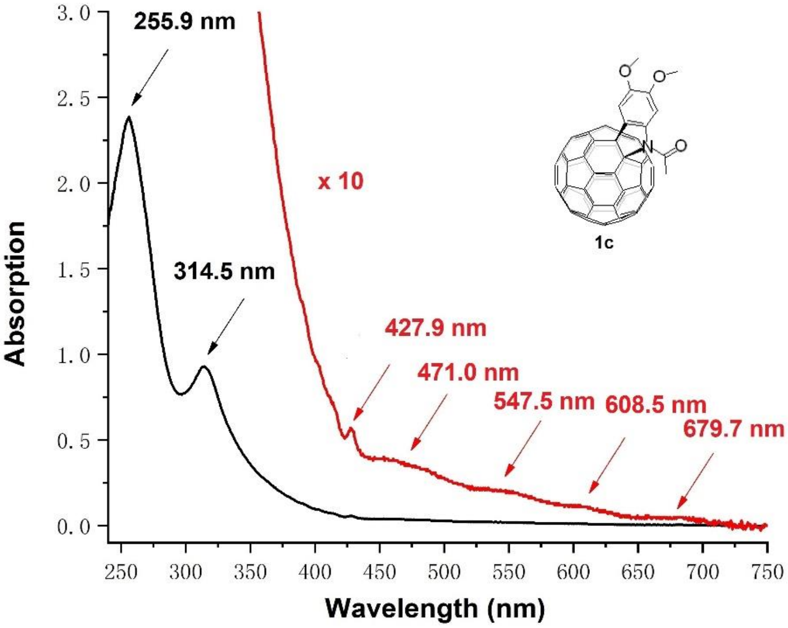

Figure S57 UV-vis absorption of compound 1c in $CHCl_3$

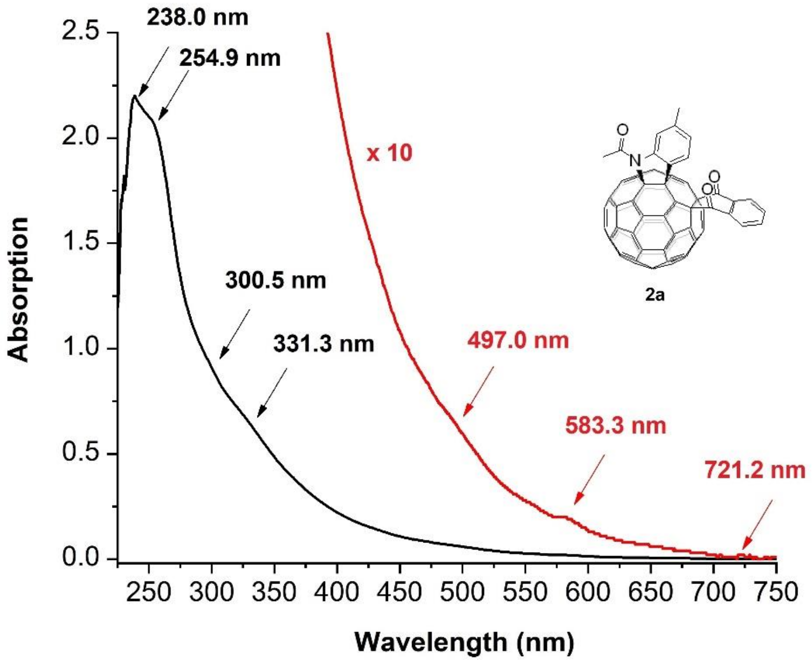

Figure S58 UV-vis absorption of compound 2a in $CHCl_3$

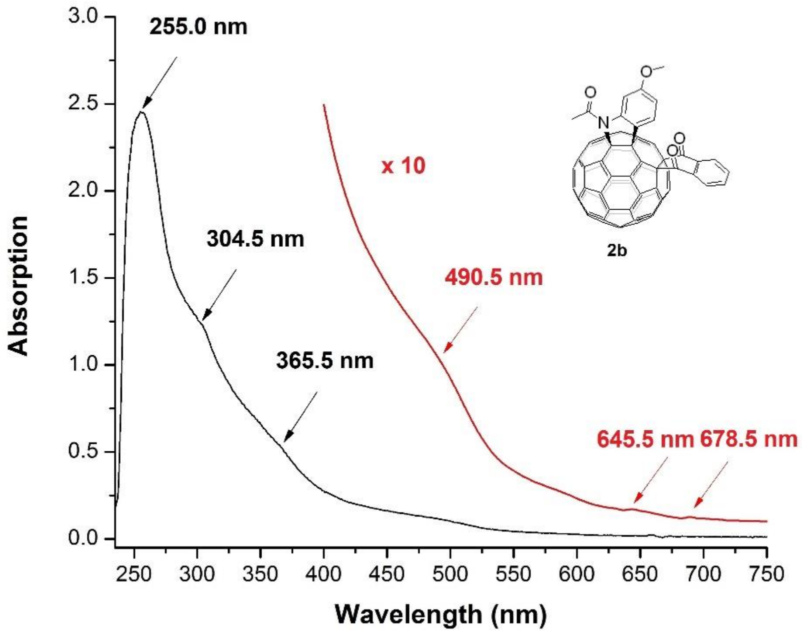

Figure S59 UV-vis absorption of compound 2b in $CHCl_3$

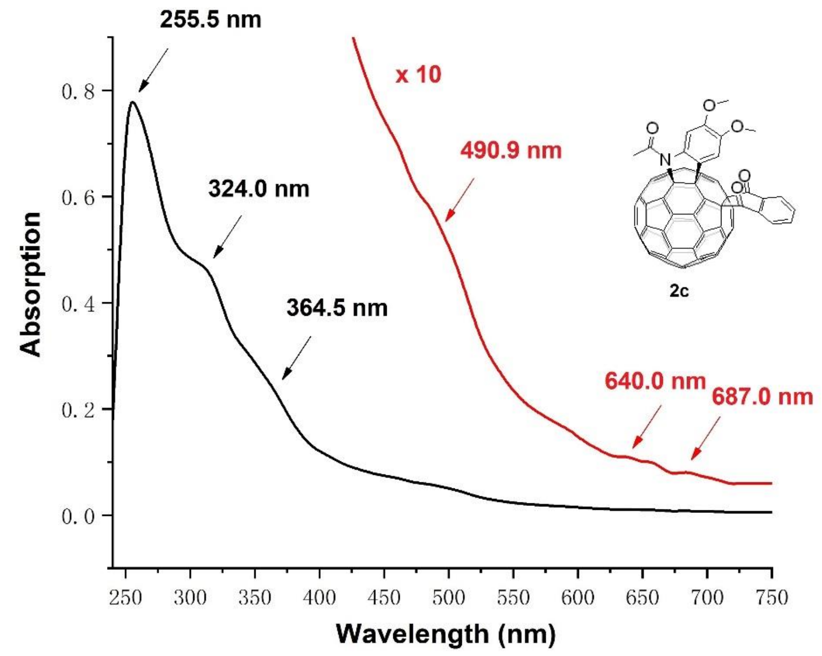

Figure S60 UV-vis absorption of compound 2c in $CHCl_3$

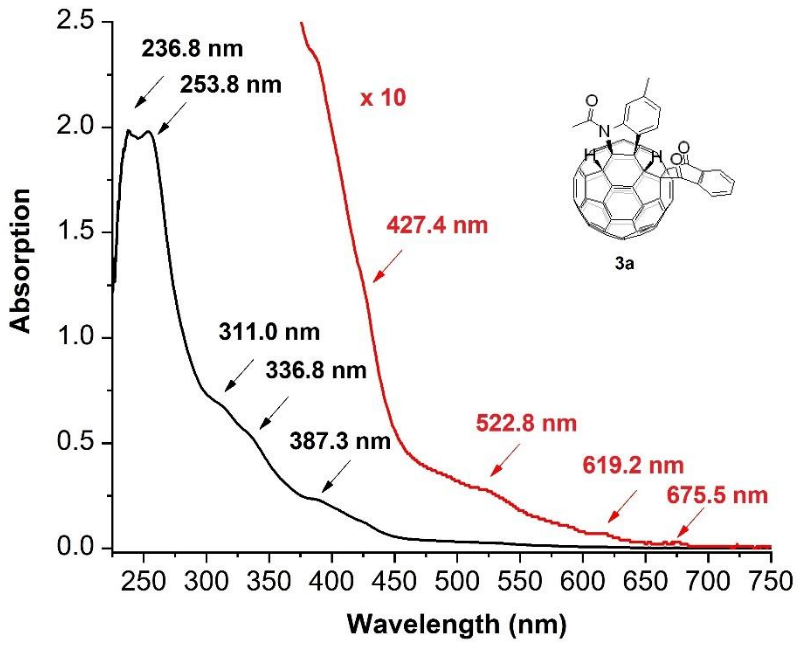

Figure S61 UV-vis absorption of compound 3a in $CHCl_3$

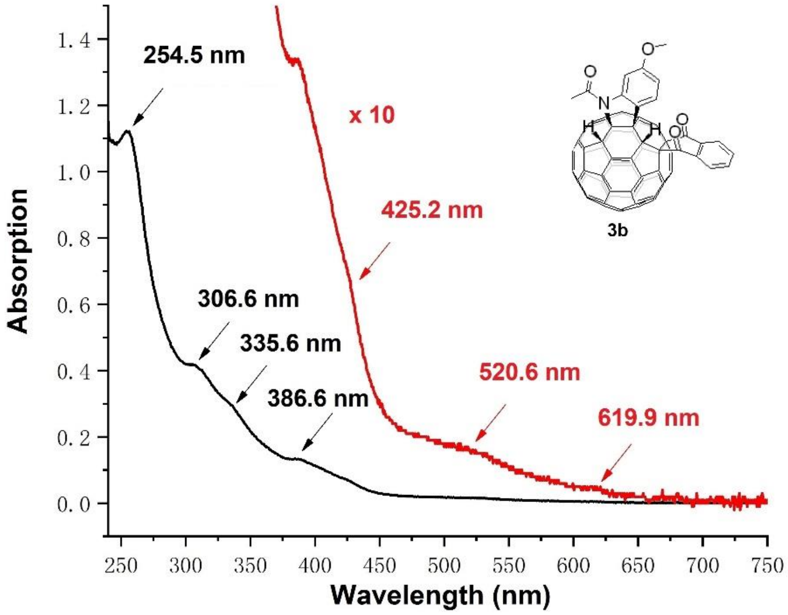

Figure S62 UV-vis absorption of compound 3b in $CHCl_3$

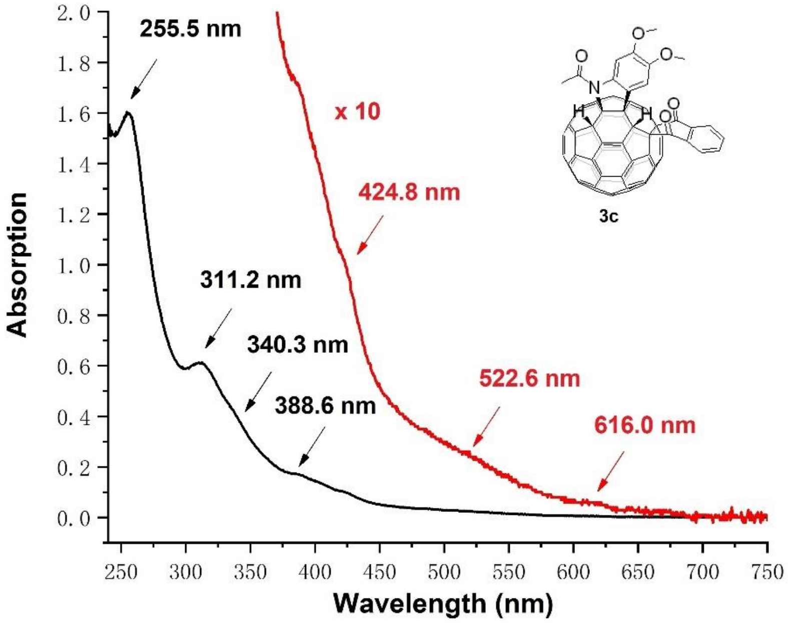

Figure S63 UV-vis absorption of compound 3c in $CHCl_3$

10. MALDI-TOF HRMS Spectra of Compounds 1b, 1c, 2a-c, and 3a-c

Trans-2-[3-(4-tert-Butylphenyl)-2-methyl-2-propenylidene]malononitrile (DCTB) was used as the matrix for MALDI-TOF HRMS spectra.

D:\Data\ZUCI\gc\181007\YXX-1\0_J7\1\1Ref

Comment 1

Comment 2

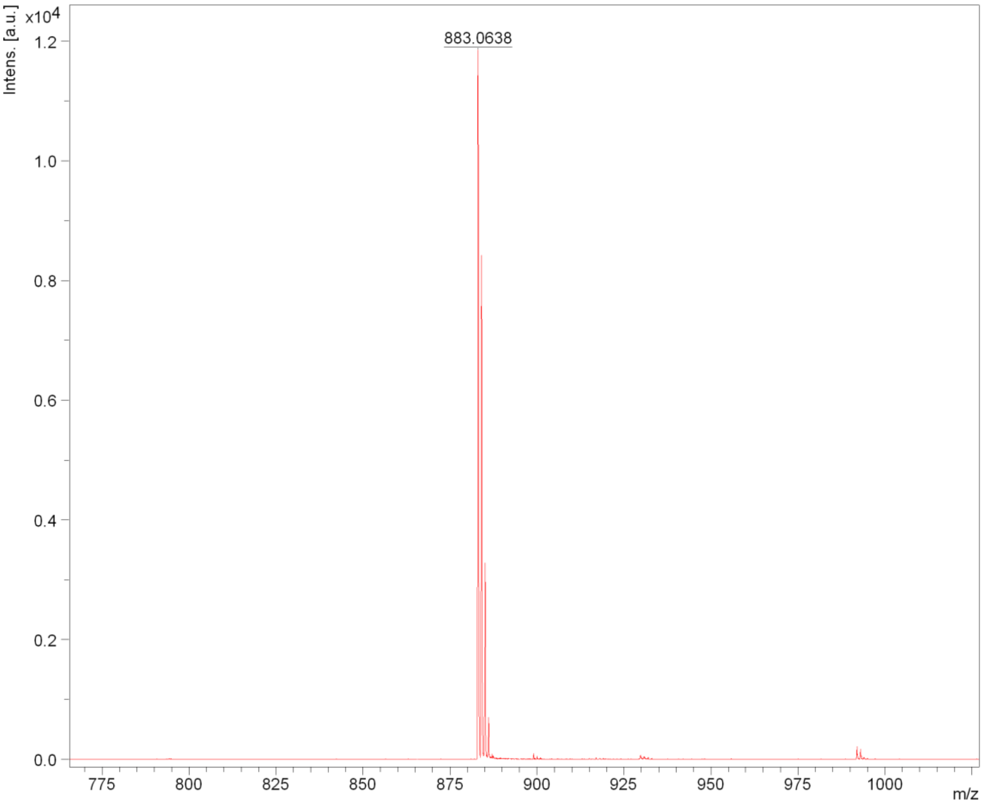

Acquisition Parameter

Date of acquisition	2018-10-07T11:12:30.859+08:00
Acquisition method name	D:\Methods\flexControlMethods\gc-RN_0-1200_Da.par
Aquisition operation mode	Reflector
Voltage polarity	NEG
Number of shots	500
Name of spectrum used for calibration	
Calibration reference list used	sample

Instrument Info

Bruker Daltonics flexAnalysis printed: 10/8/2018 3:22:17 PM

Figure S64 MALDI-TOF HRMS of Compound 1b

D:\Data\ZUCI\gc\181007\YXX-2\0_J8\1\1Ref

Comment 1
Comment 2

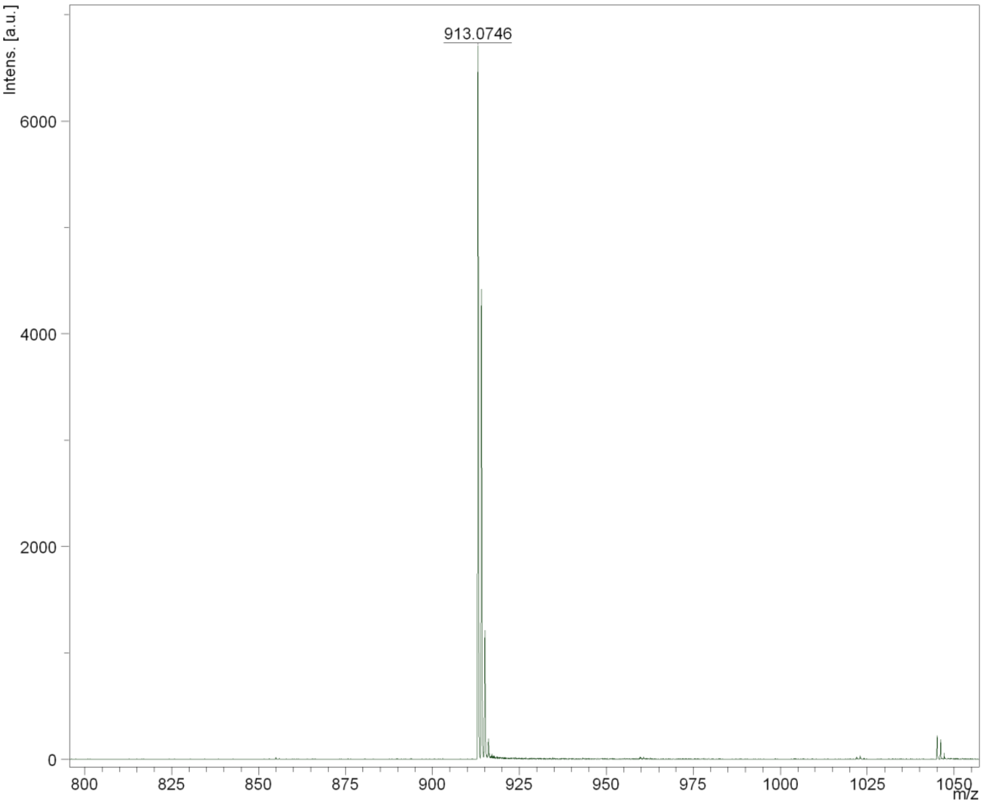

Acquisition Parameter

Date of acquisition	2018-10-07T11:12:43.655+08:00
Acquisition method name	D:\Methods\flexControlMethods\gc-RN_0-1200_Da.par
Aquisition operation mode	Reflector
Voltage polarity	NEG
Number of shots	500
Name of spectrum used for calibration	
Calibration reference list used	sample

Instrument Info

Bruker Daltonics flexAnalysis printed: 10/8/2018 3:23:28 PM

Figure S65 MALDI-TOF HRMS of Compound 1c

D:\Data\ZUCI\gc\181126\yxx-2c\0_F3\1\1Ref

Comment 1
Comment 2

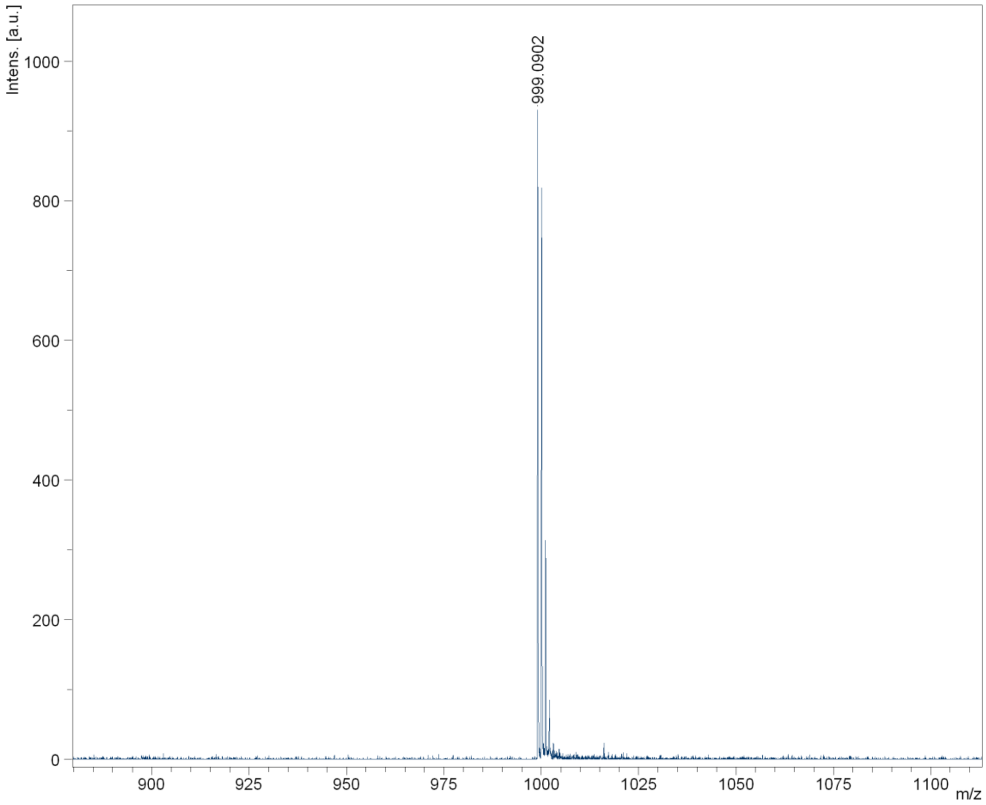

Acquisition Parameter

Date of acquisition	2018-11-26T21:58:38.140+08:00
Acquisition method name	D:\Methods\flexControlMethods\gc-RN_0-1200_Da.par
Aquisition operation mode	Reflector
Voltage polarity	NEG
Number of shots	500
Name of spectrum used for calibration	
Calibration reference list used	sample

Instrument Info

Bruker Daltonics flexAnalysis printed: 11/26/2018 10:20:29 PM

Figure S66 MALDI-TOF HRMS of Compound 2a

D:\Data\ZUCI\gc\161213\1MeO\0_B20\1\1SRef

Comment 1
Comment 2

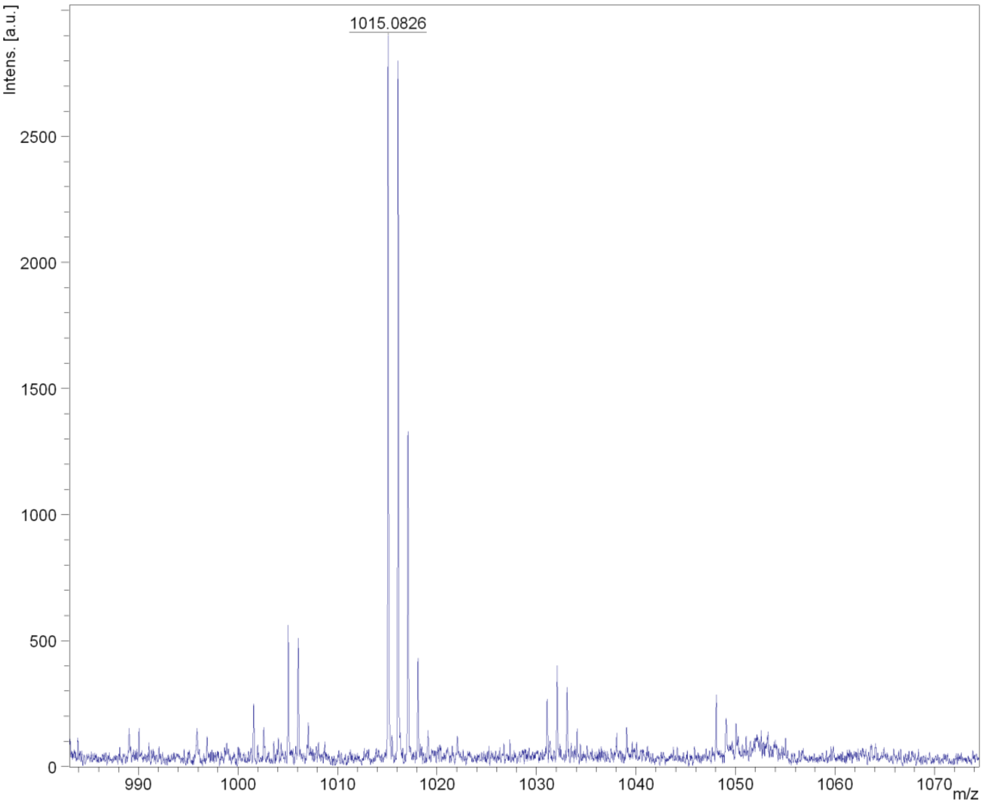

Acquisition Parameter

Date of acquisition	2016-12-13T14:45:15.156+08:00
Acquisition method name	D:\Methods\flexControlMethods\gc-RP_100-1500_Da.par
Aquisition operation mode	Reflector
Voltage polarity	POS
Number of shots	1500
Name of spectrum used for calibration	
Calibration reference list used	sample

Instrument Info

Bruker Daltonics flexAnalysis printed: 12/15/2016 2:00:20 PM

Figure S67 MALDI-TOF HRMS of Compound 2b

D:\Data\ZUCI\gc\161213\2MeO\0_B21\1\1Ref

Comment 1
Comment 2

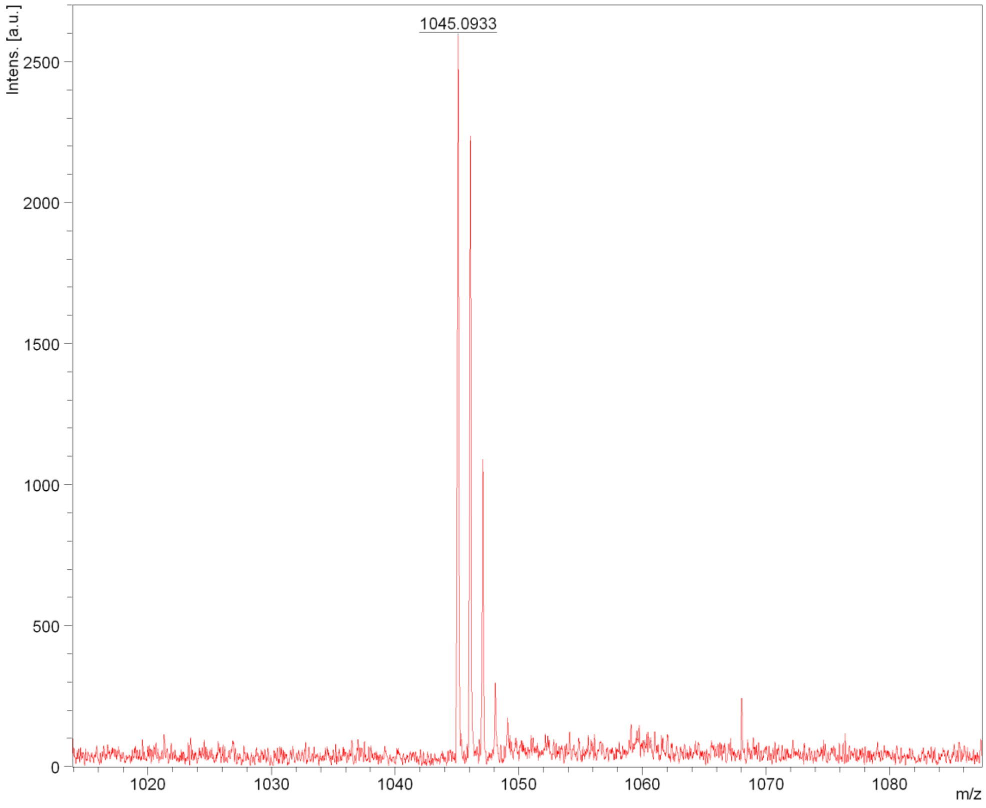

Acquisition Parameter

Date of acquisition	2016-12-13T14:36:44.109+08:00
Acquisition method name	D:\Methods\flexControlMethods\gc-RP_100-1500_Da.par
Aquisition operation mode	Reflector
Voltage polarity	POS
Number of shots	500
Name of spectrum used for calibration	
Calibration reference list used	sample

Instrument Info

Bruker Daltonics flexAnalysis printed: 12/15/2016 2:00:39 PM

Figure S68 MALDI-TOF HRMS of Compound 2c

D:\Data\ZUCI\gc\20190125\YXX-3C\0_B1\1\1Ref

Comment 1
Comment 2

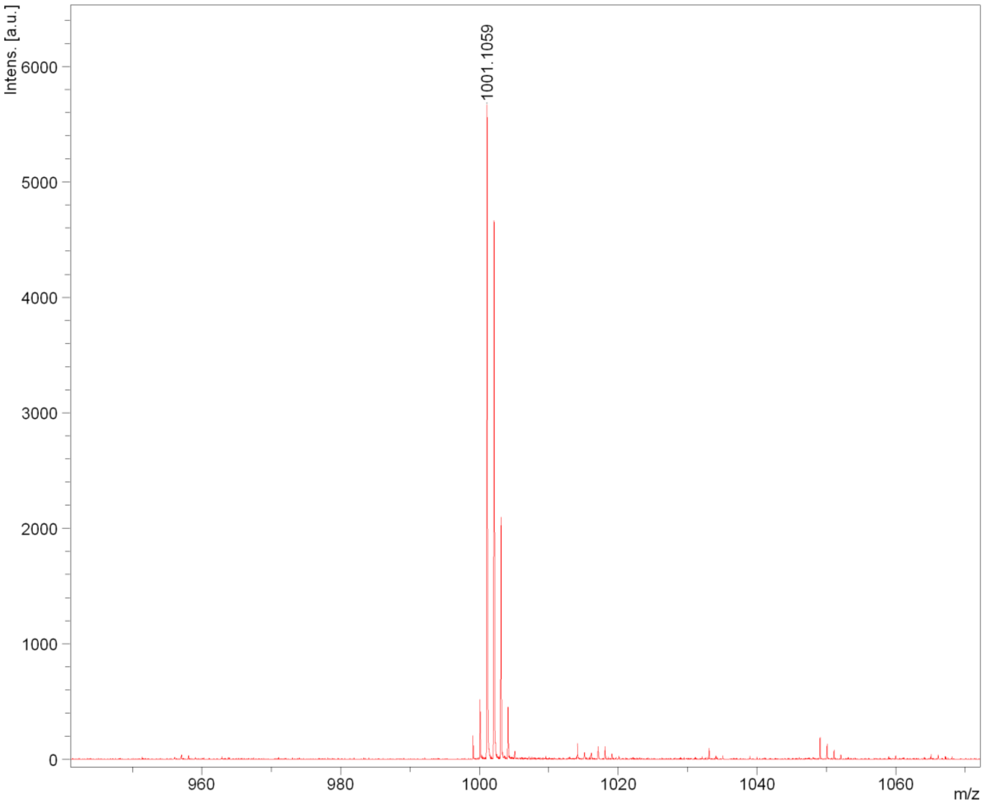

Acquisition Parameter

Date of acquisition	2019-01-26T15:03:14.765+08:00
Acquisition method name	D:\Methods\flexControlMethods\gc-RN_0-1200_Da.par
Aquisition operation mode	Reflector
Voltage polarity	NEG
Number of shots	500
Name of spectrum used for calibration	
Calibration reference list used	sample

Instrument Info

Bruker Daltonics flexAnalysis   printed: 1/26/2019 3:29:00 PM

Figure S69 MALDI-TOF HRMS of Compound 3a

D:\Data\ZUCI\gc\180324\yxx-multi\0_E21\1\1Ref

Comment 1

Comment 2

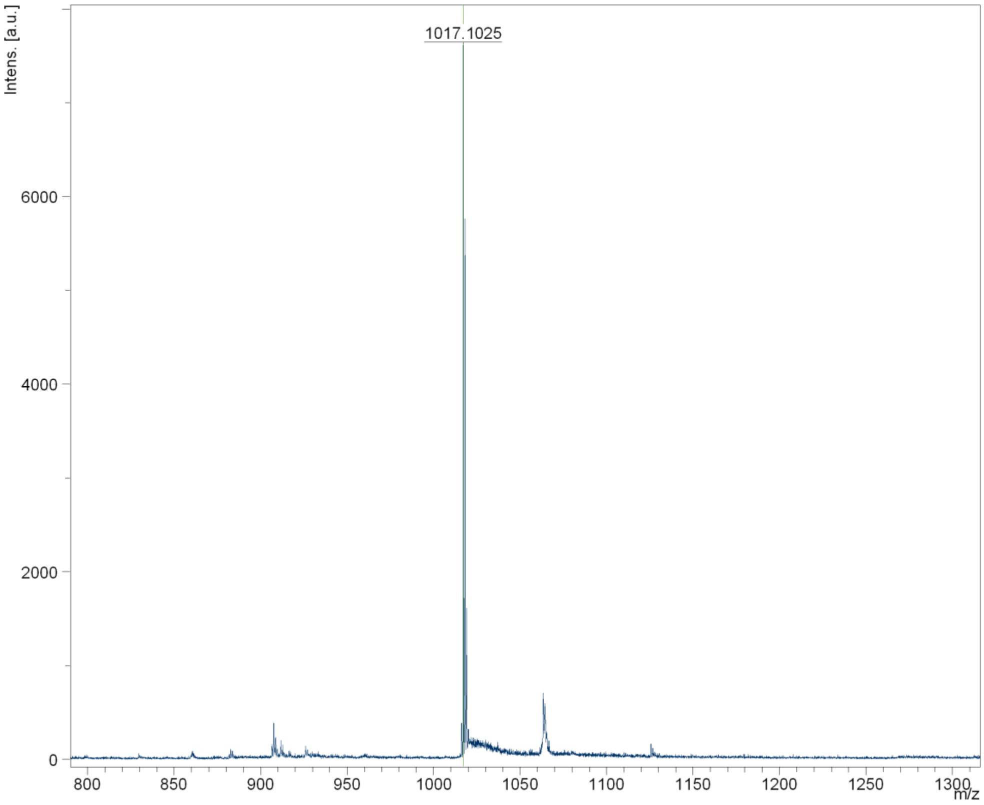

Acquisition Parameter

Date of acquisition	2018-03-24T16:25:35.445+08:00
Acquisition method name	D:\Methods\flexControlMethods\gc-RN_0-1200_Da.par
Aquisition operation mode	Reflector
Voltage polarity	NEG
Number of shots	500
Name of spectrum used for calibration	
Calibration reference list used	sample

Instrument Info

Bruker Daltonics flexAnalysis printed: 3/24/2018 4:46:57 PM

Figure S70 MALDI-TOF HRMS of Compound 3b

D:\Data\ZUCI\gc\180807\yxx-multi-2\0_I5\2\1Ref

Comment 1
Comment 2

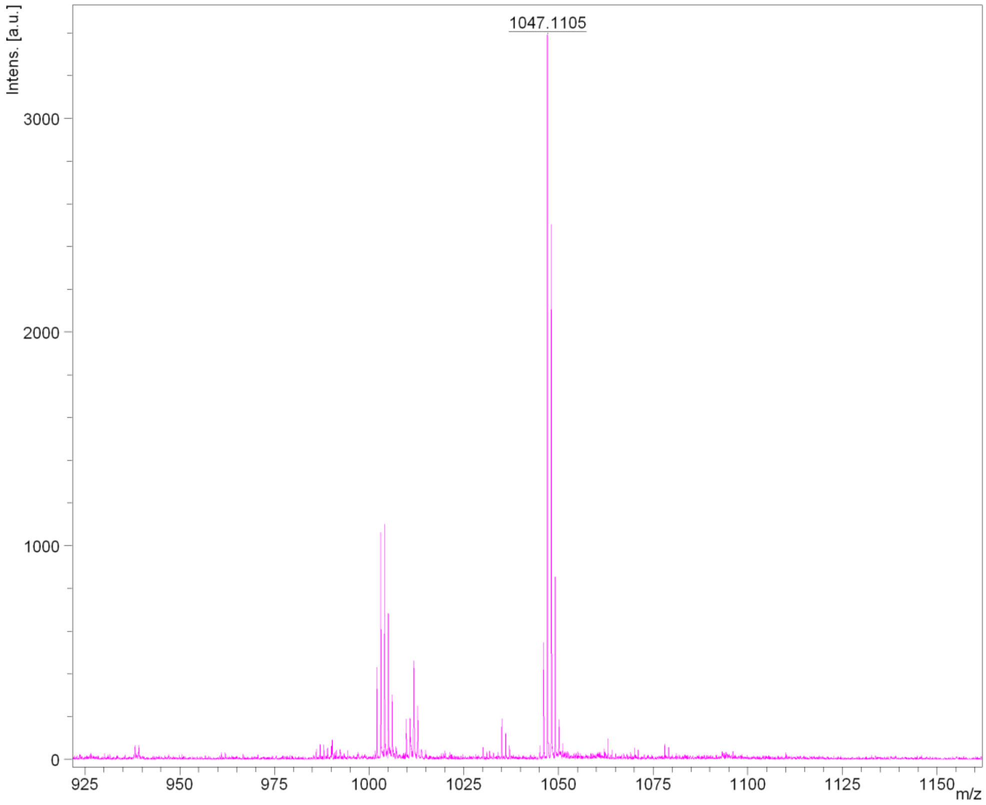

Acquisition Parameter

Date of acquisition	2018-08-08T00:47:29.105+08:00
Acquisition method name	D:\Methods\flexControlMethods\gc-RP_100-1500_Da.par
Aquisition operation mode	Reflector
Voltage polarity	POS
Number of shots	500
Name of spectrum used for calibration	
Calibration reference list used	sample

Instrument Info

Bruker Daltonics flexAnalysis printed: 8/8/2018 12:50:30 AM

Figure S71 MALDI-TOF HRMS of Compound 3c

11. References

1. H.-S. Lin, Y. Matsuo, J.-J. Wang, and G.-W. Wang, "Regioselective acylation and carboxylation of [60]Fulleroindoline via electrochemical synthesis." *Organic Chemistry Frontiers*, vol. 4, no. 4, pp. 603–607, 2017.
2. B. Zhu and G.-W. Wang, "Palladium-catalyzed heteroannulation of [60]Fullerene with anilides via C−H bond activation." *Organic Letters*, vol. 11, no. 19, pp. 4334–4337, 2009.
3. Y. Xiao and G. Wang, "A 1,2,3,4-tetrahydrofullerene derivative generated from a [60]Fulleroindoline: gegioselective electrosynthesis and computational study." *Chinese Journal of Chemistry*, vol. 32, no. 8, pp. 699–702, 2014.
4. T. Wei, S. Wang, F. Liu et al., "Capturing the long-sought small-bandgap endohedral fullerene $Sc_3N@C_{82}$ with low kinetic stability." *Journal of the American Chemical Society*, vol. 137, no. 8, pp. 3119–3123, 2015.
5. M. Yamada, Y. Muto, H. Kurihara et al., "Regioselective cage opening of $La_2@D_2(10611)\text{-}C_{72}$ with 5,6-diphenyl-3-(2-pyridyl)-1,2,4-triazine." *Angewandte Chemie International Edition*, vol. 54, no. 7, pp. 2232–2235, 2015.
